# Exploring Li growth modes in Li metal electrodes using X-ray nano-tomography

**DOI:** 10.1038/s41467-026-78182-9

**Published:** 2026-09-29

**Authors:** Josef Rizell, Victor Vanpeene, Martina Olsson, Jan Jamroz, François Cadiou, Hervé Manzanarez, Valentin Vinci, Olga Stamati, Julie Villanova, Sandrine Lyonnard, Aleksandar Matic

**Affiliations:** 1 Department of Physics and Astronomy, Chalmers University of Technology, Gothenburg, Sweden; 2 Univ. Grenoble Alpes, CEA, CNRS, Grenoble INP, IRIG, SyMMES, Grenoble, France; 3 Faculty of Physics, Warsaw University of Technology, Warsaw, Poland; 4 Wallenberg Initiative Materials Science for Sustainability (WISE), Department of Physics and Astronomy, Chalmers University of Technology, Gothenburg, Sweden; 5 Univ. Grenoble Alpes, CEA, Liten, DEHT, Grenoble, France; 6 European Synchrotron Radiation Facility (ESRF), 71 Avenue des Martyrs, CS 40220, Grenoble, France; 7 Univ. Grenoble Alpes, CNRS, Grenoble INP, 3SR, Grenoble, France

**Keywords:** Batteries, Batteries

## Abstract

To pave way for application of Li-metal electrodes in high energy density batteries the evolution of the 3D structures formed during charging needs accurate characterization inside working cells. The growth of porous, weakly connected structures is directly linked to the poor cycle life of Li-metal batteries. Here we present time-resolved analysis of the lithium metal structural evolution in 3D with sub-micron spatial resolution using in situ X-ray nano-tomography. This enables the growth mechanisms of entangled lithium morphologies to be studied both qualitatively and quantitatively. We identify distinct growth modes of needle-like and sheet-like structures, where segments are elongated at the tip, base, kinks or edges simultaneously with minimal coarsening. Different growth modes, and the transition between them, are linked to the initial growth of new grains and solid electrolyte interphase evolution, highlighting time resolved 3D morphological characterization as an avenue to understand the mechanisms of Li deposition.

## Abstract (web summary)

The growth of porous, weakly connected structures in Li electrodes is directly linked to the poor cycle life of Li-metal batteries. Here, the authors use in situ nano-tomography to track the evolution of these structures and understand their growth mechanism.

## Introduction

As a result of the high capacity (3860 mAh/g) and low redox potential (–2.79 V vs a standard hydrogen electrode in propylene carbonate)^1^ of Li, cells with energy densities well above those of conventional lithium-ion batteries can be realized by replacing the graphite electrode with Li metal^2^. In a Li-metal cell, lithium is electrochemically plated onto the negative electrode during charge and stripped during discharge. Ideally, the plated Li metal would form a dense and uniform film^3^, but in practice, it tends to plate and strip heterogeneously, resulting in low reversibility and risks of short circuiting the cell^4^. Branched or porous Li structures expose large surface areas towards the electrolyte, driving the loss of active material through exacerbated side reactions with the electrolyte^5,6^. During stripping, the electrical connection between fragile lithium structures and the current collector can also be broken, further aggravating capacity decay^3,7^.

The morphology of the deposited lithium metal will depend on several factors^8^. Branched, fractal structures can form due to ion transport limitations in the electrolyte at high current densities or low temperature^8,9^, analogous to what is observed for metals like Cu and Zn^10^. However, lithium deposition differs from electrodeposition of several other metals as it is deposited outside the electrochemical stability window of the commonly used liquid electrolytes. At low potentials electrolyte components are reduced at the negative electrode until a passivating solid electrolyte interphase (SEI) layer forms, through which the Li-ions must be transported to reach the electrode^10^. The nature of the SEI, e.g., ionic conductivity and mechanical strength, will play a crucial role for the final morphology^11^. Homogeneous deposition is expected below a mechanically strong SEI, whereas imperfections or cracks in the SEI result in hotspots for deposition with heterogeneous growth as a result, e.g. as needle-like structures^12,13^. The further evolution of such structures during deposition will also depend on the SEI formed on them, with branching suggested to occur at imperfections in the SEI^14^.

To obtain a detailed mechanistic understanding of how different lithium morphologies form, the evolution of structures at different stages of deposition needs to be followed, requiring non-destructive in situ characterization. Historically, insights on lithium growth modes have been mainly derived from in situ optical microscopy^12,15,16^. Thin Li whiskers or needles, with sharp kinks, have been reported to grow from the base^16^, rationalized as extrusion of lithium through cracks or imperfections in the SEI^12^. In parallel, lithium whiskers are also observed to experience growth from tips or kinks^15–17^. However, details are difficult to discern with the low spatial resolution of optical microscopy. Using transmission electron microscopy (TEM) significantly higher resolution is obtained, for instance allowing transitions between 1D whisker and multidirectional growth at lower overpotentials or at mechanical constraints to be observed^14,18,19^. Branching of Li deposits could also be studied by TEM and attributed to crack formation in the SEI-layer^14^. While TEM provides excellent spatial resolution, the cell configuration is far from ideal only allowing very thin two-dimensional structures in a small field of view to be tracked. This limits investigations of lithium deposition to relatively short timescales, as structures soon start growing outside the field of view.

To draw statistically relevant conclusions on the growth of lithium deposits, measurements combining high spatial resolution and a large field of view are highly desirable. In addition, 3D imaging is essential to understand the complexity and internal structure of three-dimensional lithium morphologies often formed during electrodeposition^20,21^. We have previously shown that Li deposition and dissolution inside a working cell can be tracked in 3D using X-ray micro-tomography^22^. Although 3D structural visualization and quantitative analysis were demonstrated, the connectivity of deposits and their fine structure could not be fully captured with the spatial resolution (≈1–2 µm) in that experiment. To capture the growth of lithium with sub-micron spatial resolution, we here apply in situ X-ray nano-holo-tomography. Through phase contrast this method provides the high sensitivity needed to distinguish between weakly attenuating materials, allowing growth of lithium metal during electrodeposition to be followed in a working cell with a high spatial resolution of 150 nm (Supplementary Notes 1–2 and Figs. S1–3). The high-resolution 3D data enables visualization of the individual lithium structures in the highly entangled mossy lithium^20^, classification of different types of Li morphologies and quantitatively tracking their evolution over time. We identify two distinct classes of structures, needle-like and sheet-like lithium, and observe that both types grow through a complex mechanism where segments are elongated at the tip, base, kinks, and edges of the structures simultaneously with minimal coarsening. Connected deposits often contain both types of lithium structures, with SEI evolution playing a key role in transitioning between them.

## Results and discussion

### Identification of structural building blocks

**Fig. 1:**
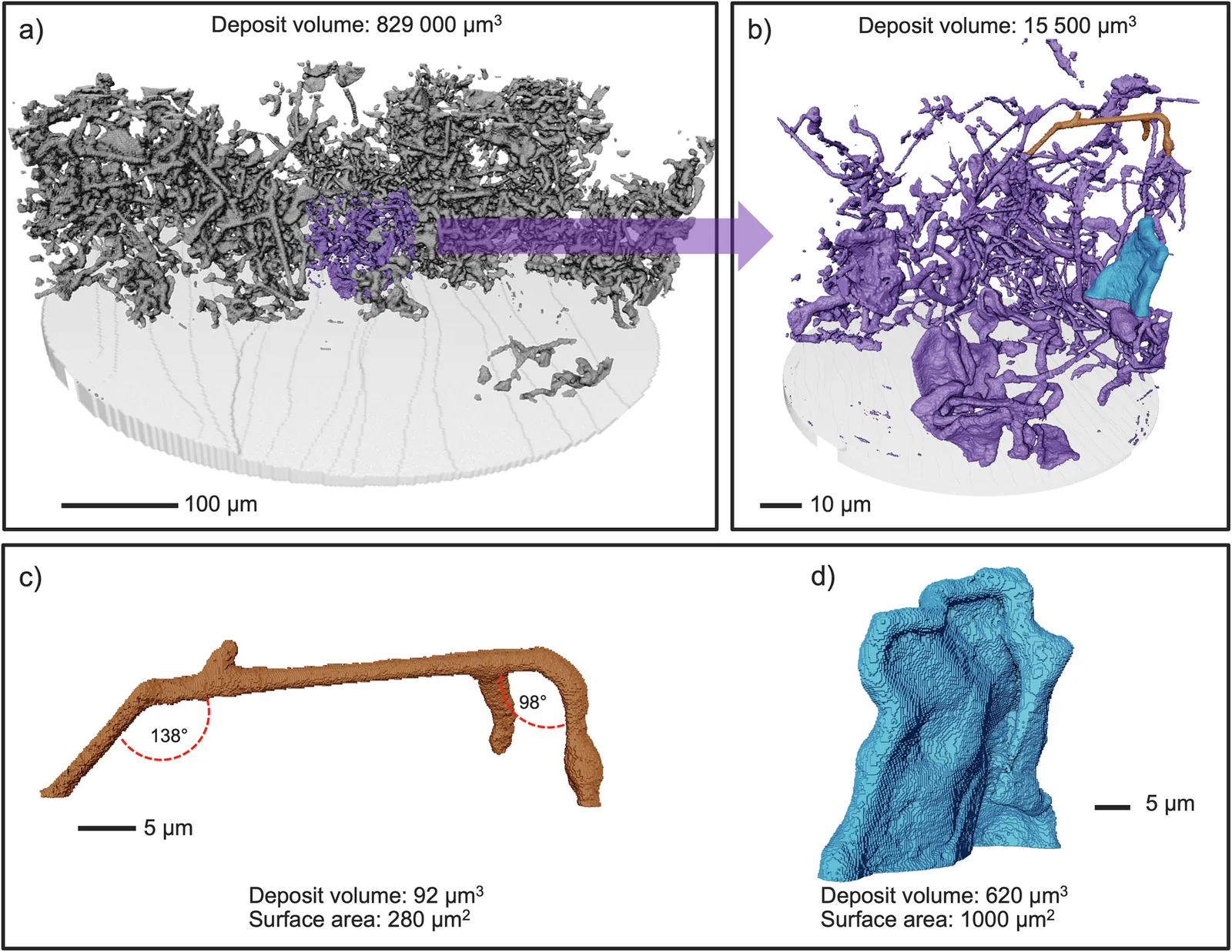
Hierarchical 3D imaging of lithium structures from electrode scale to individual building blocks. **a** Volume rendering of segmented Li (gray) after deposition at 1 mA/cm^2^ for 1 h, from in situ nano-tomography measurements at 257 nm voxel size. The Li in the region where nano-holo-tomography was performed is shown in violet. **b** Volume rendering of segmented data from a nano-holo-tomography measurement with 50 nm voxel size and data binned to 100 nm voxel size for the analysis, where examples of a lithium needle (brown) (**c**) and a lithium sheet (blue) (**d**) are highlighted.

Results from in situ X-ray nano-tomography during galvanostatic plating of Li metal in symmetric cells with a pressure free configuration (Fig. S1 and Supplementary Note 3) are shown in Fig. 1. Measuring without applied pressure constitutes an idealized case, or model system, simplifying the analysis of the driving forces controlling the evolution of different morphologies during lithium deposition. Furthermore, local differences in pressure are common in both coin and pouch cells^23–25^, and it is thus important to understand how lithium grows also in regions with little or no pressure.

In the experiment, we adopt a hierarchical approach combining large field-of-view imaging and zoomed-in high-resolution scans to visualize details of the structure. Overview tomograms at low resolution with a 526 µm field of view (FOV) (Fig. 1a) are complemented with nano-holo-tomography measurements, with a 102 µm FOV, with a high spatial resolution (see Fig. 1b and Supplementary Notes 1–2). The overview image reveals that a highly porous lithium morphology, with entangled structures, has been formed after deposition at 1 mA/cm^2^ for 60 min. This morphology stands in contrast to both (i) classical dendrites, which are fractal self-similar structures forming due to ion depletion in the electrolyte (Supplementary Note 4)^9,26^, as well as (ii) ideal bulk lithium. The applied current is too low to cause ion depletion with the cell geometry and electrolyte used, explaining the absence of fractal structures (Supplementary Note 4).

From the high-resolution nano-holo-tomography images, two fundamental building blocks of the morphology can be identified (Figs. 1c, d and S4), thin needle-like structures, and wrinkled sheet-like structures (Figs. 1d and S5a). The needle-like structures, with a typical thickness of 1–2 µm, abruptly change growth direction at kinks (Fig. 1c). Similar structures have also been found in ex situ scanning electron microscopy (SEM) images on electrodes from deposition experiments in larger cells (Fig. S5b) and cryo-TEM has previously shown that kinked Li needles, or whiskers, are (single) crystalline^27^. Here, using holo-tomography, a faceted needle with a hexagonal cross-section and around 2 µm wide facets could for instance be identified (Fig. S6), which is an expected shape for a lithium needle growing along the <111> direction^27^.

Three main explanations for the needle growth during electrodeposition have previously been proposed. (i) The potential of zero charge is much higher than the reduction potential. This creates a large negative surface charge during deposition, making it thermodynamically favorable to increase the surface area through needle growth in order to distribute the surface charge^28^. It has also been argued that the negative surface charge localizes to the tips/protrusions, further increasing deposition at these points^29^. (ii) Inhomogeneous deposition beneath the SEI layer also creates stress and, eventually, the SEI layer cracks and lithium metal is extruded through the crack as a needle^12,18^. (iii) Li crystallites are anisotropic, with better attachment kinetics at some crystal face, promoting one-dimensional growth^4,17^. For needles to form this way lithium atoms at the surface need sufficient time to rearrange into the thermodynamically favored sites, requiring fast surface diffusion^30^. While stress-driven extrusion of lithium (ii) could explain the initiation of needle-type structures, including branching and would favor base growth, Fig. S7 shows that segments in the middle of lithium needles elongate and branch during deposition. This indicates that a negatively charged surface (i) and/or crystal anisotropy (iii) should also be important drivers for the elongation of needle segments during lithium growth.

The sheet like structures have a similar thickness as the needles (Figs. 1d and S4b). Reports discussing sheet-like Li deposits are uncommon in literature, but several publications show SEM images of similar structures in a range of electrolytes (Supplementary Note 5) without discussing them in detail^31–36^. A sheet-like morphology has been associated with non-crystalline deposition^37^ and amorphous lithium deposits have previously been observed in TEM experiments^38,39^. At lower current densities, more amorphous nuclei are suggested to form, which then evolve into sheet-like structures with low crystallinity^37^. Considering the proposed dependence of deposit shape on the crystallinity of the initial structures, the coexistence of needle like and sheet like lithium structures (Fig. 1) could be a signature of local variations in how new lithium grains form. This may result from local current density variations caused by SEI inhomogeneity and its evolution.

### Evolution of 3D lithium structures

**Fig. 2:**
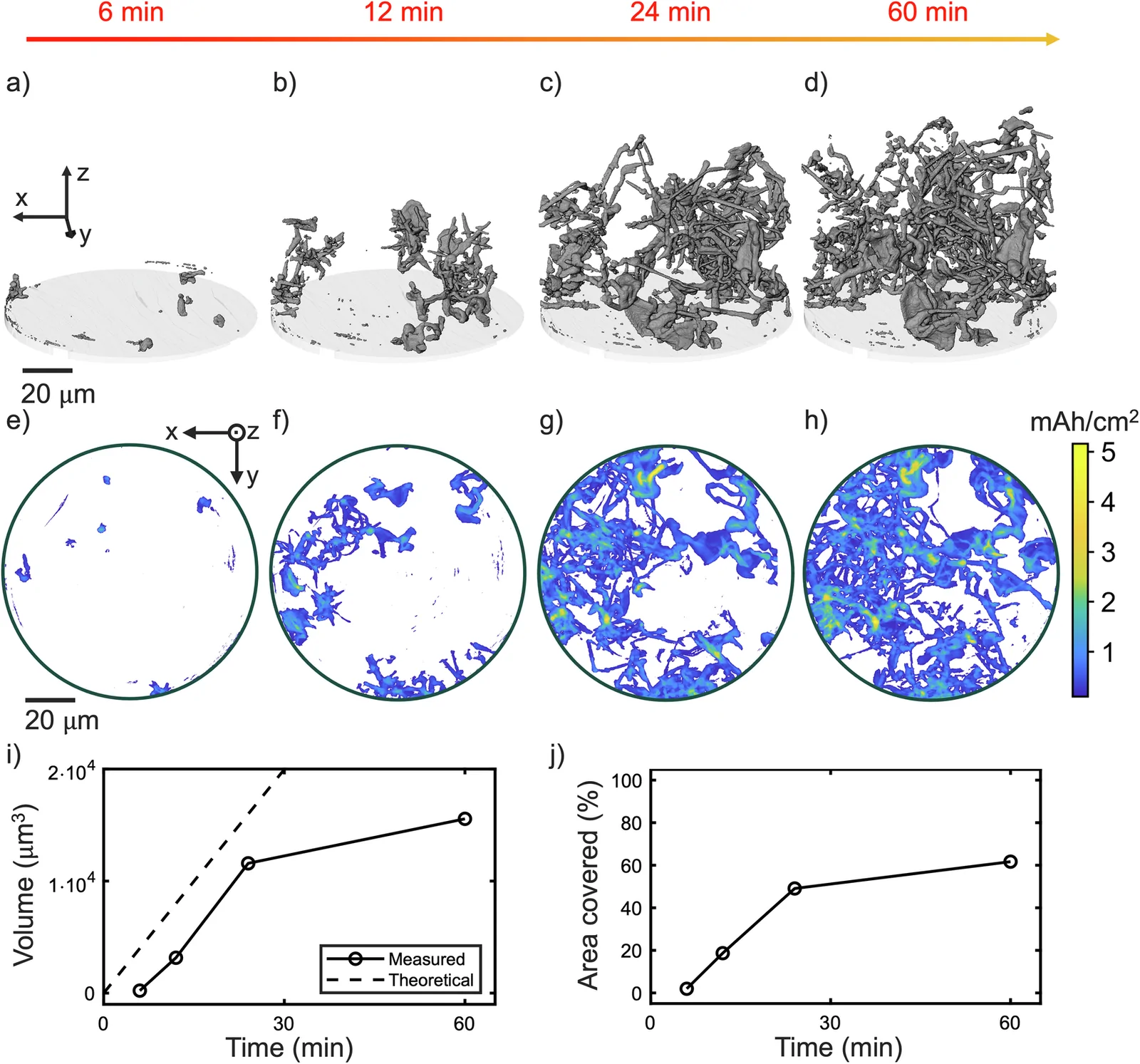
Evolution of lithium deposition. Segmented Li structures after 6 min (**a**), 12 min (**b**), 24 min (**c**) and 60 min (**d**) of deposition at 1 mA/cm^2^. Areal capacity of deposited Li above each point in the x-y plane of the electrode shown as a heat map after 6 min (**e**), 12 min (**f**) 24 min (**g**) and 60 min (**h**) of deposition. **i** Volume of deposited Li as a function of time. Dashed line shows the expected Li volume from the galvanostatic deposition. **j** Electrode area covered with deposited Li (in a projection along the *z*-axis, extracted from **e** to **h**).

The formation of the structures shown in Fig. 1 was tracked step by step during galvanostatic deposition (Fig. S8) to further understand the growth mechanism. The plating was interrupted for holo-tomographic acquisition at four points during the deposition. Initially, the growth of lithium is localized to a few distinct points on the electrode (Fig. 2a, tomogram taken 6 min into deposition). From these initial locations the structures gradually branch out and grow, forming the mossy lithium morphology (Fig. 2b–d, 12–60 min of deposition). The volume of the deposited Li can be quantified and tracked over time (Fig. 2i). In the first stages of deposition, up to 24 min, the volume of deposited Li increases linearly with time, as expected for a galvanostatic process, and it closely follows the Li volume expected based on homogeneous deposition across the entire electrode at the applied current density (1 mA/cm^2^). However, in the later stages the volume of deposited Li increases significantly slower. This can partially be explained by Li structures growing above the FOV.

To obtain further quantitative insight into the growth modes, the Li distribution across the electrode can be analyzed by projecting the deposited Li onto the electrode surface. This yields a heat map showing the areal capacity of Li deposited above each point on the electrode (Fig. 2e–h, for details, see “Methods” section). As expected, both areal capacity of deposited lithium and areal coverage increase during deposition (Fig. 2j). This indicates that the growth of Li is multidirectional since structures growing vertically increase the local areal capacity, while horizontal structural components increase areal coverage. Only a few new intersection points form between the bulk Li electrode and deposited Li between 6 and 60 min (Fig. S9), showing that the initial structures are the main locations for further deposition. This can be explained considering that a native surface layer will always cover the pristine bulk electrode^33,34^. The initial structures formed during deposition will pierce through this layer and form an interphase only through reactions with the electrolyte, which is expected to be more conductive, promoting further deposition on existing structures rather than the formation of new ones from the bulk^40,41^.

**Fig. 3:**
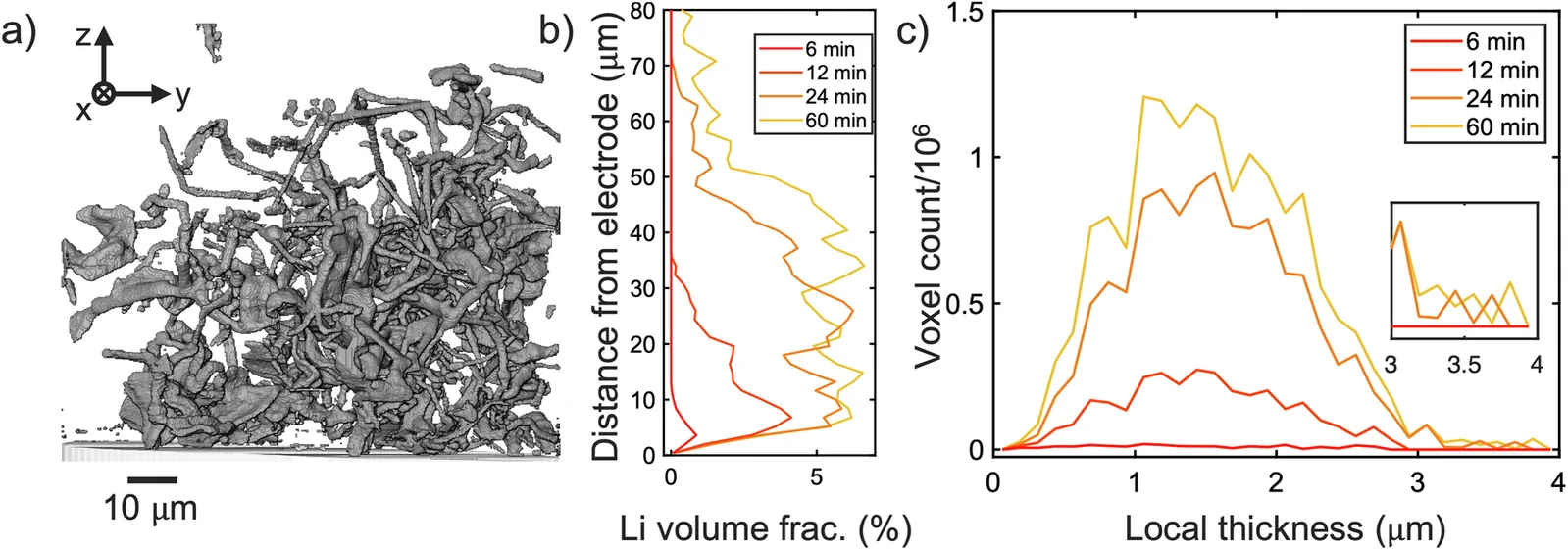
Quantification of Li deposition (1 mA/cm^2^) over time. **a** Side-view rendering of segmented Li after 60 min of deposition. **b** Volume fraction of deposited Li in a cross-sectional slice through the deposited structure as a function of the distance from the bulk electrode (along the z-axis in **a**). **c** Histogram of the local thickness evolution of segmented Li structures.

The Li structures formed after 60 min of deposition (Fig. 3a) extend beyond the FOV (80 μm into the electrolyte) and appear sparser further away from the electrode. To track the evolution of the structures, the volume fraction of Li was calculated as a function of the distance from the electrode at different deposition times (Fig. 3b). After 6 min, the lithium structures extend less than 12 microns into the electrolyte. With continued deposition they extend further, but the amount of Li close to the electrode surface also increases. To clarify where Li is added to the structures during deposition, the local thickness of the lithium structures is calculated at each time point (Fig. 3c, for details see methods section). The average local thickness increases only slightly, from 1.40 μm (σ = 0.71 μm) to 1.55 μm (σ = 0.62 μm) between 6 and 60 min of deposition (Fig. S10). The almost constant average local thickness, and lack of new growth sites on the bulk electrode (Fig. S9), show that Li grows mainly through branching, elongation of branches or planar growth, without significant coarsening of the structures. However, the very small amount of thicker Li (around 3 μm) that appears in later stages of deposition, indicates that some coarsening of existing structures over time cannot be excluded.

**Fig. 4:**
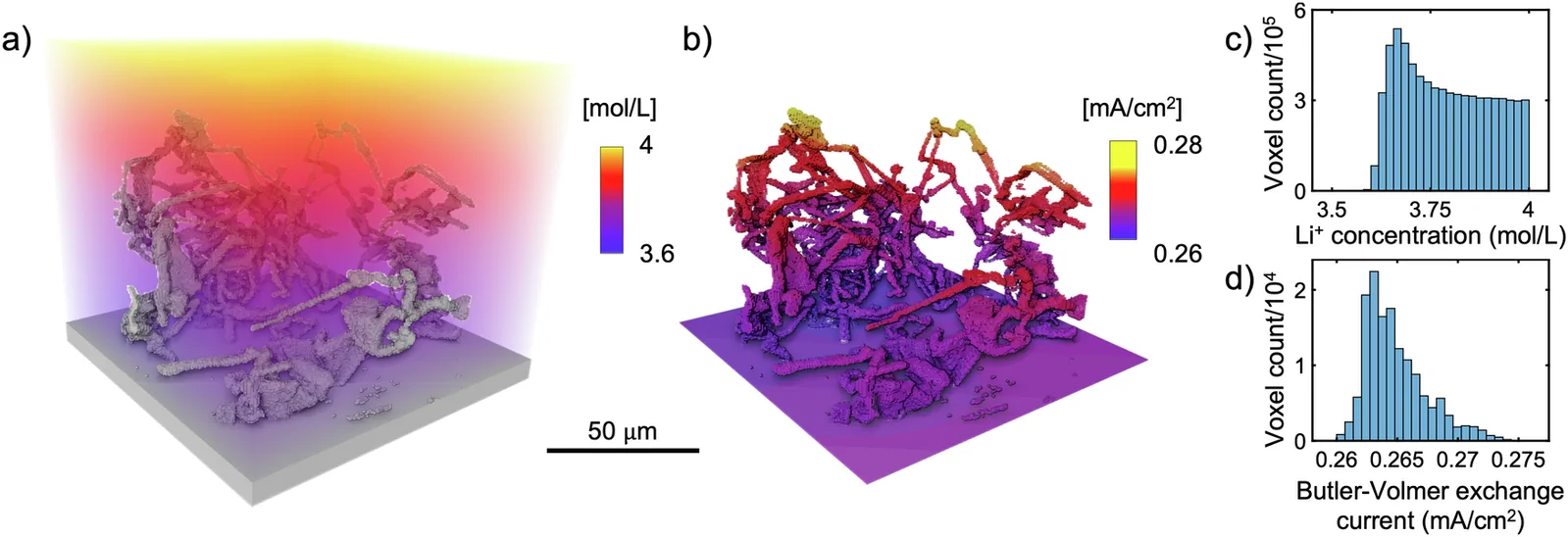
3D electrochemical simulations, based on Li morphology after 24 min deposition at 1 mA/cm^2^. **a** Li-ion distribution in the electrolyte, visualized with a semitransparent colormap. Gray structures correspond to the Li metal. **b** Local Butler-Volmer exchange current density distribution across the electrode surface. **c**, **d** Histograms based on the data in **a**, **b**.

To elucidate the growth mechanism, the equilibrium Li-ion concentration and local current densities were obtained from electrochemical simulations based on the actual 3D-structures obtained from tomography (Figs. 4, S11–S13, and Supplementary Note 6). Assuming a homogeneous interfacial resistance across the electrode, a Li-ion concentration gradient from the top of the sample to the bulk electrode is predicted, with smooth variations and no significant changes around edges or endpoints (Fig. 4a, c). This ion concentration gradient would cause slightly higher Butler-Volmer exchange current density at the structures extending the furthest into the electrolyte (Fig. 4b, d), but generally the variations are small, less than 10%. Clearly, neither the differences between needles and sheets or the emergence of either shape, can be due to enhancement of electrical fields at tips or Li concentration gradients. Instead, effects such as crystal anisotropy and differences in interfacial resistance across the electrode, not accounted for in the simulation, must be driving the evolution of the electrode morphology.

**Fig. 5:**
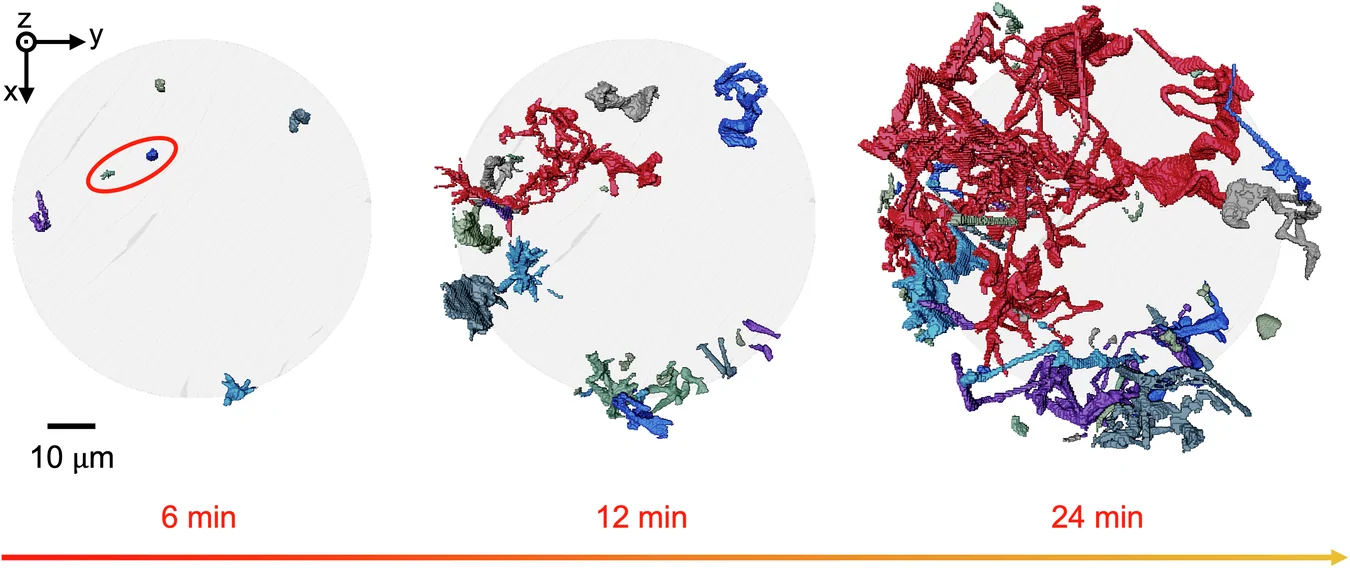
Connectivity of Lithium deposits. Volume rendering of segmented Li deposits, on top of the bulk electrode (light gray), at different times of deposition (1 mA/cm^2^). Li deposits that are not connected appear in different colors.

From the 3D images, we can also access the connectivity of the evolving deposits (for details, see “Methods” section). Figure 5 shows the largest connected Li deposits ($\ge 1.25\,$µm^3^), rendered in different colors. Initially (6 min) only small, isolated deposits appear, but with further deposition some of the deposits that nucleated in different spots (inside red oval) are now connected (shown in red), and the apparent connectivity increases with deposition. To determine if different branches coalesce^15^ or if they just come very close or touch without being attached, the two initial deposits circled in Fig. 5 were investigated in detail (Fig. S14). A branch connecting these deposits after 24 min moves with further deposition (60 min), showing that the deposits were in fact not attached. Still, the possibility of different branches coalescing during deposition remains an open question. Several closed loops are found in the analyzed volume (Fig. S15), which could be a sign of coalescence, but this data is not sufficient to determine if the loops generally stay connected during continued growth.

### Growth mechanisms of individual lithium structures

**Fig. 6:**
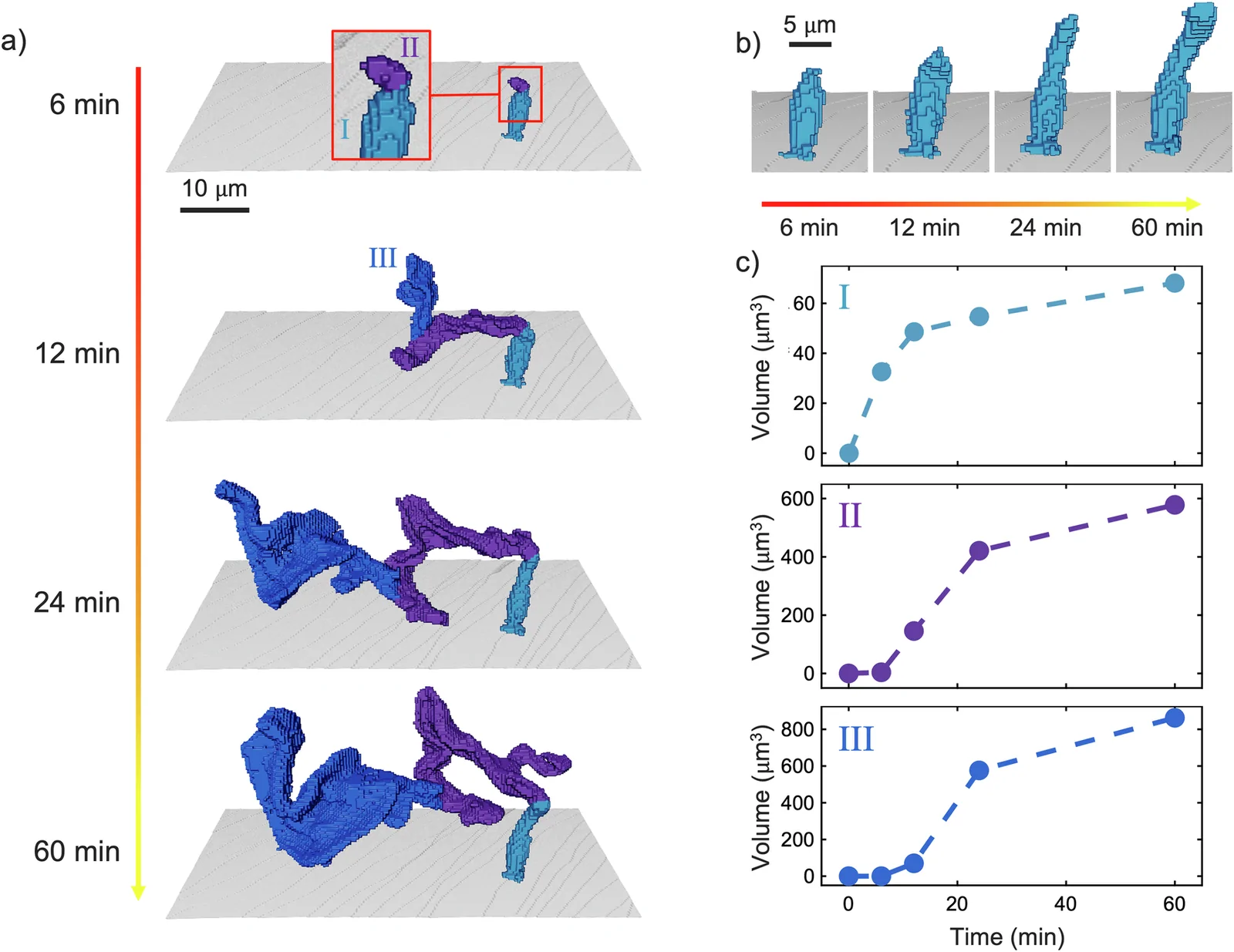
Evolution of a Li deposit. **a** 3D renderings of the same segmented deposit after 6, 12, 24 and 60 min of deposition at 1 mA/cm^2^. For clarity some lithium connected to this structure is not shown. The full structure is shown in Fig. S18. **b** Evolution of the base of the structure (I in a) between 6 and 60 min of deposition. **c** Volume evolution of the different parts of the structure in **a** (I, II and III shown in light blue, purple and dark blue respectively). The bulk Li electrode is shown in gray.

To access the lithium growth mechanisms, a specific structure can be isolated in the segmented volumes and features of interest tagged (I, II and III in Fig. 6a). The growth of the structure follows a complex sequence of steps, potentially associated with distinct growth mechanisms. The growth of the structure in Fig. 6a starts like a needle protruding vertically from the bulk Li electrode (6 min, segment I), with a small kink visible at the tip (segment II). With further deposition (12 min), segment II has grown in the horizontal direction and another kink has formed creating a new segment in the vertical direction (III). The tip of segment III has already started to expand and as deposition continues, it grows and gradually folds to form a wrinkled sheet. A closer look at segment I (Fig. 6b) also reveals a continuous evolution of the base of the structure. This segment keeps growing throughout the 60 min of deposition, affirmed by the increased volume of this segment (Fig. 6c), pushing the rest of the structure away from the electrode surface.

All three segments grow simultaneously, but after rapid growth of the initially formed segment (I), the growth is transferred to the freshly formed segments (II and III) as they appear (Fig. 6c). Branching is also observed, with a new branch extending from the side of segment II after 12 min, growing both longer and thicker with further deposition (Fig. S16a). Meanwhile, the sheet-like segment (III) expands (Fig. S16b), implying a growth from its edge. Altogether, this example shows multiple segments growing simultaneously, which reflects simultaneous Li growth at the base, tip, kinks and other parts of the structure. This stands in contrast to the sequential growth of needle segments through extrusion from the base proposed by Kushima et al.^18^, underlining that needle growth occurs not only from the base, but rather through concurrent growth of connected segments, proposed for instance by Steiger et al.^16^. The fast growth of newly formed segments points to preferential deposition on freshly formed structures, where the SEI-layer is expected to be thin and highly conductive. One can note that the growth rate significantly decreases in the later stages of deposition for all structures, which can be linked to the increasing total electrode surface area, effectively reducing the average local current density, and the formation of a thicker more resistive SEI on aging parts of the structure, redirecting deposition towards freshly formed structures. This explanation is supported by electrochemical impedance spectroscopy (EIS) measurements (Fig. S17 and Table S2). During deposition, when more fresh structures are continuously formed, the overall electrode impedance decreases as the electrode surface area increases. However, the electrode impedance increases when held at open-circuit voltage (OCV) after deposition, highlighting that the SEI becomes more resistive as it ages. This can be directly connected to the preferential deposition on newly formed structures (Fig. S9). Note that the fast growing freshly formed structures can both be close to and far from the bulk electrode since new branches can grow in many different directions, as for instance illustrated by the growth of the structure in Fig. 6a.

**Fig. 7:**
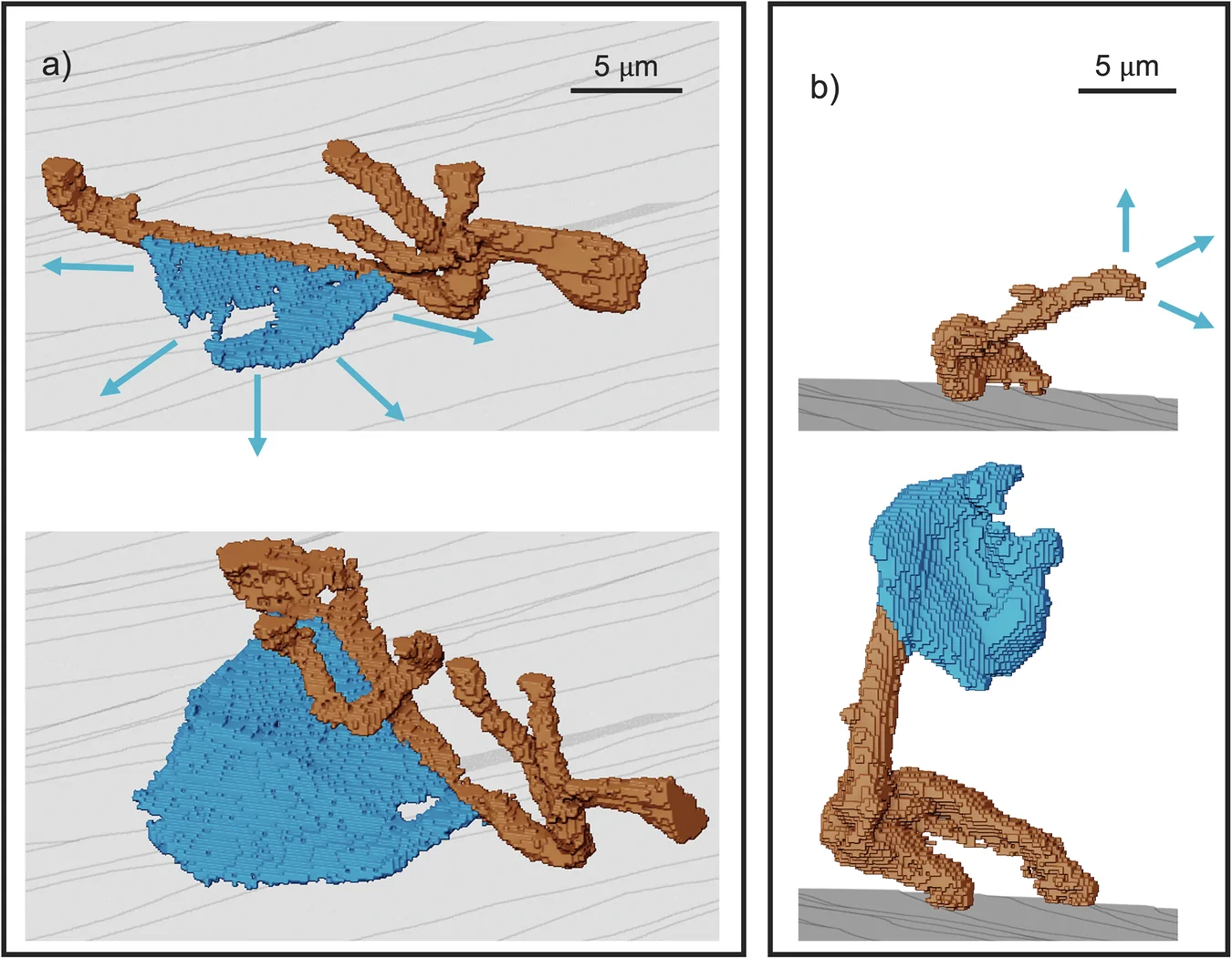
Dynamic transitions between 1D needle-type and 2D sheet-type growth modes. **a** Sheet growing from the side of a needle between 24 and 60 min of deposition (1 mA/cm^2^). **b** Sheet growing from tip of a needle between 6 and 12 min of deposition (1 mA/cm^2^). Sheets are shown in blue, needles in brown and the bulk Li electrode in gray. Blue arrows indicate growth direction of the 2D sheets.

The detailed analysis of individual deposits also highlights transitions between different dimensionalities, e.g., from 1D (needle-type) to 2D (sheet-type) segments during deposition. For instance, Figs. 7a and S7 show sheets growing from the side of a needle, expanding in-plane. Similarly, Fig. 7b shows a sheet formed at the tip of a needle. The converse transition of growth mode, from sheet to needle, can also be observed (see Fig. S19), however no instances of sheets growing directly from the bulk electrode could be found. The range of local current densities estimated for growing sheets and needles show significant overlap (Fig. S20 and Supplementary Note 7), suggesting that local current density during the growth phase does not determine the morphology. Instead, the initial growth of new grains/branches could dictate which morphology evolves during their subsequent growth. To trigger different types of growth, changes/cracks in the SEI-layer could impact the nucleation of new growth sites. This electrolyte forms a roughly 10 nm thick, amorphous SEI^42^, containing several inorganic components, such as LiF, Li_2_S, Li_2_O and Li_3_N, as determined by X-ray photoelectron spectroscopy experiments (Fig. S21 and Supplementary Note 8). This type of interphase can be expected to be brittle and thereby prone to crack due to stress buildup during growth^43^.

**Fig. 8:**
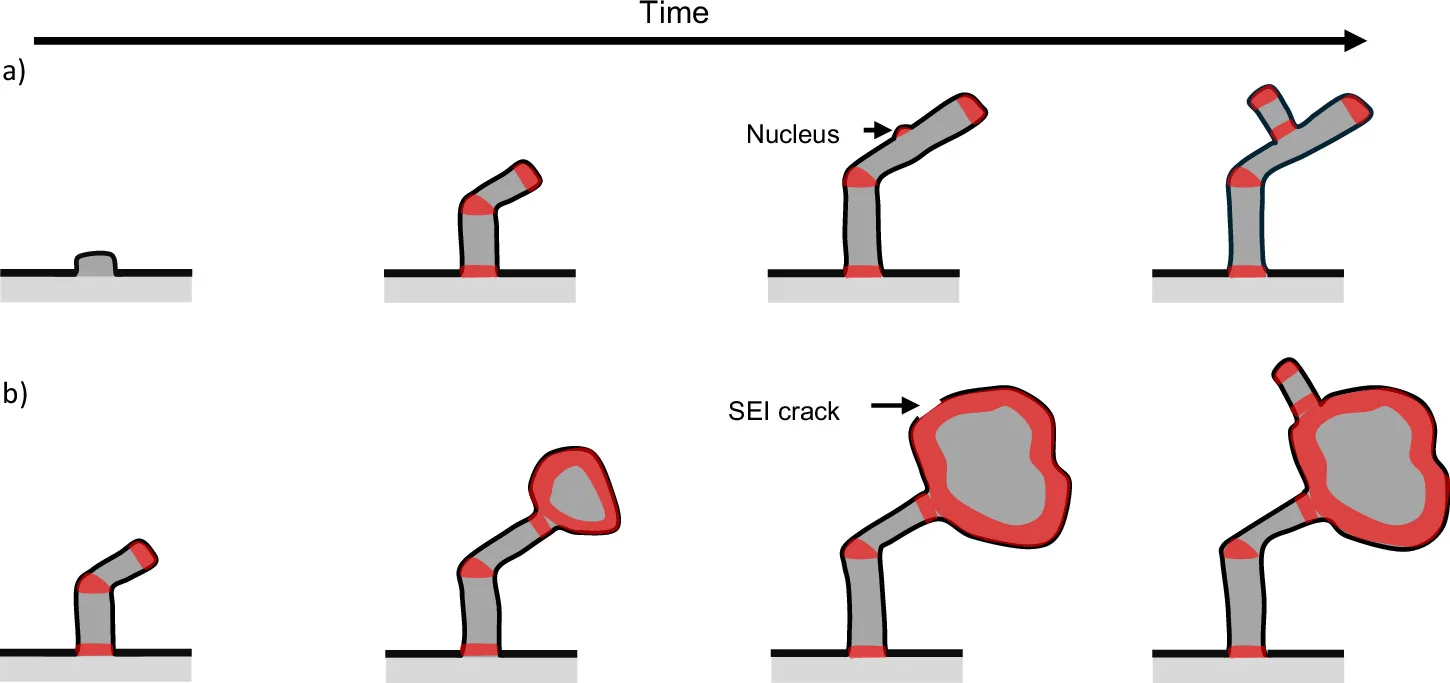
Schematic of lithium growth modes and proposed transition mechanisms. **a** Growth of a needle; **b** Growth of a sheet from a needle. Deposited Li is shown in dark gray, with semi-transparent red marking growth zones in each timestep, thin black lines representing the SEI and thicker black lines the native layer covering the bulk electrode (light gray).

Figure 8 summarizes the observed growth modes. After a deposit has pierced the native surface layer, preferential growth of this deposit is observed. Needles are shown to grow through elongation, which occurs in parallel in several segments. This can be rationalized as crystal grains growing along specific directions, forming growth zones at the needle base, tip or at kinks. New branches have been observed to grow from the sides of existing needles (Figs. S7, 8a and Supplementary Note 9), which could be rationalized by a long diffusion path to the growth zones. Segments with sheet-like morphology are also observed (Fig. 8b) to grow from needles during the deposition. While the mechanism for their formation remains to be determined, they grow from the edges through in-plane expansion. As the sheet grows, stress will be exerted on the SEI-layer which can crack, exposing fresh Li, which could trigger growth of a needle from the crack.

In summary, we present time-resolved analysis of lithium metal electrodeposition in 3D with a sub-micron spatial resolution, highlighting the potential of in situ X-ray nano-tomography to elucidate the growth modes and formation mechanisms of Li structures. Lithium electrodeposition is captured at multiple scales, ranging from entangled lithium structures, down to (sub)micron-sized segments within individual lithium deposits. A large field of view (102 × 102 × 102 µm^3^) allowed the evolution of lithium structures to be tracked over a longer time (1 h of deposition) as the growing structures stay within the field of view. Two fundamentally different Li structures coexist and build up the mossy Li morphology, (i) one-dimensional needle-like lithium, presumed to be single crystalline and (ii) two-dimensional wrinkled, amorphous or polycrystalline, sheets. Based on our data, the evolution of these structures cannot be explained by ion diffusion limitations in the electrolyte, instead we propose a model for Li growth, where Li structures grow with almost constant thickness, through branching, elongation of existing branches and planar growth of sheets. In a single connected structure, different segments grow simultaneously, however freshly deposited parts grow faster, pointing to a gradually increasing resistance of the SEI over time. Transitions between sheet and needle morphologies can occur within one connected structure, and it is hypothesized that transitions are caused by changes/cracks in the SEI-layer impacting the initial growth of new lithium grains, but more work is required to fully understand the mechanism. For instance, the coupling between SEI layer properties and deposit morphology needs to be rationalized, calling for both systematic studies exploring the impact of current density and electrolyte formulation, to shed light on the role of the SEI by tuning its characteristics, as well as theoretical modeling based on 4D tomography datasets. Development of more advanced computational models, which account for, e.g., the SEI layer properties and lithium crystal anisotropy, will be important to help explain how different lithium morphologies form. More broadly, in situ X-ray nano-tomography experiments could help explore how different processes and parameters, such as stripping or applied stack pressure, influence the morphology of lithium metal electrodes.

## Methods

### Electrolyte preparation

Lithium bis(fluorosulfonyl)imide (LiFSI, Solvionic 99.9%) salt was dried at 80 °C for 72 h in a Büchi oven inside an argon-purged glovebox. 1,2-Dimethoxyethane (DME, Sigma–Aldrich 99.5%) was dried over molecular sieves (3 Å) for several days and filtered through a polytetrafluoroethylene syringe filter. The final electrolyte was a 4 M mixture, magnetically stirred at room temperature for 24 h to ensure complete salt dissolution. Note that the high salt concentration helps improve the image contrast (between Li metal and electrolyte) in this experiment.

### Tomography cell and cycling

In situ X-ray nano-tomography measurements were carried out in a customized electrochemical polyether ether ketone (PEEK) cell with 1.59 mm diameter stainless steel rods acting as the current collectors (Fig. S1a, b). A Li metal symmetric cell was assembled with electrode separation of around 1 mm. Electrodes were prepared by pushing the current collector rods through a pristine Li foil, 200 µm thick. To ensure proper sealing of the cell, ultraviolet-curing glue was applied to the points where potential leaks could occur.

Li deposition was performed with an OrigaFlex OGF500 potentiostat/galvanostat using a dedicated setup developed at the ID16B beamline (23±0.05 °C)^44^. When tomographic acquisitions were performed, the cell was rested at OCV in order to assess possible beam interactions. The current density of 1 mA/cm^2^ was calculated based on the top area of the metal pins where the Li-electrodes were attached. Since the characteristic growth modes of needle and sheet were confirmed to arise also when lithium was plated without interruption (Fig. S5, SEM images), the morphology should not have been qualitatively altered by the interruptions, and ion depletion should not occur at the electrode with or without pauses (Supplementary Note 4). All tomography data reported except Fig. S6 were measured on the same cell.

### Nano-tomography

Measurements were carried out at the ID16B beamline at the European synchrotron radiation (ESRF) in Grenoble, France^45^. To mitigate beam damage, the experiment was carried out with a reduced beam flux of 3.4 × 10^11^ ph/s, using 3.5 mm of Si filters and diminished aperture opening of a horizontal slit. The sample was illuminated with a coherent conical pink beam^46^ of energy 29.45 keV (ΔE/E ~ 10^−2^), produced by focusing the beam upstream of the sample (50 × 50 nm) using Kirkpatrick–Baez (KB) mirrors. Projections were acquired using a PCO Edge 4.2 complementary metal–oxide–semiconductor (CMOS) camera (2048×2048 pixels), equipped with a 30 µm lutetium oxyorthosilicate (LSO) scintillator. For a single tomogram, 2505 projections were taken with 50 ms integration time for each projection. For holo-tomography, four different sample-detector distances are used resulting in a total beam exposure time of 501 s for one measurement. Using these settings, no bubble formation, indicative of beam-induced electrolysis, was observed during the experiment (see Fig. S22). Further, voltage perturbations during beam exposure can be a sign of beam damage^44^, but here no voltage changes could be observed during tomography scans (Fig. S23) apart from the expected relaxation of the concentration overpotential. Complementary ex situ SEM measurements (Fig. S5) also showed good agreement with the lithium morphologies observed during the tomography experiment, suggesting that any influence of the X-ray beam on the resulting morphology is minor.

Overview measurements (526 × 526 × 526 µm^3^) were carried out with a 257 nm voxel size using a single sample-detector distance, giving a total measurement time of around 2 min. A two-step reconstruction process was used for this data, described also in reference ^47^. First, the software PyNX^48^ was used to perform iterative phase retrieval calculations (a Paganin-like approach with a complex refraction index ratio δ/β = 120 was used as the starting point). In the second step, a 3D volume was reconstructed from the acquired 2D phase maps using filtered back-projection, implemented in Nabu (https://gitlab.esrf.fr/tomotools/nabu).

The high-resolution X-ray nano-tomography measurements had a voxel size of 50 nm and were carried out using the holo-tomography approach, where tomograms were taken at 4 different sample-detector distances and combined into a single measurement during the phase retrieval process (Fig. S1c)^49^. Adding time to let the sample equilibrate after moving it (45 s per distance), the total time for a holo-tomography measurement was around 12 min. The phase information was extracted from the raw data projections using a Paganin-like phase retrieval recursive algorithm with a complex refraction index ratio δ/β = 563^49,50^. Then the final volume (102 × 102 × 102 µm^3^) was reconstructed using a filtered back-projection algorithm in the software PyHST2^51^. Post-processing ring removal and 16-bit conversion was performed using a dedicated MATLAB script^52^.

The center of the electrode was imaged during the experiment and the representativity of the (102 × 102 × 102 µm^3^) subregion tracked with high resolution X-ray nano-holo-tomography measurements was confirmed by comparing the lithium morphology there to surrounding locations on the electrode (Fig. S24). Qualitatively, these show similar morphologies.

### Image processing and analysis

The reconstruction of a phase contrast tomogram renders a 3D representation of the scanned sample, where each voxel has a grayscale value corresponding to the electron density of that material. To limit computational cost and decrease the noise content, the data were binned to a 100 nm voxel size before the segmentation for all figures except Fig. 4, where the analysis was performed without binning of data. To enable visualization and quantitative analysis of the formed lithium structures, each voxel was labeled as bulk lithium, deposited lithium or electrolyte. First, the bulk Li electrode was manually segmented. After masking the bulk electrode, the remaining voxels were classified as deposited lithium or electrolyte using the software Illastik^53^. A machine-learning based approach was applied, where regions of electrolyte and lithium metal structures were interactively selected. Three unique random forest-based classifiers were trained for each different voxel size. One for the overview measurements (514 nm, binned), one for the full holo-tomography (100 nm, binned) and one for the holo-tomography sub-region shown in Fig. 6 (50 nm, no binning).

The areal capacity of deposited lithium was calculated by projecting segmented structures onto the electrode, generating 2D images where pixel values correspond to the number of Li-containing voxels, N, above that point on the electrode. The equivalent areal capacity of lithium projected onto each point on the electrode, ${Q}_{{{{\rm{A}}}}}$, was calculated as

$${Q}_{{{{\rm{A}}}}}=\frac{{nF}\rho {V}_{{{{\rm{vox}}}}}N}{{A}_{{{{\rm{vox}}}}}{M}_{{{{\rm{w}}}}}}$$

where n is the number of electrons transferred (1), F is Faraday’s constant, ρ the density of lithium, ${V}_{{{{\rm{vox}}}}}$ is the volume of a voxel, and ${M}_{{{{\rm{w}}}}}$ the molecular weight of lithium atoms.

The local thickness, defined for each voxel inside a segmented lithium structure as the diameter of the largest sphere that fits inside the lithium structure and contains that voxel, was determined using built-in functions in ImageJ^54,55^.

Connectivity analysis was carried out on volumes containing only the electrodeposited lithium (removing the bulk electrode) (Fig. S25a). The analysis was carried out in Avizo, assigning the same label to neighboring lithium voxels, and visualizing different labels in different colors (Fig. S25b, removing any deposit smaller than 1.25 µm^3^). Voxels sharing a face were considered neighbors (Fig. S25c shows the voxels sharing a face with the central voxel).

### Scanning electron microscopy

0.2 mAh/cm^2^ Li was galvanostatically deposited at 1 mA/cm^2^ in a Li-Li symmetric cell. The cell was a custom-built PEEK T-Cell with Swagelok type sealing where a 1 mm thick polypropylene spacer separated the electrodes without applying any external pressure to the Li-metal electrodes. Li electrodes (Chemetall, 200 µm thick, 5 mm diameter) were punched from pristine foil and the cell was filled with 75 µL of electrolyte.

The cell was cycled inside an argon-purged glovebox (<1 ppm O_2_, <0.01 ppm H_2_O, 22±1 °C) after which the electrode extracted, soaked in DME for 1 min, and then rinsed once more to remove any loose Li pieces. Under an argon atmosphere, a dedicated transfer vessel moved the electrodes from the glovebox to the instrument. SEM images were obtained using a PHI 700 scanning Auger Nanoprobe, a 10 kV acceleration voltage, and a 5 nA beam current.

### Electrochemical impedance spectroscopy

A three-electrode Li-Li cell with a Li-metal reference electrode was assembled in the same type of PEEK T-cell used for SEM experiments inside an argon-purged glovebox (<1 ppm O_2_, <0.01 ppm H_2_O, 22±1 °C). EIS spectra were acquired on a biologic VMP-3 potentiostat in the frequency range 100 kHz − 100 mHz (8 points per decade), with a potentiostatic modulation of 10 mV. EIS spectra for the Li working electrode were collected at OCV before the experiment, and at OCV directly after interrupting galvanostatic deposition (1 mA/cm^2^) at 6, 12, 24 60 min, as well as every hour during a subsequent 6 h rest. To mimic conditions in the tomography experiment, any time the deposition was interrupted, a 30 min rest was applied before deposition continued. All EIS spectra were fitted with an *R*_1_(*R*_2_*P*_2_)(*R*_3_*P*_3_) equivalent circuit model, where *R* and *P* denote resistances and constant phase elements, with the high- and low-frequency arcs labeled as *R*_2_ and *R*_3_, respectively. Spectra were fitted in the ZView 4.0 h (Scribner) software, the fitted parameters and their relative errors are provided in Table S2.

### X-ray photoelectron spectroscopy

Li was deposited on a Cu foil (13 mm diameter) for 1 h at 1 mA/cm^2^ in a Li-Cu coin cell (Wellcos, stainless steel), containing a Wellcos 2400 separator and a 10 mm diameter Li counter electrode (Honjo metal, 200 µm thick) and 40 µL of electrolyte. To remove electrolyte salt residue from these samples they were soaked in DME for 5 min and then rinsed with 100 µL of DME. Cell cycling and electrode extraction was done inside an argon purged glovebox ( < 1 ppm O_2_, <0.01 ppm H_2_O, 22±1 °C) from which samples were transported to the instrument using a dedicated transfer vessel to avoid air exposure. Measurements were done in a PHI5000 Versa Probe III spectrometer from ULVAC-PHI with monochromatic Al Kα X-rays (1486.6 eV). Energy calibration of the spectra was carried out by shifting the LiF peak to 684.8 eV.

### Electrochemical simulations

To study the link between the lithium morphology and the current distribution at the associated reactive interfaces, an electrochemical model was implemented. The simulations were conducted using a home-made code leveraging the open-source TRUST high performance computing software (https://cea-trust-platform.github.io/). Similar approaches have been recently reported using the commercial software BatteryDict^56,57^. The segmented microstructure obtained from the X-ray nano-holo-tomography at the 24 min timestep was used as a base mesh to define the geometrical regions for the simulation (local phase volume fraction and interfacial surface). The model focused on simulating transport phenomena in the electrolyte domain by solving the potential and Li-ion concentration as well as the Butler – Volmer current at the interface between the electrolyte and the Li metal (dendrites and the electrode native surface)^58–61^. It provides the local instantaneous plating current on the Li-metal surface but does not predict the evolution of the surface shape.

## Supplementary information

Supplementary Information

Transparent Peer Review file

## Source data

Source Data

## Data Availability

The synchrotron data generated in this study is available from the ESRF at https://doi.org/10.15151/ESRF-ES-1259505667. The segmented 3D data have been deposited in the Zenodo repository, https://doi.org/10.5281/zenodo.21820984. All other relevant data that support the findings of this study can be obtained from the corresponding authors upon request. Source data are provided with this paper.
