# Supplementary material for: Exploring Li growth modes in Li metal electrodes using X-ray nano-tomography
Source: Nat Commun. 2026 Sep 29;17:11233. doi: 10.1038/s41467-026-78182-9 (PMC13720151; doi:10.1038/s41467-026-78182-9)
Supplement: Supplementary file 1 [file 41467_2026_78182_MOESM1_ESM.pdf]

SUPPLEMENTARY INFORMATION

Exploring Li growth modes in Li metal electrodes using X-ray nano-tomography

Josef Rizell[1], Victor Vanpeene[2]*, Martina Olsson[1], Jan Jamroz[1,3,4], François Cadiou[5], Hervé Manzanarez[5], Valentin Vinci[6], Olga Stamati[6,7], Julie Villanova[6], Sandrine Lyonnard[2], Aleksandar Matic[1,4]*

[1] Department of Physics and Astronomy, Chalmers University of Technology, Origovägen 6B 412 58 Gothenburg, Sweden
[2] Univ. Grenoble Alpes, CEA, CNRS, Grenoble INP, IRIG, SyMMES, 38000 Grenoble, France
[3] Faculty of Physics, Warsaw University of Technology, Koszykowa 75, 00-662 Warsaw, Poland
[4] Wallenberg Initiative Materials Science for Sustainability (WISE), Department of Physics and Astronomy, Chalmers University of Technology, SE 412 96 Göteborg, Sweden
[5] Univ. Grenoble Alpes, CEA, Liten, DEHT, 38000, Grenoble, France
[6] The European Synchrotron, 71, avenue des Martyrs, CS 40220, 38043 Grenoble Cedex 9, France
[7] Univ. Grenoble Alpes, CNRS, Grenoble INP, 3SR, 38000 Grenoble, France

* Corresponding authors: matic@chalmers.se, victor.vanpeene@cea.fr

Supplementary Note 1: Phase-contrast holo-tomography

Li metal and the surrounding electrolyte are both weakly attenuating materials, resulting in limited contrast using conventional X-ray absorption tomography.[1] With nano-holo-tomography, materials are differentiated based on the X-ray phase shift, which is directly proportional to their electron density[2]. The phase shift is retrieved from the recorded interference patterns of the transmitted X-ray beam through the sample.[3] During a measurement, the cell (Fig. S1a,b) is rotated (360°) while the image of the transmitted X-ray beam is recorded on a 2D camera (Fig. S1c). To compensate for the zero-crossing effect while retrieving some spatial frequencies, the interference patterns of four different propagation distances are combined.[3] The improvement in image quality using phase contrast compared to absorption contrast is attributed to the refractive indices of electrolyte and lithium metal (Fig. S2). The difference in the real part of the refractive index, relating to the phase contrast, is around 3 orders of magnitude larger than the difference in the imaginary part of the refractive index, corresponding to the absorption. Thus, recovering phase information significantly helps to improve the contrast between lithium and electrolyte.

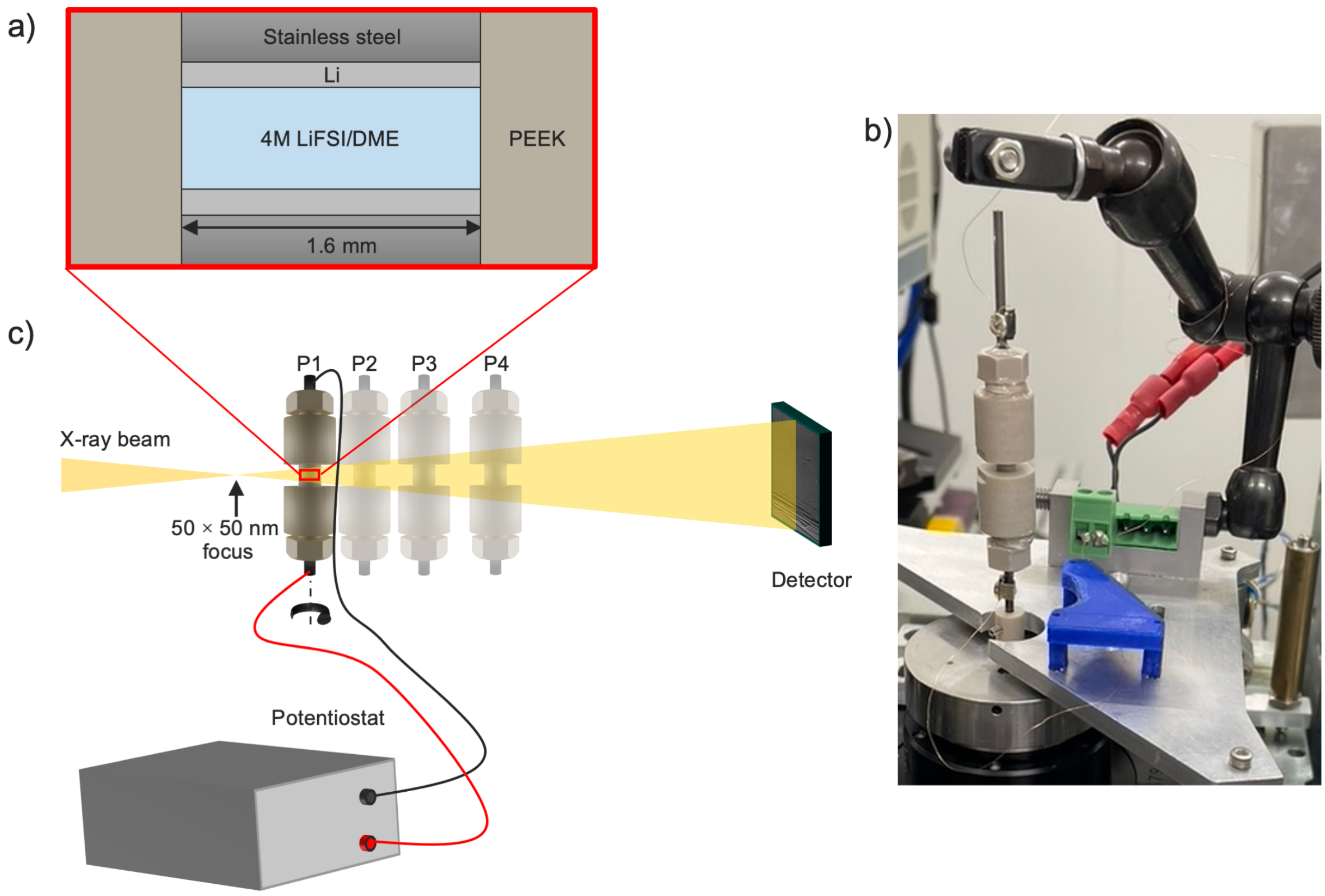

Fig. S1. *In situ* nano-holo-tomography setup. **a)** Sketch of the electrochemical cell used. **b)** Image of the cell, connected to the potentiostat, at the instrument. **c)** Holo-tomography acquisition. The black arrow illustrates the rotation of the cell and semi-transparent cells demonstrate the use of different sample-detector distances (P1-P4) for the holo-tomography technique.

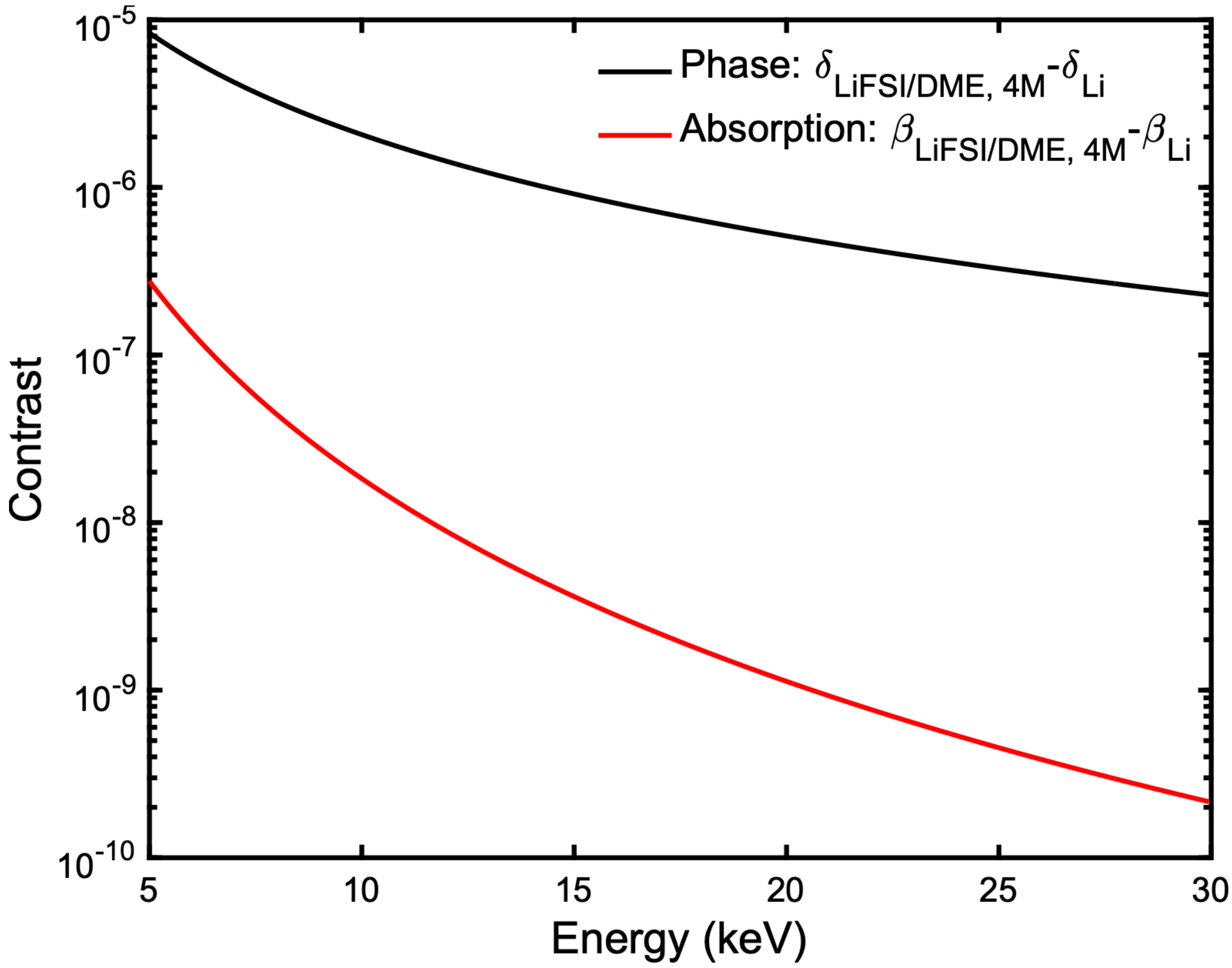

Fig. S2. Difference in real (δ) and imaginary (β) part of the refractive index between the electrolyte and Li metal.

Supplementary Note 2: Image resolution

Projections are acquired at a set voxel size that defines the size of the reconstructed volume. For high resolution experiments a voxel size of 50 nm was used. The spatial resolution of the images used for quantification depends on several parameters including source size, acquisition parameters determining the noise level in the raw data, mechanical stability of the cell and the growing structures, and the reconstruction parameters.

Here, the to estimate the image resolution, sharp profiles in the reconstructed tomogram were identified (Fig. S3a). For each region, the grayscale intensity along a line profile perpendicular to the sample surface was extracted and used to calculate the modulation transfer function (MTF, Fig. S3b).[4] The spatial resolution, x_{res}, was determined using the spatial frequency f_{res} at which MTF dropped to 10% of its maximum, according to

$$x_{\mathrm{res}} = \frac{2\pi}{f_{\mathrm{res}}}.$$

The average value for the f_{res} in Fig. S3 is 2.107 voxels^{-1} (excluding the two extreme values), resulting in a true spatial resolution of 3.0 times the voxel size or 150 nm.

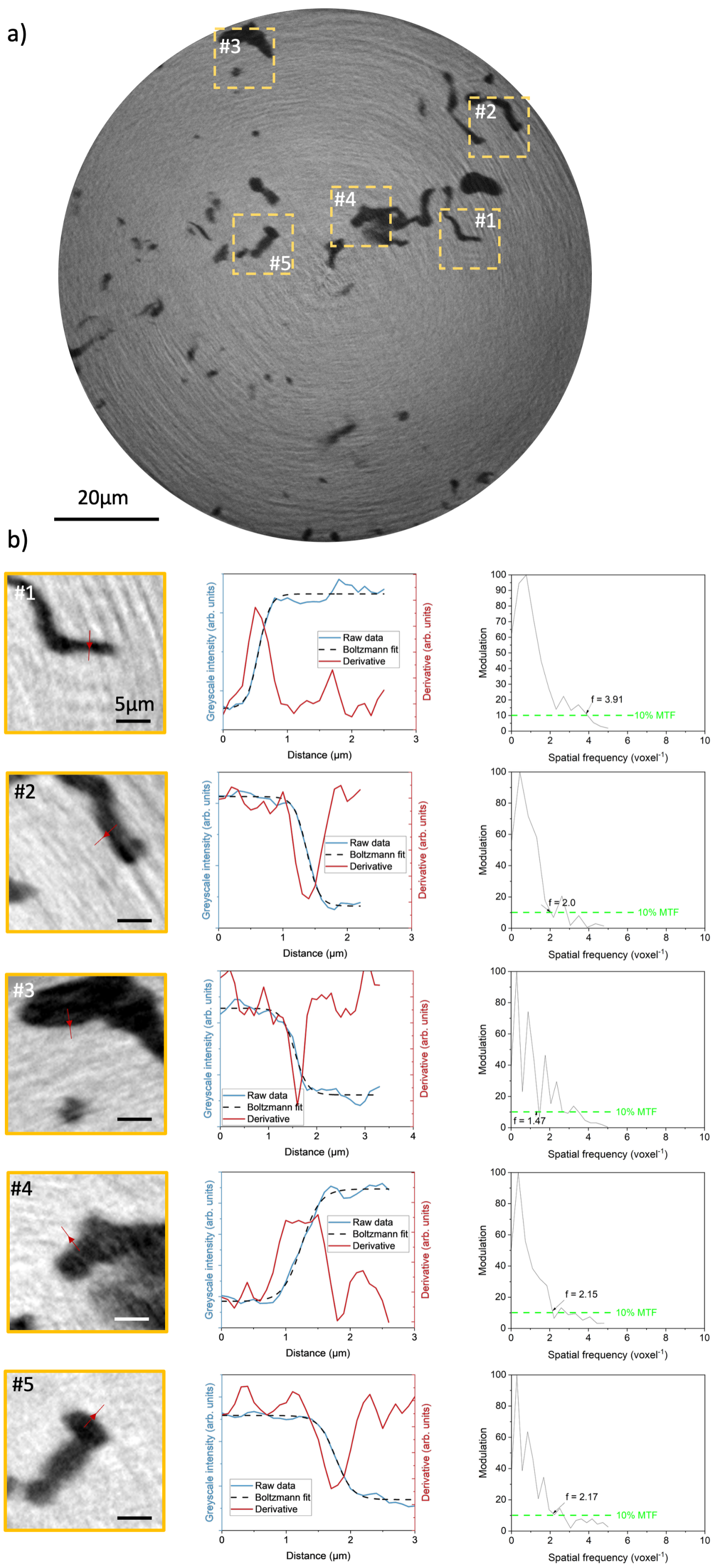

Fig. S3. a) Holo-tomography reconstructed grayscale slice at 50 nm voxel size with different Li structures used for determining the resolution. **b)** Magnified view of structures with interfaces selected for resolution estimation (red arrow) and corresponding plots of grayscale values as a function of the position along the interfacial lines and the calculated MTF, with dashed green lines marking where the MTF falls to 10% of its maximum value.

Supplementary Note 3: Cell configuration

The tomography cell operates with no applied stack pressure, and an electrode separation around 1 mm. This ensures that cells do not short circuit while keeping electrode separation small enough to avoid ion depletion at the electrode surface (see Supplementary Note 4). Note that very large electrode separations have been observed to trigger spherical lithium growth morphologies, whereas more moderate inter-electrode spacing has been shown to result in morphologies similar to those observed in this work.[5]

Still, the absence of stack pressure is a clear difference between this cell and common lithium metal cell formats, where the pressure and type of separator have a strong influence on the morphology of the deposited lithium.[6] Generally, a more compact morphology with larger grains form under pressure.[7] However, the deposition without pressure is still important to understand for two reasons: 1) the driving forces controlling the lithium metal deposition are easier to disentangle in this more idealized scenario and 2) local differences in pressure are common in both coin and pouch cells,[8–10] which means that it is important to understand how lithium grows also in regions with little or no pressure.

Supplementary Note 4: Dendrite growth

The absence of fractal, self-similar structures (classical dendrites), can be rationalized considering the moderate current density during deposition. The diffusion limited current density, J^*, above which the ion diffusion to the electrode cannot be sustained by the electrolyte, eventually triggering fractal (dendritic) growth, can be calculated from the following formula:[11]

$$J^* = \frac{4 z_{\mathrm{Li}} c_0 F D_{\mathrm{Li}}}{L}$$

where, z_{Li} is the charge number of the Li ion ($z_{\mathrm{Li}} = 1$), c_0 the bulk salt concentration, F is Faraday's constant, D_{Li} the diffusion coefficient for Li ions in the electrolyte and L the inter-electrode separation. The diffusion coefficient $D_{\mathrm{Li}} = 2.06\ 10^{-6} \mathrm{cm}^2/\mathrm{s}$ for this electrolyte (determined using nuclear magnetic resonance spectroscopy at 20°C), together with an electrode spacing $L = 1$ mm, and 4 M Li-ion concentration gives a limiting current density $J^* = 3.2\ \mathrm{mA/cm}^2$. This value is significantly higher than the current applied in this work, explaining why classic (fractal) dendrites could not be observed.

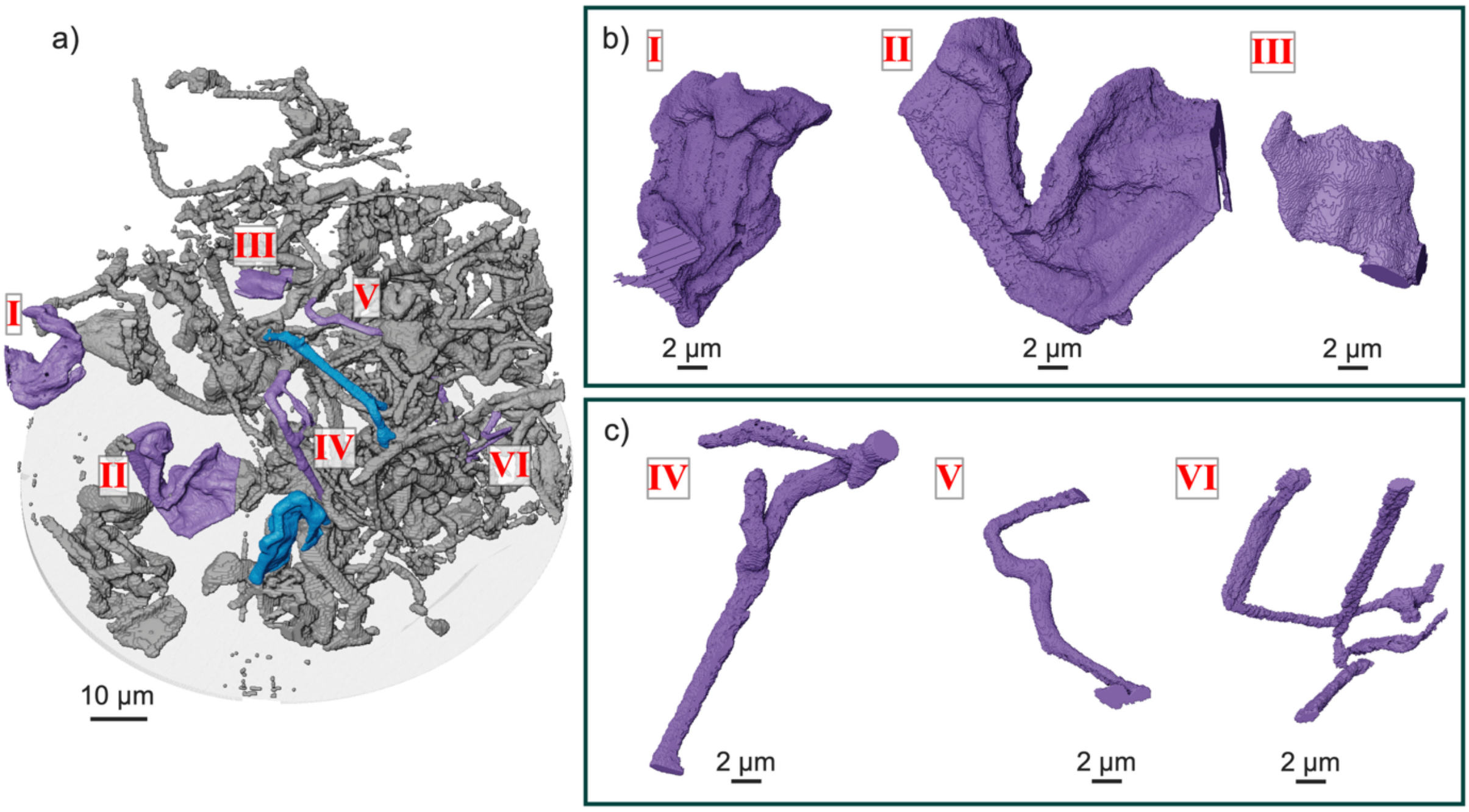

Fig. S4. Different types of lithium deposits. **a)** Volume rendering of segmented Li structures from holo-tomography scan after 60 min deposition at 1 mA/cm^2. The deposits shown in Fig. 1 are shown in blue, additional examples of lithium sheets and needles are highlighted in violet while the rest of the deposited lithium is dark gray and light gray marks the bulk electrode. **(b-c)** Examples of sheets **(b)** and needles **(c)** in an enlarged view.

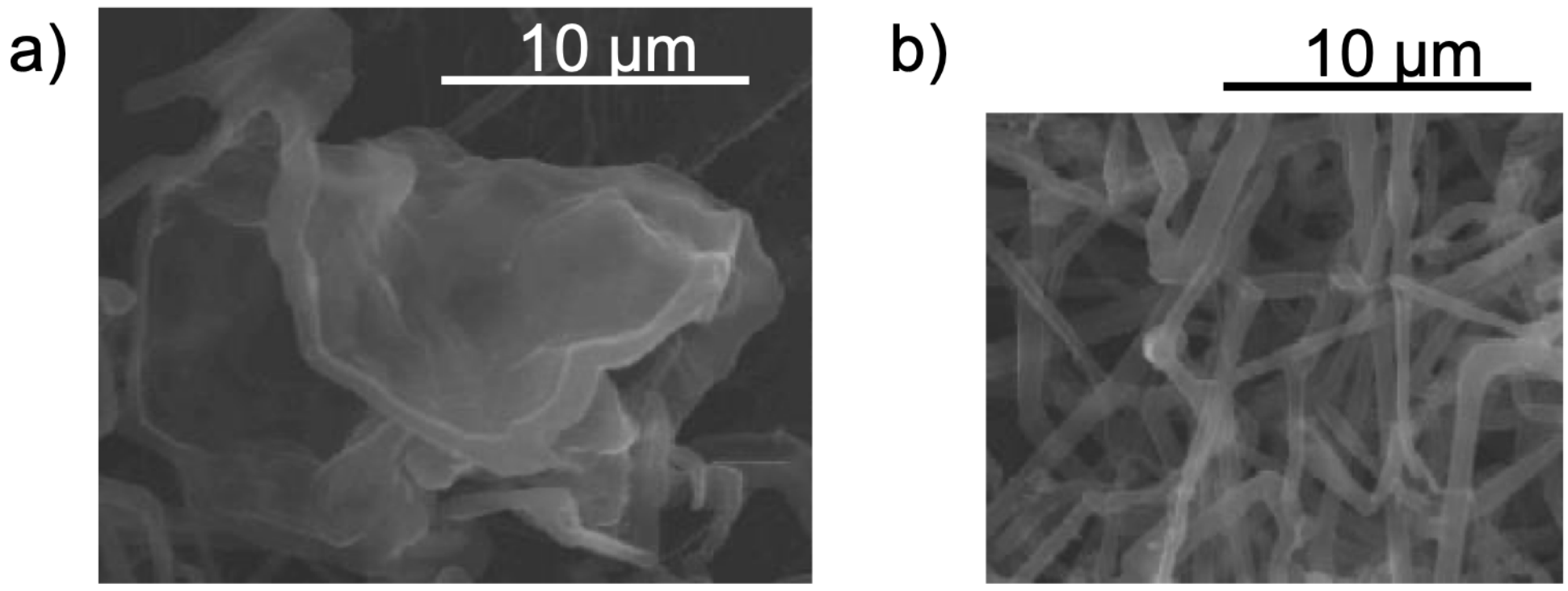

Fig. S5. *Ex situ* SEM images of a Li-metal electrode after 12 min deposition at 1 mA/cm^2 in Swagelok cells (electrode diameter 10 mm) depicting **a)** Li sheets and **b)** Li needles.

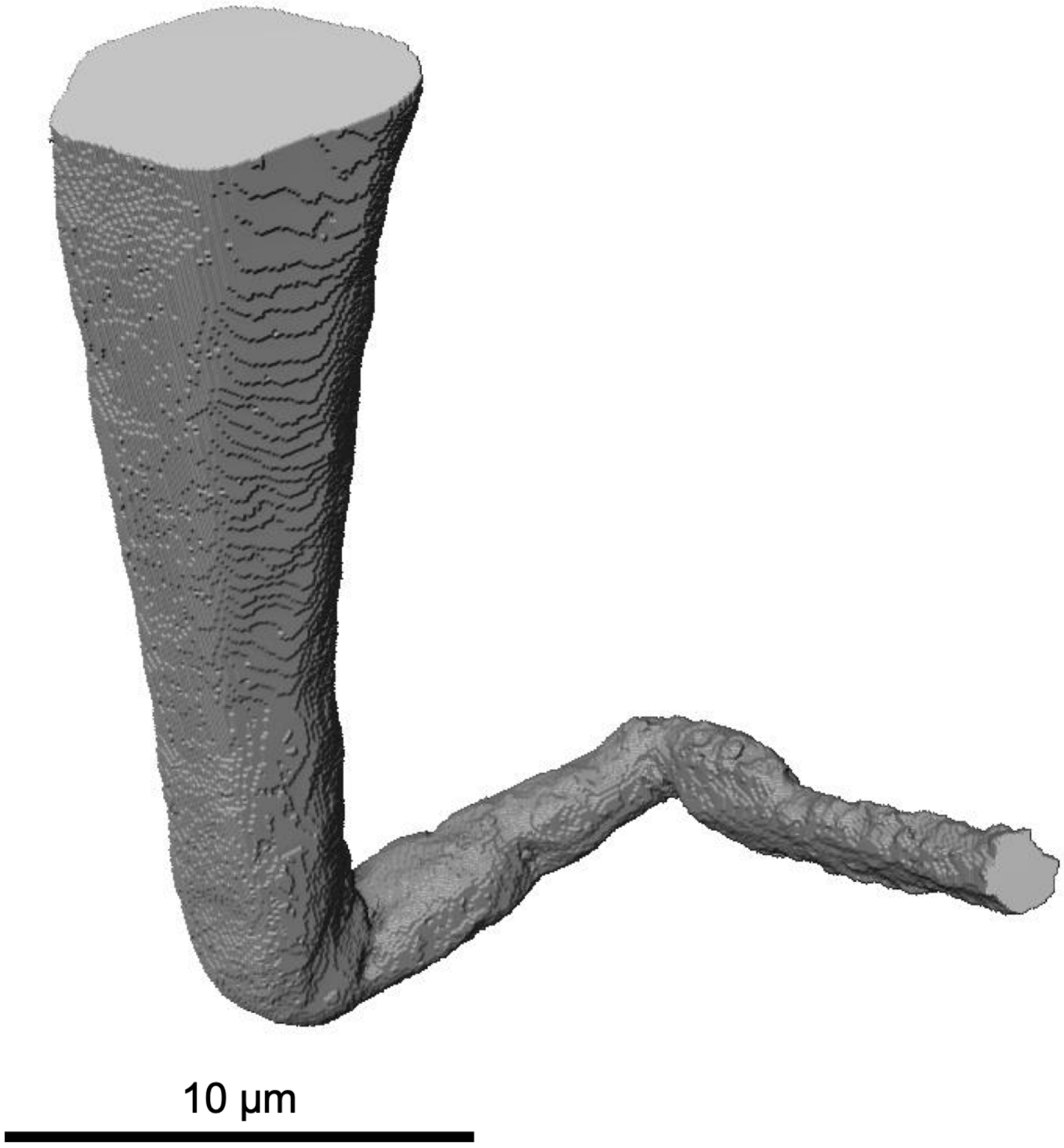

Fig. S6. Volume rendering of a segmented Li structure from a nano-holo-tomography scan after 60 min deposition at 1 mA/cm^2.

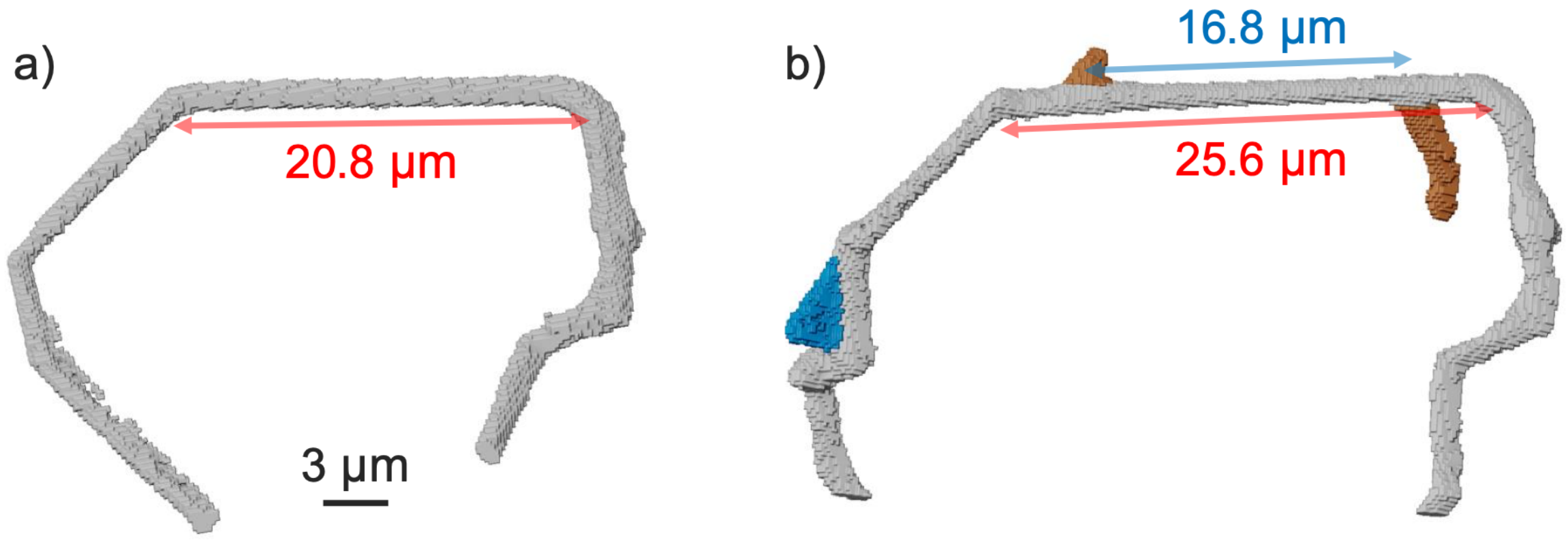

Fig. S7. Nucleation of new branches and elongation of a Li-needle. **a)** A needle found after 24 min of deposition and **b)** the same needle after 60 min of deposition at 1 mA/cm^2. New needle-like branches are shown in brown and a sheet growing from the side of a segment is shown in blue.

Supplementary Note 5: Electrolyte formulation

The 4M lithium bis(fluorosulfonyl)imide/1,2-dimethoxyethane (LiFSI/DME) electrolyte used shows excellent long term cycling performance of Li-metal cells, as shown in for instance by Qian *et al*.[12] This is in line with the general observation that highly concentrated electrolytes shown good compatibility with Li metal electrodes, increasing the coulombic efficiency and resulting in longer cycle life.[13,14]

Even though a highly concentrated electrolyte is used in this work, the results are relevant also with respect to standard electrolytes with lower salt concentration (typically 1M). Electrodeposited Li with a morphology containing sheets and needles can be observed also in these types of electrolytes and some examples are listed in Table S1.

Table S1. Reports of lithium with sheet morphology.

**Electrolyte**	**Reference**	**Figure**	**Morphology**
1M lithium hexafluorophosphate ($LiPF_6$) in ethylene carbonate (EC): ethyl methyl carbonate (EMC) 3:7 by volume	[5]	S9	Needle + Sheet
1M Lithium bis(trifluoromethanesulfonyl)imide (LiTFSI) in 1,3-dioxolane (DOL)/DME	[15]	S4c	Needle + Sheet
1M LiFSI in fluorinated 1,4-dimethoxylbutane (FDMB)	[16]	6e	Needle + Sheet
1M $LiPF_6$ in EC: dimethyl carbonate (DMC) 1:1 by volume	[17]	1e	Sheet
1M LiTFSI in EC:EMC 4:6 by weight	[18]	S9b	Needle + Sheet

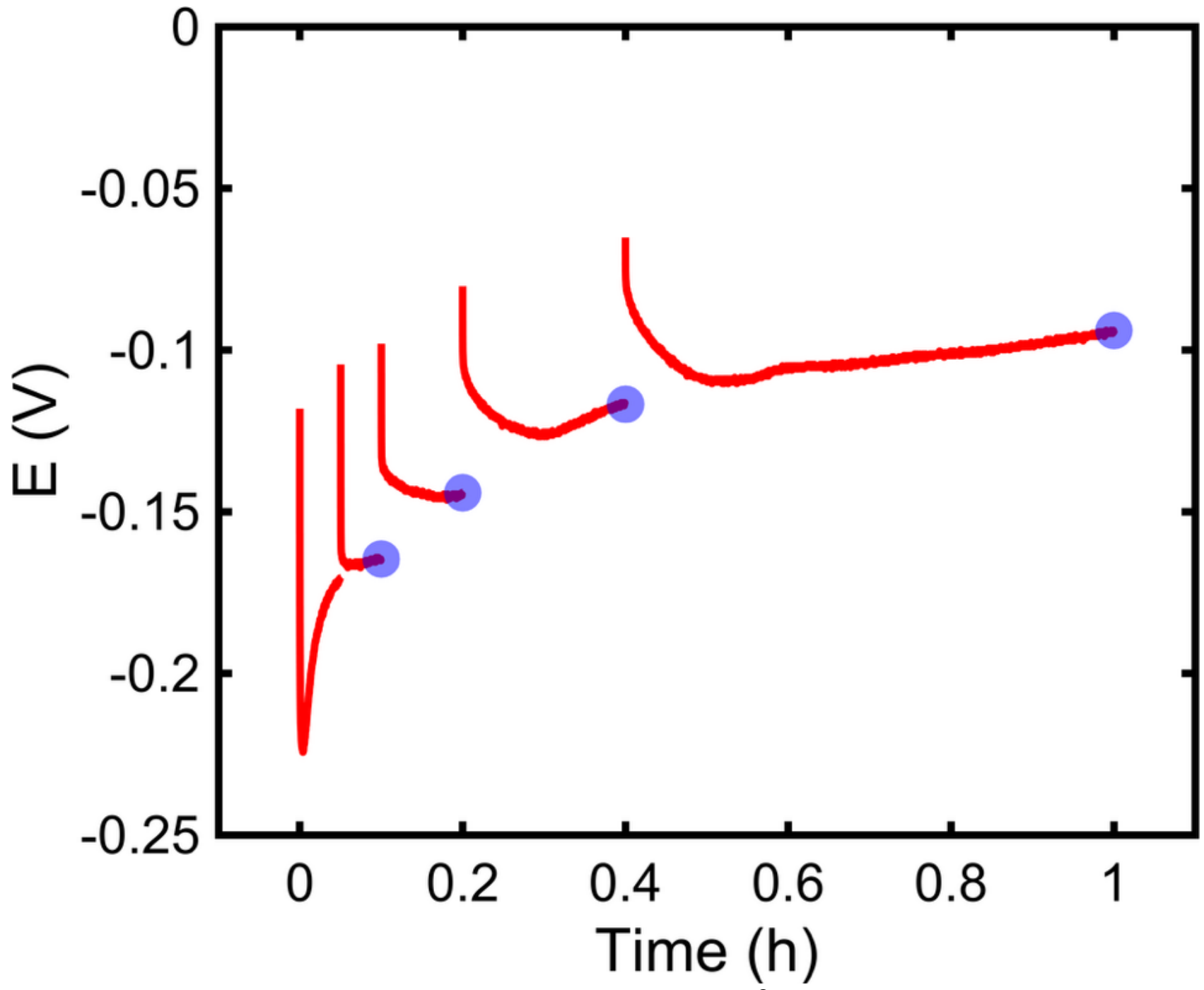

Fig. S8. Voltage profile, plating/stripping at 1 mA/cm^2. Blue rings mark where the cell was rested at open-circuit voltage to carry out holo-tomography acquisitions.

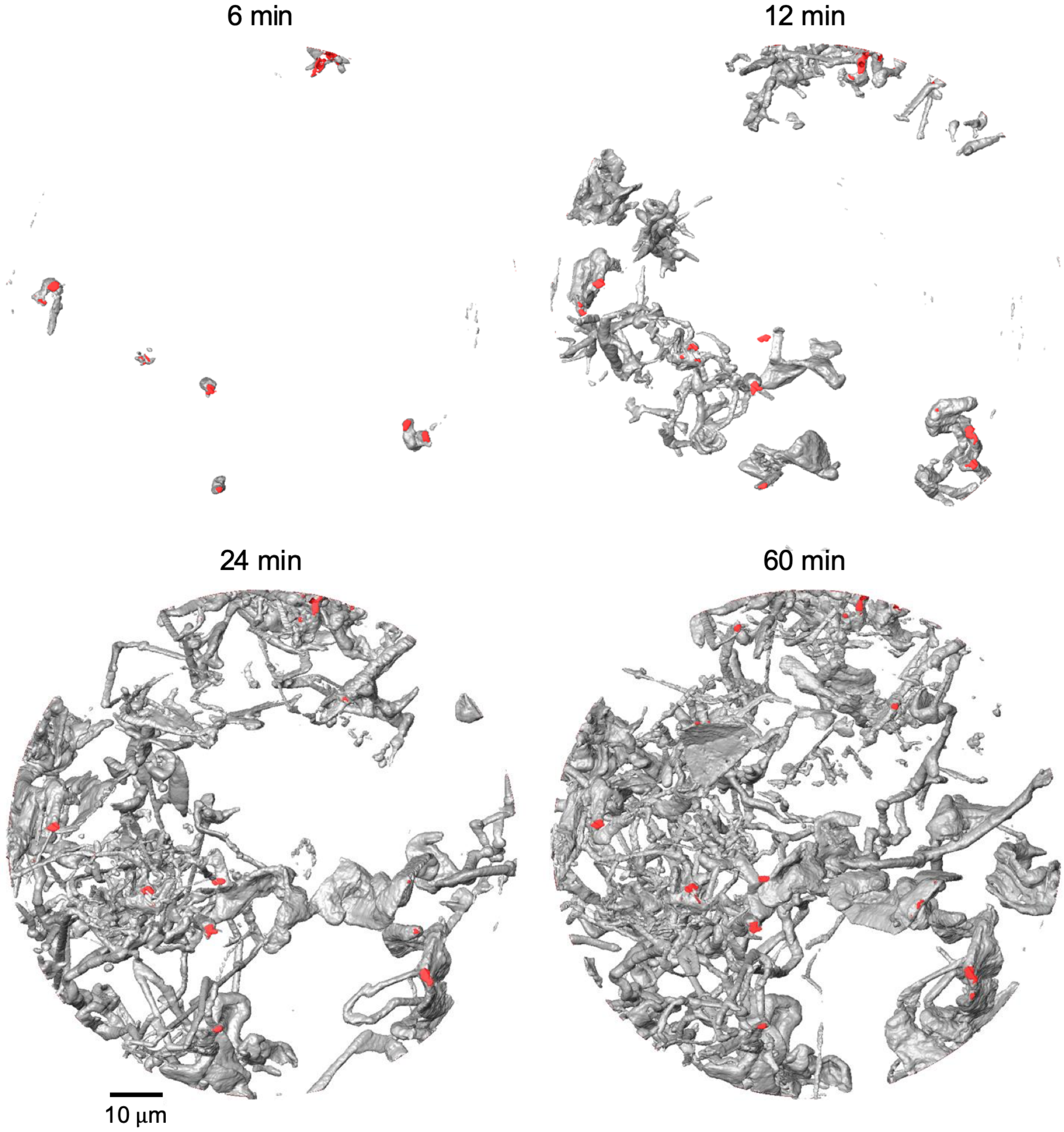

Fig. S9. Volume renderings of Li structures during deposition at 1 mA/cm^2, viewed from the bulk Li electrode side. Contact points between deposited structures (gray) and bulk electrode are marked as red.

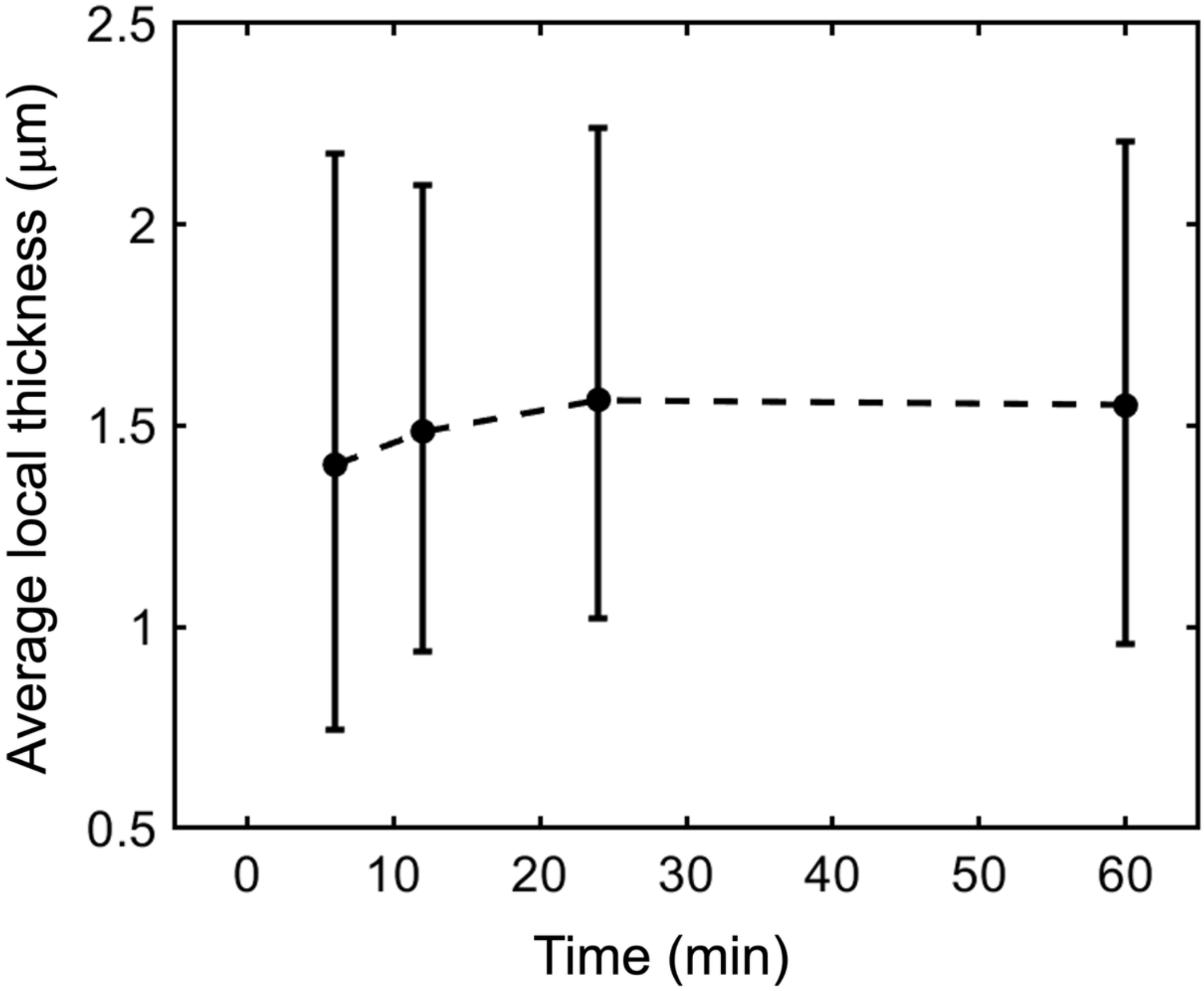

Fig. S10. Average local thickness evolution during plating at 1 mA/cm^2 (histogram in Fig. 3c) of segmented Li metal structures. Error bars represent one standard deviation from the mean, indicate the spread of the local thickness-value distribution inside a single sample. The values are calculated from between $2.2 \cdot 10^5$ (at 6 min) and $1.6 \cdot 10^7$ (at 60 min) Li voxels.

Supplementary Note 6: Electrochemical Simulations

To investigate the origin of local variations in morphology, electrochemical simulations were performed based on the actual 3D-structures obtained from tomography. The three main equations articulating the model[19–22] were:

$$-\mathrm{div}\big(\kappa_{\mathrm{el}}\mathrm{grad}(\psi)\big) - \mathrm{div}\left(-\frac{\kappa_{\mathrm{el}}\kappa_{\mathrm{add}}}{C_{\mathrm{el}}}\mathrm{grad}(C_{\mathrm{el}})\right) = 0 \quad (1)$$

$$\%_{\mathrm{v,el}}\frac{\mathrm{d}C_{\mathrm{el}}}{\mathrm{d}t} - \mathrm{div}\big(D_{\mathrm{el}}\mathrm{grad}(C_{\mathrm{el}})\big) = 0 \quad (2)$$

$$i_{\mathrm{bv}} = -i_0\left(\frac{C_{\mathrm{el}}}{C_{\mathrm{init}}}\right)^{0.5}\sinh\left(\frac{F\eta}{2RT}\right) \quad (3)$$

Equation (1) models the electronic potential in the electrolyte with κ_{el} the electrolyte conductivity in S/m, ψ the potential in V, C_{el} the Li concentration in mol/L, κ_{add} the contribution of the concentration gradient to the potential gradient. Equation (2) governs the Li concentration in the electrolyte with $\%_{\mathrm{v,el}}$ the electrolyte volume fraction, D_{el} the electrolyte diffusivity in m^2/s. Equation (3) is the Butler-Volmer exchange current at the interface electrolyte/Li metal with i_{bv} the exchange current in A/m^2, i_0 a constant factor in A/m^2, C_{init} the initial Li concentration in mol/L, F the Faraday constant in C/mol, η the opposite of the electrolyte potential in V, R the perfect gas constant in J/mol/K and T the temperature in K. These equations are solved on a 3D regular grid where the Butler-Volmer current (i_{bv}) as well as the electrolyte mass, diffusivity (D_{el}) and conductivity (κ_{el} becoming κ_{eff}) are homogenized in each mesh cell. To this end, the electrolyte volume fraction ($\%_{\mathrm{v,el}}$) and the lithium-electrolyte interface area in each cell are used to build a volumic source term (S_ψ for the potential in Equation (1) and $S_{C_{\mathrm{el}}}$ for the Li concentration in Equation (2), both in the electrolyte) localized in the cells that contain a lithium-electrolyte interface.

Integrating the segmented microstructures from nano-holo-tomography in the simulations requires a resampling of such a discrete value map into a more continuous description of each phase. The reasons for this resampling are two-fold. First, it is used to create the homogenized descriptions leveraged in the solver and second, it can be combined with a down-sampling effectively reducing the computation costs. In each subsampled cell, when both electrolyte and Li-metal are present, the contact area between lithium and electrolyte in the cell as well as the volume fraction of electrolyte are evaluated to feed the homogenized model used for the plating model in this cell.

For the simulation purposes, an artificial volumetric layer (5 cells = 2.5 µm) was built on top of the simulation volume to mimic a bulk electrolyte area in which the boundary conditions are defined (Fig. S11a). There are two boundary conditions at the top of this added electrolyte layer (opposite to the interface with the tomography volume): a Neuman type one with an imposed current flux in A/m^2 (10 A/m^2 = 1 mA/cm^2) and a Dirichlet one with an imposed Li concentration in the electrolyte (4 mol/L), both mimicking the experimental conditions.

A mesh convergence study using $3 \times 3 \times 3$ to $10 \times 10 \times 10$ resampling kernels has been carried out. The average potential in the added electrolyte layer (encapsulated by blue borders in Fig. S11a) at steady state is chosen as a representative value to compare the mesh size effect and results are shown in Fig. S11b. Fig. S11c shows the evolution of the total current at the exchange

interface (total Li flux in mol/s multiplied by the Faraday constant) with the iteration steps towards equilibrium. It shows that the kernel size does not seem to have a strong impact on the model convergence since all of the final values are within a 0.8 % relative window. Based on the similarity of the 3D fields computed from the model with different kernel sizes (Fig. S12), a $5 \times 5 \times 5$ resampling kernel is deemed to give satisfactory precision for the purpose of this study and was used to run the subsequent simulations as a compromise between accuracy and computational cost. Fig. S13 shows the gradients in the electrolyte potential computed at equilibrium over two perpendicular slices. This indicates, in conjunction with the lower Li concentration (Fig. S12 and 4a), a decrease in the Butler-Volmer current under the grown Li structure close to the bulk Li metal substrate (Fig. 4b), hence a slower Li exchange from the electrolyte to the Li metal in these areas.

Concerning the electrochemical equations within the model, the Butler-Volmer equation is typically written under the form of Equation (4) where:

- α is a charge transfer coefficient (generally α = 0.5);
- η is the overpotential $\eta = E - E_{\mathrm{eq}} = \phi_{\mathrm{S}} - \phi_{\mathrm{el}}$ for the lithium metal (open-circuit voltage equal 0 by convention), ϕ_{S} is the active material potential and ϕ_{el} the electrolyte potential;
- $i\left(C_{\mathrm{Li,el}}, C_{\mathrm{Li,S}}\right)$ is the active material current density. This term is dependent on the lithium content at the interface (1 here due to the Li metal) and on the lithium concentration in the electrolyte near the interface.

$$i_{\mathrm{bv}} = i\left(C_{\mathrm{Li,el}}, C_{\mathrm{Li,S}}\right)\left[\exp\left(\frac{\alpha F}{RT}\eta\right) - \exp\left(-\frac{\beta F}{RT}\eta\right)\right] \tag{4}$$

Making the assumption that $\alpha = 1 - \beta = 0.5$ and that $\eta = -\phi_{\mathrm{el}}$ (taking $\phi_{\mathrm{S}} = 0$ as the current collector / bulk Li metal substrate potential), Equation (4) can be simplified into Equation (3).

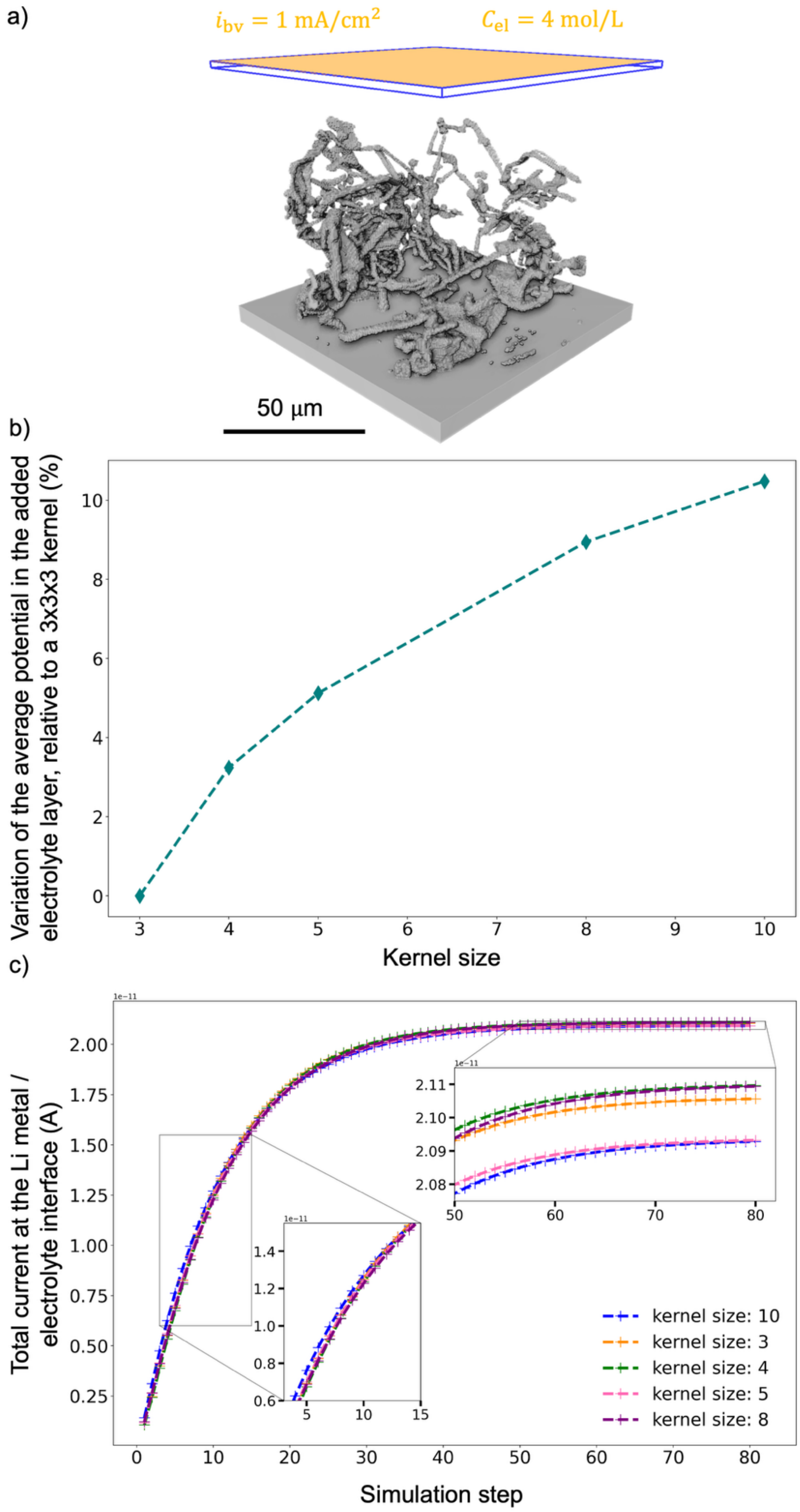

Fig. S11. Boundary conditions and influence of kernel size. a) Simulation box with Li in gray the added electrolyte layer (blue) and its border where boundary conditions are defined (orange). b) Impact of the kernel size on the computed average potential at the added electrolyte layer at equilibrium. c) Impact of the kernel size on the model convergence to equilibrium.

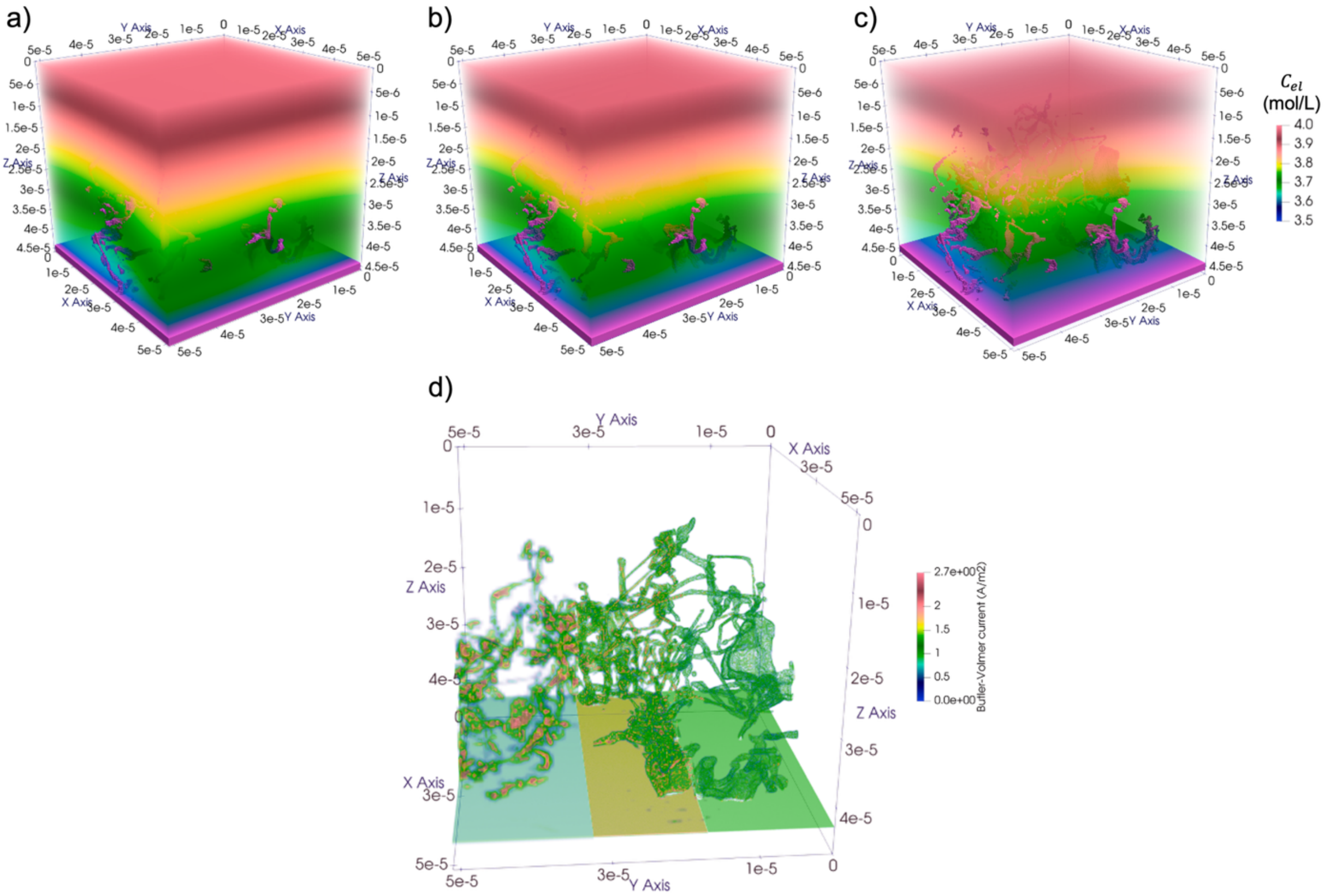

Fig. S12. Effect of the kernel size on computed fields. a) to c) Li concentration fields in the electrolyte 3D visualizations, with lithium metal shown in purple, for three different resampling kernel sizes: a) 3 × 3 × 3; b) 5 × 5 × 5 and c) 10 × 10 × 10. d) Butler-Volmer current fields in the electrolyte. 3D visualizations for three different resampling kernel sizes, 10 × 10 × 10 on the left-hand side (blue), 5 × 5 × 5 in the middle (olive) and 3 × 3 × 3 on the right-hand side (green). Deposited lithium metal is shown in green.

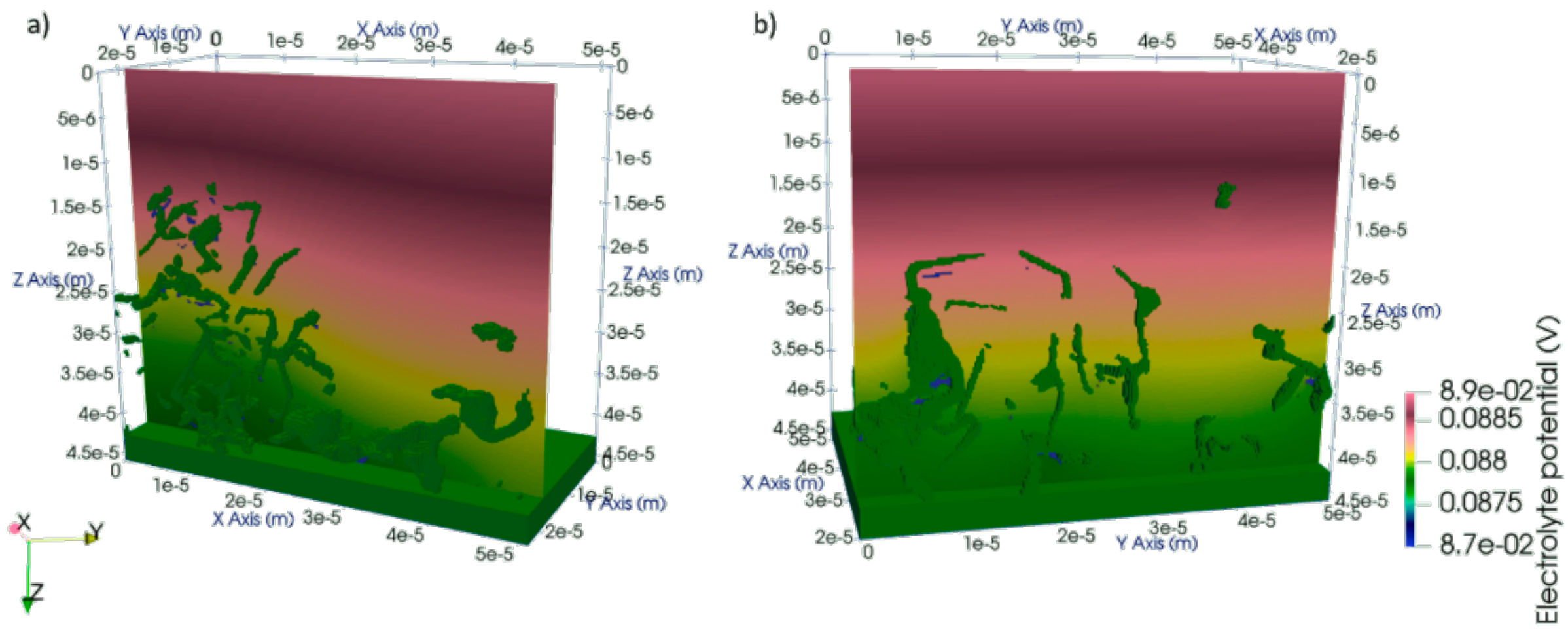

Fig. S13. Computed electrolyte potential field. 2D slices showing the electrolyte potential fields at equilibrium over the depths of the simulation volume in two perpendicular slices, a) XZ plane and b) YZ plane. The additional 3D renderings show the position of the Li structure and how they locally influence the potential gradient. The 3D Li metal structure color (green) is not linked to the potential field color bar.

The parameters κ_{add} and D_{el} (used in Equations (1) and (2)) were determined using Equations (5) and (6):

$$\kappa_{\mathrm{add}} = 2\frac{RT}{F}(1+\gamma)(1-t_{+}) \quad (5)$$

$$D_{\mathrm{el}} = 2(1+\gamma)\frac{\kappa_{\mathrm{el}}k_{\mathrm{B}}T}{1000N_{\mathrm{a}}e^{2}C_{\mathrm{el}}}(1-t_{+})t_{+} \quad (6)$$

Where γ is the electrolyte activity coefficient, κ_{el} the bulk electrolyte conductivity in S/m, t_{+} the transference number for the Li^{+} ions in the electrolyte, k_{B} the Boltzmann constant in J/K, N_{a} the Avogadro number in mol^{-1} and e the charge of one electron in C. For this study, γ was taken as 0 and the transferance number t_{+} inferred from Equation (6) given a Li^{+} diffusivity in the electrolyte of 2.062×10^{-11} m^{2}/s, measured using nuclear magnetic resonance spectroscopy at 20 °C.

The volume fraction $\%_{\mathrm{v,el}}$ and interfacial surface s_{el} maps from the tomography data were incorporated in Equations (1), (2) and (3) either directly or through the source terms S_{ψ} and $S_{C_{\mathrm{el}}}$ as well as the values κ_{eff} and $C_{\mathrm{el,0}}$ as described in Equation (7). Where C_{init} is the initial Li concentration map in the electrolyte over the simulation volume.

$$\begin{gathered} S_{\psi} = -i_{\mathrm{bv}}s_{\mathrm{el}} \\ S_{C_{\mathrm{el}}} = \frac{S_{\psi}}{1000F}(1-t_{+}) \\ \kappa_{\mathrm{eff}} = \%_{\mathrm{v,el}}\kappa_{\mathrm{el}} \\ C_{\mathrm{el,0}} = \%_{\mathrm{v,el}}C_{\mathrm{init}} \end{gathered} \quad (7)$$

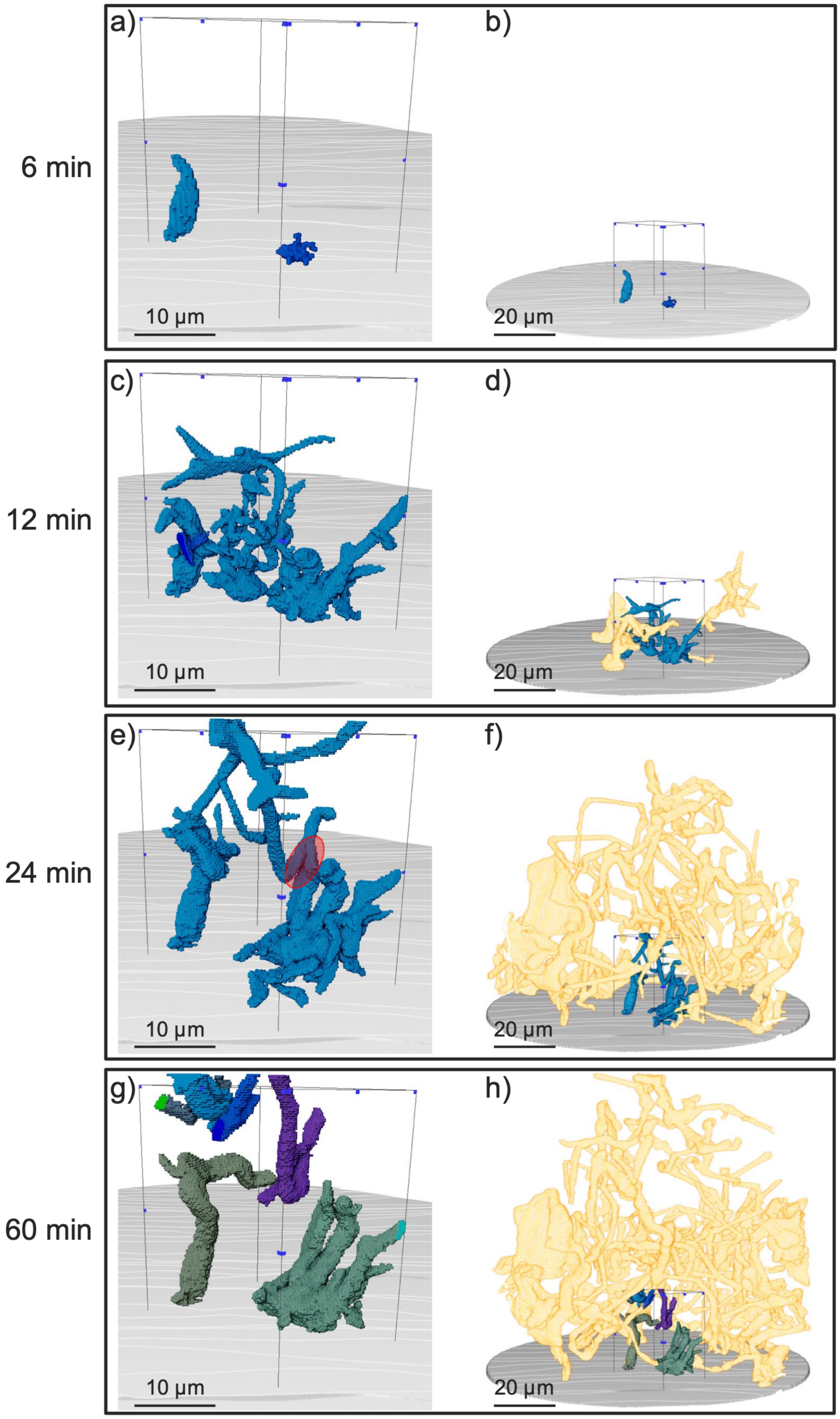

Fig. S14. Connectivity of Li deposits at different time points during deposition at 1 mA/cm^2. Volume renderings of segmented Li deposits are shown in different colors when they are not connected inside the region of interest (black box). **(a,c,e,g)** Deposits inside the region of interest. **(b,d,f,h)** Larger FOV, including any Li connected to the deposits inside the region of interest (yellow).

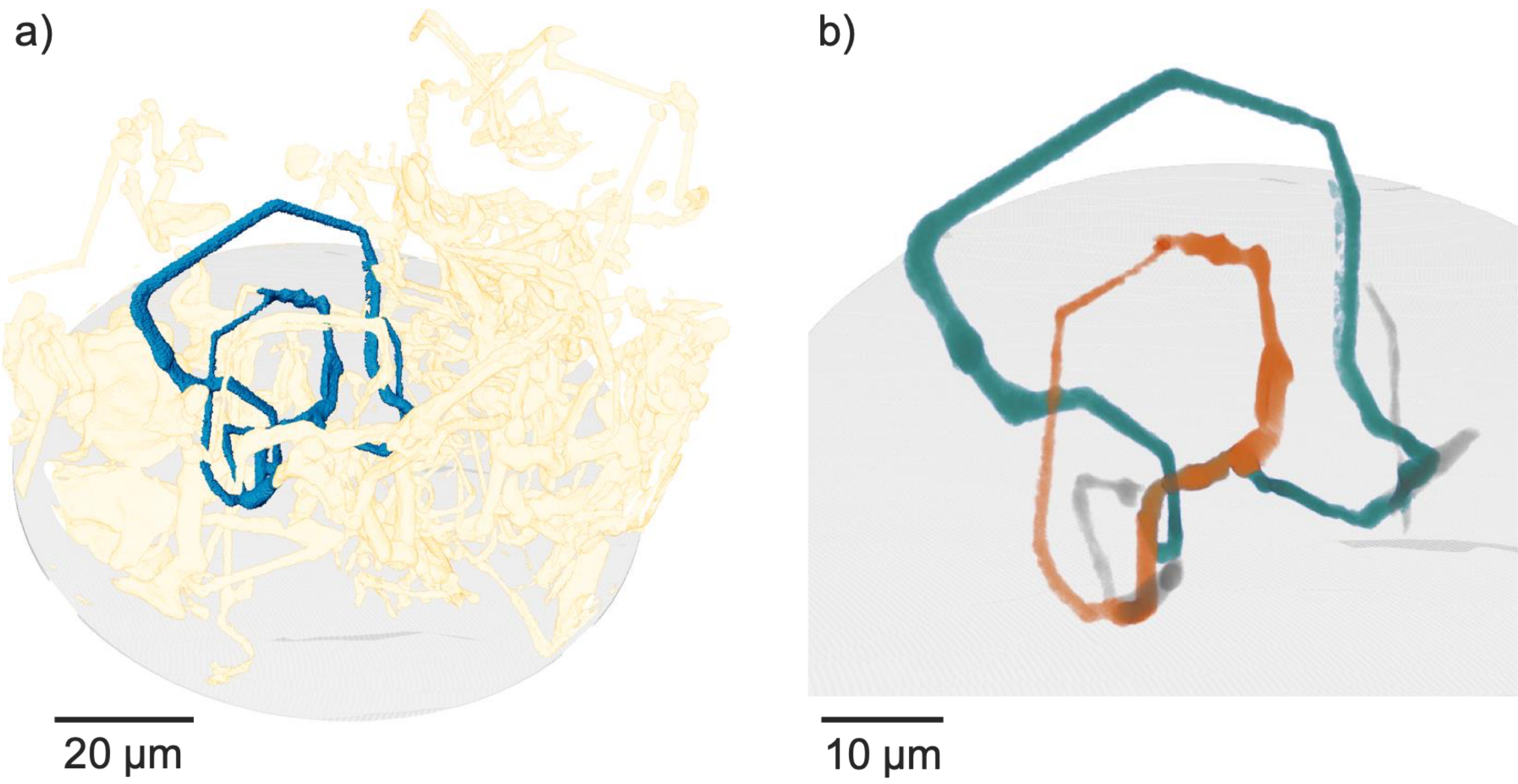

Fig. S15. Li metal structure after 24 min deposition at 1 mA/cm^2. **a)** Li branches that form loops (blue) and the surrounding Li (yellow). **b)** Enlarged view of the Li metal loops. Two examples of loops in the structure are highlighted in orange and turquoise.

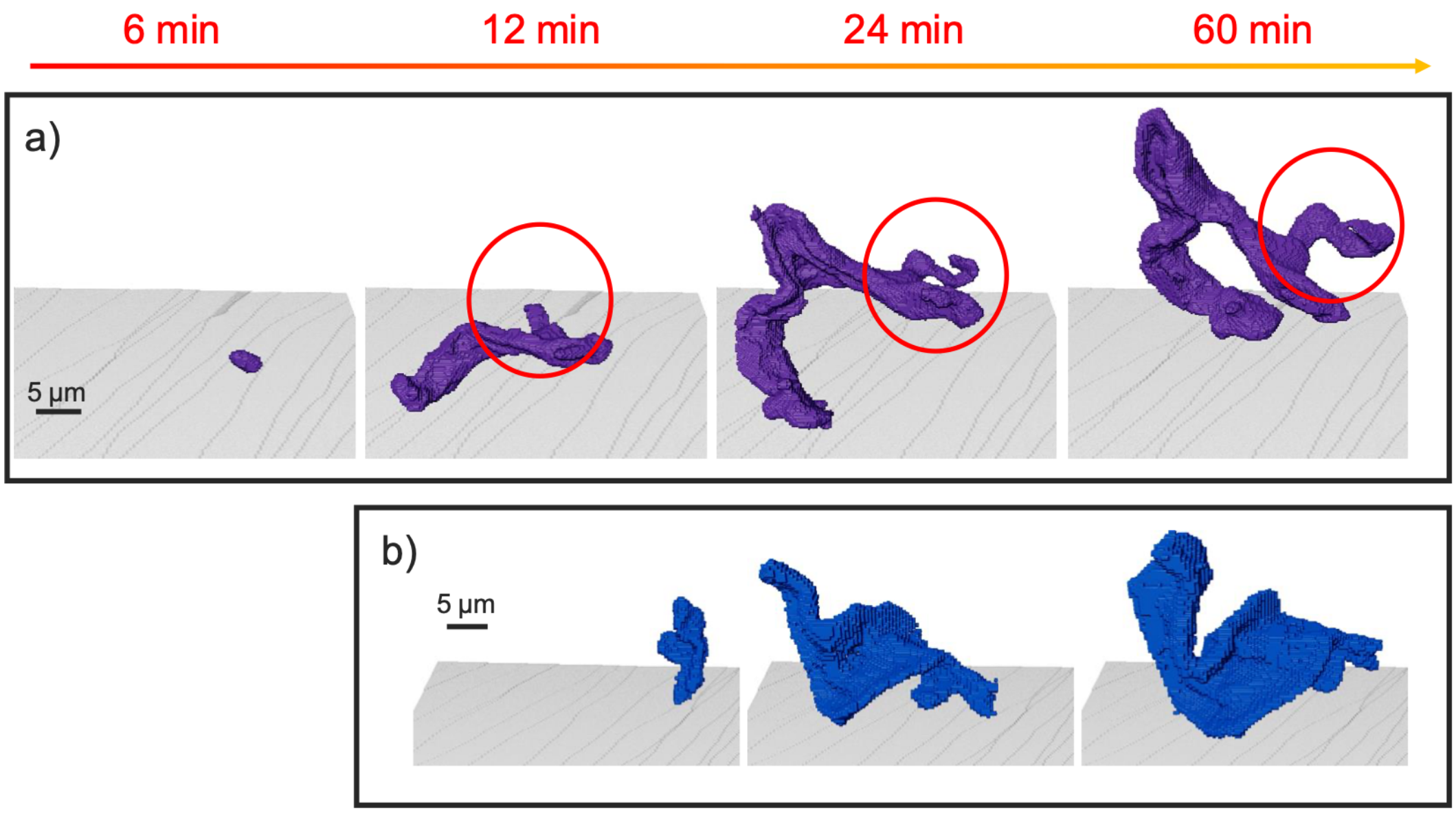

Fig. S16. Evolution of segments II **(a)** and III **(b)** of the structure shown in Fig. 6 during deposition at 1 mA/cm^2. Red circles mark the branch growing from the side of segment II as discussed in the text.

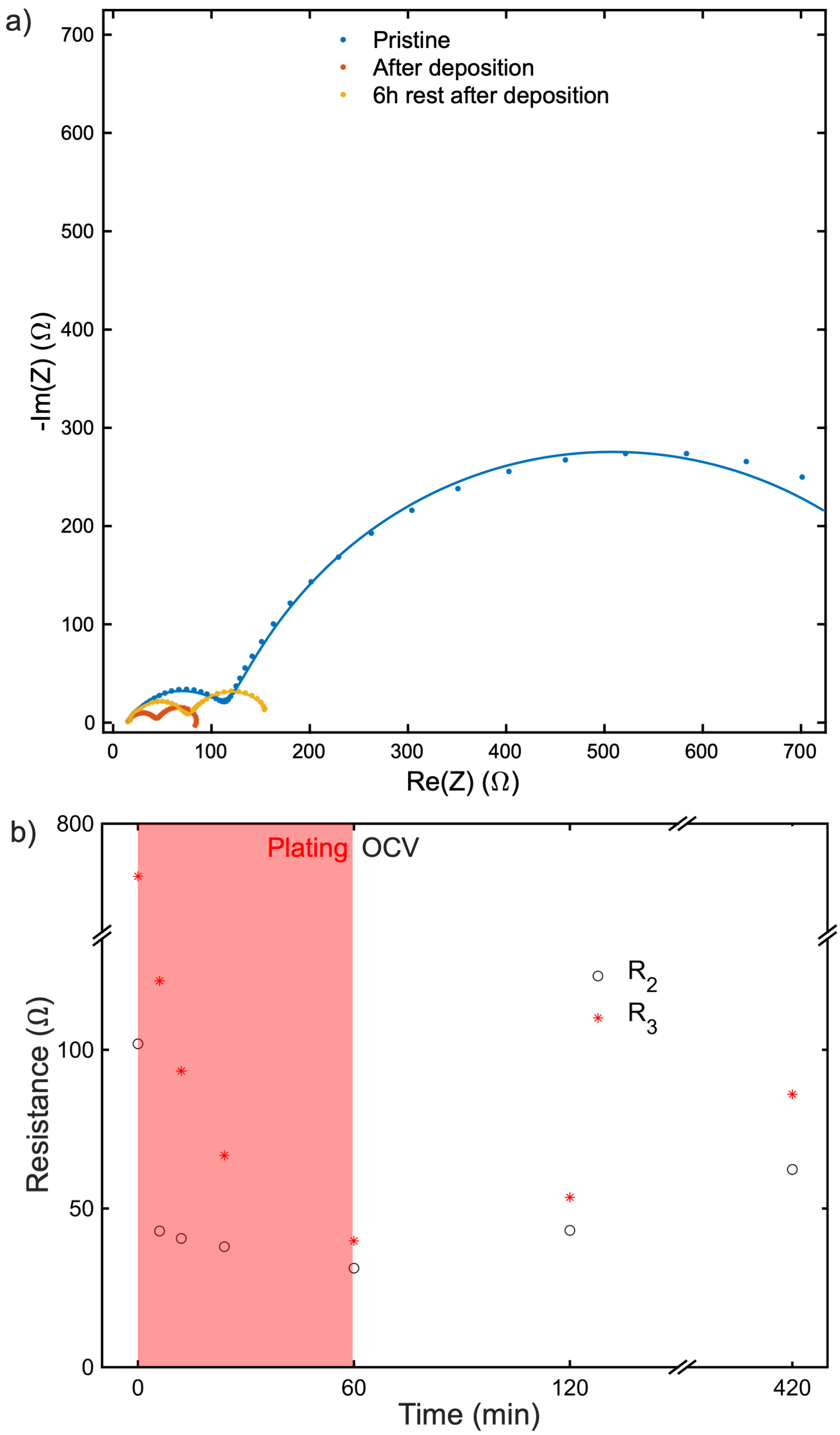

Fig. S17. Impedance evolution of a Li electrode during plating at 1 mA/cm^2. a) Impedance spectra of a pristine Li electrode, after depositing 1 mAh/cm^2 Li and subsequently resting for 6 h at OCV. Markers represent experimental data and the fitted model is shown with solid lines. b) Evolution of electrode resistances during plating and rest, fitted using an $R_1(R_2P_2)(R_3P_3)$ equivalent circuit model, where R and P denote resistances and constant phase elements, with the high- and low-frequency arcs labeled as R_2 and R_3, respectively.

Table S2. Parameters and their relative errors (ϵ) in the $R_1(R_2P_2)(R_3P_3)$ equivalent circuit model used to fit to the EIS data in Fig. S17. Q_i and n_i denote parameters of the constant phase elements, $P_i = \frac{1}{Q_i(j\omega)^i}$ with index i.

Time (min)	R_1 (Ω)	R_2 (Ω)	R_3 (Ω)	Q_2 ($\Omega^{-1}s^{n_2}$)	n_2	Q_3 ($\Omega^{-1}s^{n_3}$)	n_3	ϵ_{R_1} (%)	ϵ_{R_2} (%)	ϵ_{R_3} (%)	ϵ_{Q_2} (%)	ϵ_{n_2} (%)	ϵ_{Q_3} (%)	ϵ_3 (%)
**0**	15.2	101.9	783.0	2.86E-05	0.69	8.63E-04	0.78	1.2	1.1	1.8	6.0	0.9	1.5	0.9
**6**	15.3	42.9	121.7	4.56E-05	0.69	2.67E-03	0.75	0.6	0.8	1.0	4.8	0.7	1.5	0.9
**12**	15.4	40.6	93.3	3.77E-05	0.72	2.95E-03	0.77	0.3	0.4	0.6	2.5	0.4	0.9	0.5
**24**	15.3	38.0	66.7	4.07E-05	0.72	3.08E-03	0.82	0.5	0.7	1.1	4.9	0.7	2.0	1.0
**60**	14.5	31.2	39.8	5.37E-05	0.71	4.05E-03	0.85	0.8	1.2	1.9	8.3	1.3	3.9	1.9
**120**	14.7	43.1	53.5	3.90E-05	0.74	3.10E-03	0.87	0.6	0.8	1.6	5.7	0.8	3.1	1.5
**180**	14.8	47.7	62.1	3.55E-05	0.74	3.05E-03	0.85	0.5	0.6	1.2	4.2	0.6	2.3	1.2
**240**	15.0	52.1	68.8	3.35E-05	0.75	3.09E-03	0.84	0.4	0.5	1.1	3.3	0.5	1.9	1.0
**300**	15.1	56.0	74.0	3.20E-05	0.75	3.15E-03	0.83	0.4	0.5	1.0	3.0	0.4	1.7	0.9
**360**	15.2	59.3	80.3	3.09E-05	0.75	3.22E-03	0.81	0.3	0.4	1.0	2.7	0.4	1.6	0.9
**420**	15.4	62.3	86.0	2.98E-05	0.75	3.37E-03	0.80	0.3	0.4	1.0	2.7	0.4	1.6	0.9

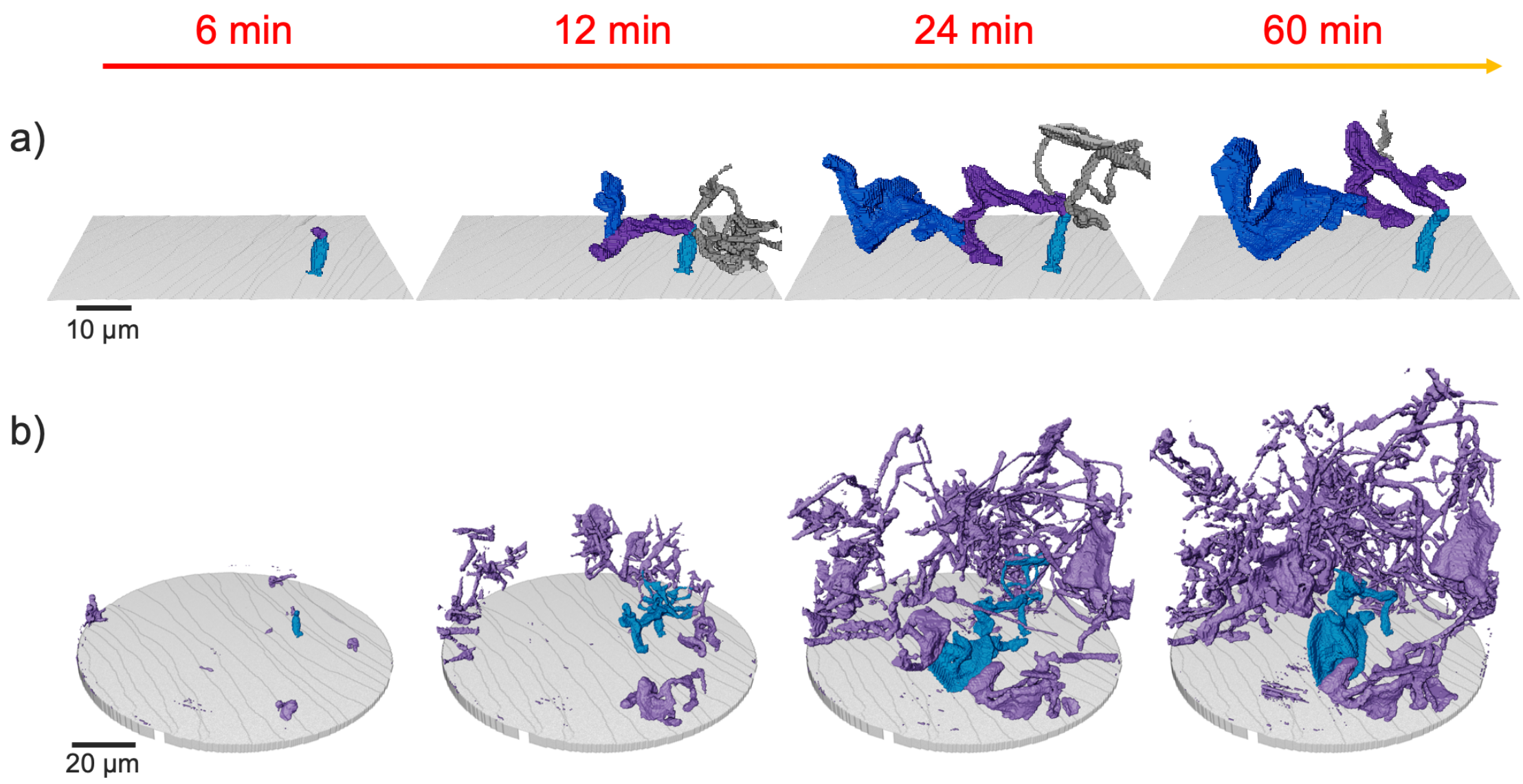

Fig. S18. Time resolved Li electrodeposition (1 mA/cm^2). **a)** The structure from Fig. 6 (light blue, dark blue and purple) is shown with other connected components (dark gray) inside the region of interest. **b)** The structure from Fig. 6 is shown in blue and the rest of the segmented Li inside the FOV of the measurement is shown in violet (electrodeposited) and light gray (bulk Li).

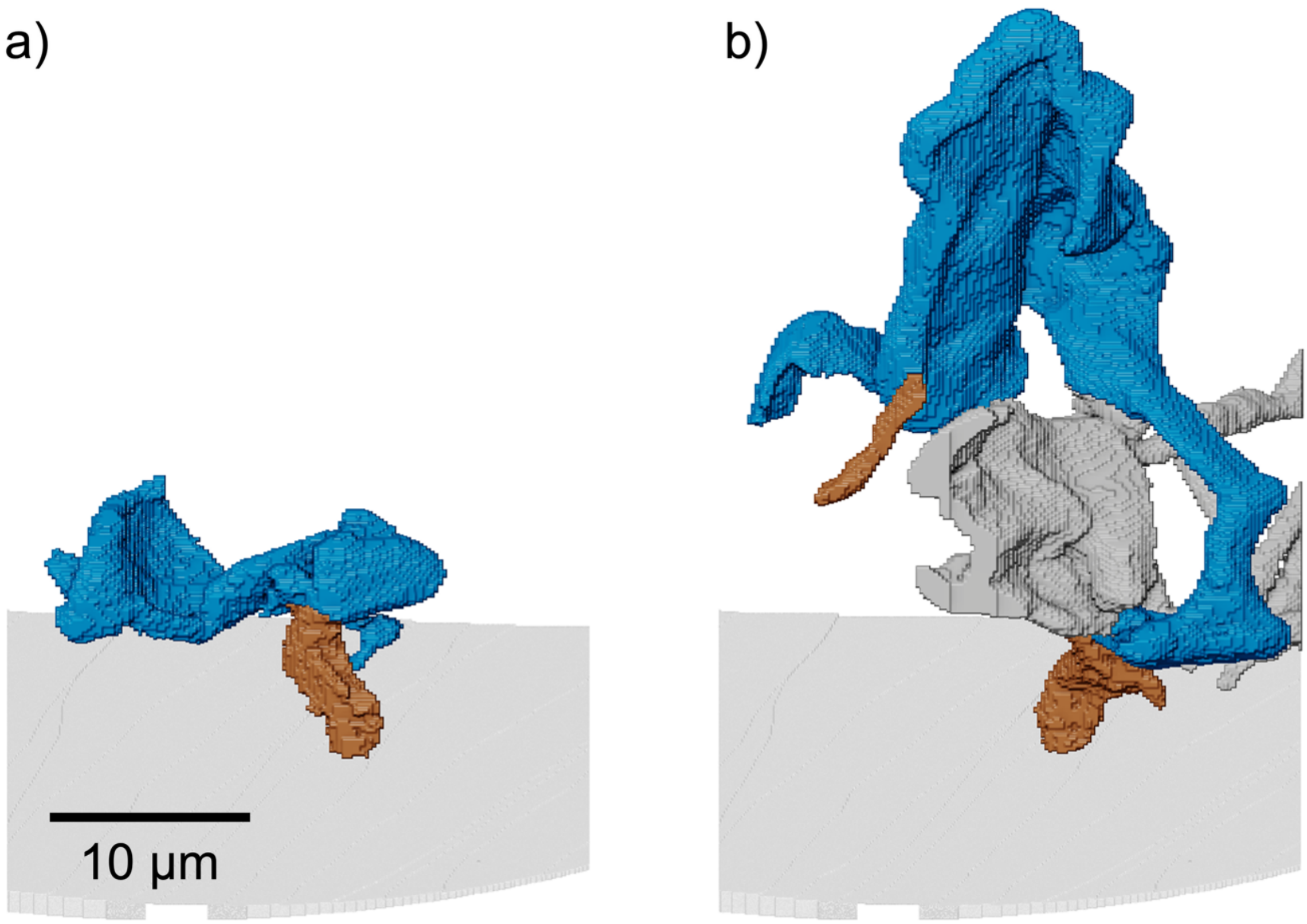

Fig. S19. Needle (brown) growing from a sheet (blue), between 12 (a) and 24 min (b) of deposition (1 mA/cm^2). Connected Li structure not of interest are shown in gray.

Supplementary Note 7: Local current density estimate

The local current density, j, in a region of the electrode can be calculated as

$$j = \frac{\Delta Q}{A\Delta t},$$

Where, A, is the area, and ΔQ is the charge passed to the electrode during the time Δt. Using Faraday's law, the expression can be rewritten as

$$j = \frac{\rho \Delta V n F}{\Delta t A M}.$$

Where ρ is the density of lithium, ΔV is the volume of lithium deposited in the time Δt, n is the number of electrons transferred in the reaction (1), F is Faraday's constant, M is the molar mass of lithium. With tomography images at two different times, t_1 and t_2, this formula can be used to estimate time-averaged local current density values at $t = (t_1 + t_2)/2$ by determining the lithium volume increases, $\Delta V = V_2 - V_1$, and average lithium surface areas $A = (A_1 + A_2)/2$. Fig. S20 shows this calculation for different segments of the lithium structure from Fig. 6. Calculations under two different assumptions are shown:

1. All of the surface area is active in the deposition, but lithium atoms are redistributed to the growth zones through diffusion (Fig. S20a)
2. The surface diffusion of lithium atoms on the structures can be neglected, which implies that the lithium must be deposited in the growth zones allowing the current density to be estimated based on the surface areas of the growth zones. For the needle (segment 1) active areas at both ends of the needle are assumed, with a cross-sectional area of roughly 3.9 μm^2, a total active area of $A = 7.8$ μm^2 is used for the calculation. For the sheets (segment 3) the growth occurs along its edges, the surface of which can be manually defined in the tomography volumes (red in Fig. S20b).

Neither of these cases are likely to be fully representative of the deposition process but enable straightforward calculations of the local current density. For both cases, the current density shows a quick decline over time. Given that the deposition current is constant, it is inevitable that the current densities decrease when the surface area of the electrode increases through inhomogeneous deposition. This trend should also be exacerbated by the gradually increasing thickness/resistivity of the SEI as the structures age.

The largest local current density for any of these segments is significantly larger than the smallest current for the other segments. This could suggest that the local current density during the growth does not determine the morphology. A possible explanation for the vastly different morphologies could be that different nucleation events dictates which morphology evolves during subsequent growth, as suggested by Wang *et al.*[23]

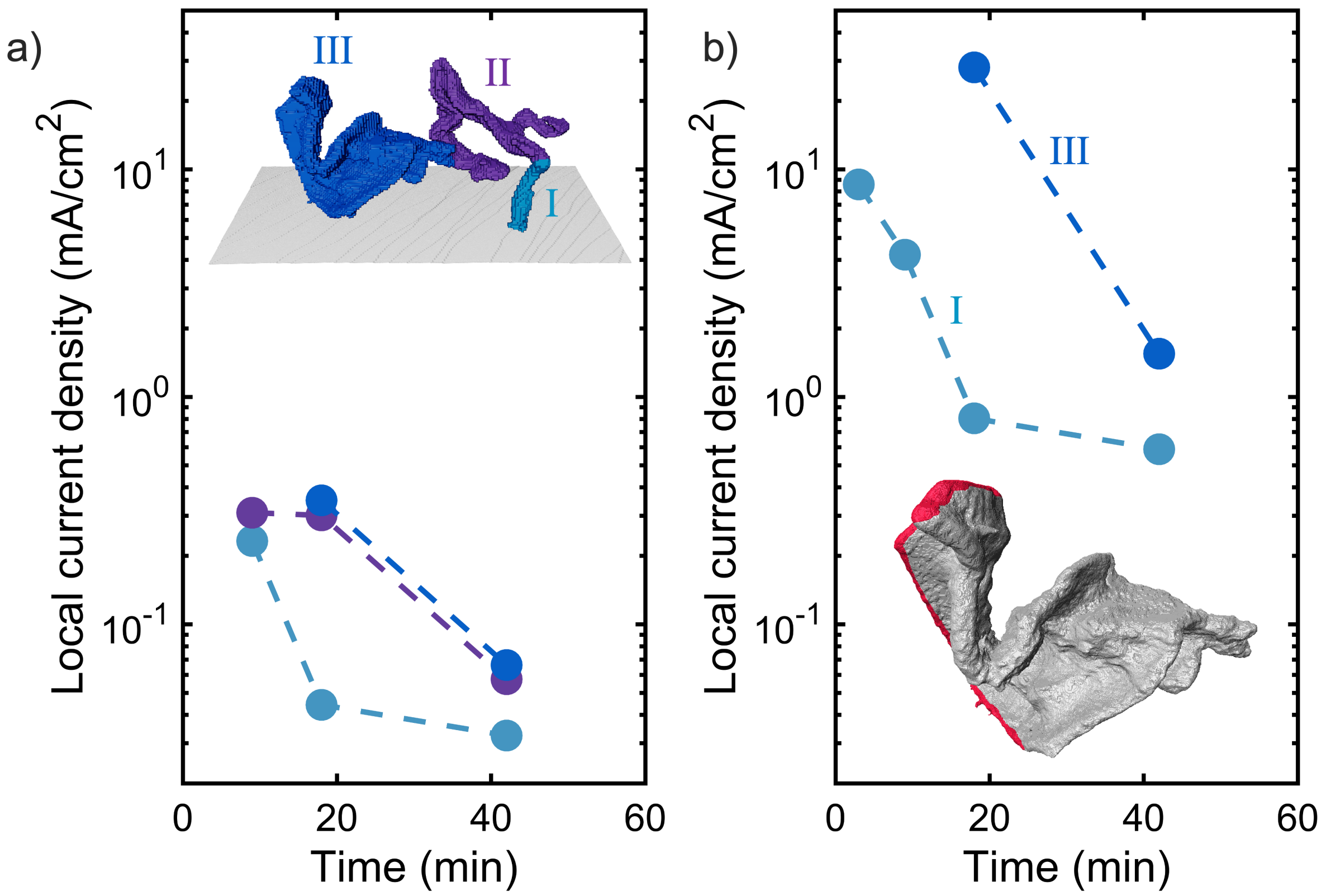

Fig. S20. Local current density estimations. a) Local current densities estimated for segment I, II and III of the structure (see inset), assuming their entire surface area is active during deposition. b) Local current densities for segment I and III, calculated using assumptions regarding which areas are active during the deposition. Inset shows active area defined for segment 3 after 60 min of deposition in red.

Supplementary Note 8: SEI characterization

Fig. S21 shows X-ray photoelectron spectra of electrodeposited Li on a Cu current collector. Since 1,2-dimethoxyethane (DME) is the sole carbon source in the electrolyte, the C1*s* spectrum reflects the solvent decomposition products in the SEI (Fig. S21a). The peak at 286.5 eV is assigned to C-O bonds, suggesting that Li alkoxides are present in the SEI, which is consistent with previous reports identifying $LiOCH_3$ as the main reduction product of DME on Li metal.[24] The peak at 289.0 eV, attributed to C=O bonds, indicates the presence of lithium carbonates or semi-carbonates ($ROCO_2Li$)[25,26] Signatures of inorganic SEI components originating from the lithium bis(fluorosulfonyl)imide (LiFSI) salt can be found in the S2*p*, N1*s*, O1*s*, and F1*s* spectra (Fig. S21b-e). Peaks with characteristic binding energies that can be ascribed to Li_2S (160.1 eV), Li_3N (396.4 eV), Li_2O (528.1 eV) and LiF (684.8 eV) as well as various oxidized sulfur SO_x (167.0 eV) and N-SO(398.3 eV) species can be observed.[27] Additionally, signatures of unreacted LiFSI can be found (Fig. S21b-e), originating from adsorbed LiFSI species remaining even after the washing. These were inhomogeneously distributed across the sample surface.

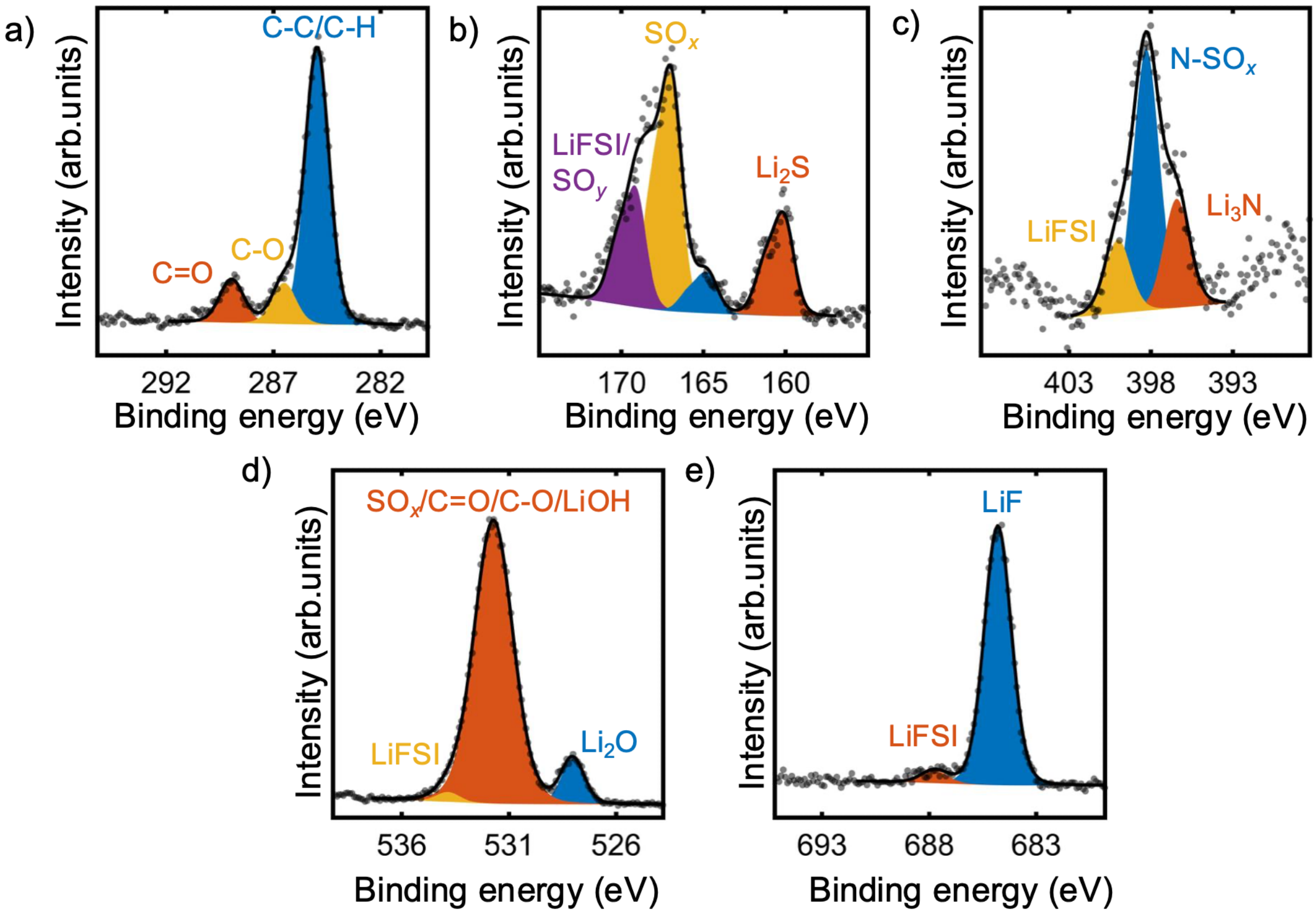

Fig. S21. X-ray photoelectron spectra of Li deposited (1 mA/cm^2) on a Cu current collector: C1*s* (a), S2*p* (b), N1*s* (c), O1*s* (d), F1*s* (e).

Supplementary Note 9: Branching

Fig. S7 shows branches (brown) that start growing from a needle. A segment between two kinks which is 20.8 µm long after 24 min (Fig. S7a) has two branches (brown) 16.8 µm apart after 60 min of deposition (Fig. S7b). This shows that at least one of the branches must have nucleated along the segment (presumably a single crystal grain) rather than at the kink (grain boundary). If lithium tends to grow through insertion at grain boundaries at kinks,[28] nucleation of a new branch along the side of a needle could be triggered by a long diffusion path to the growth zones. Note that sheet-like morphologies also can grow from the sides of needle segments (Fig. S7b).

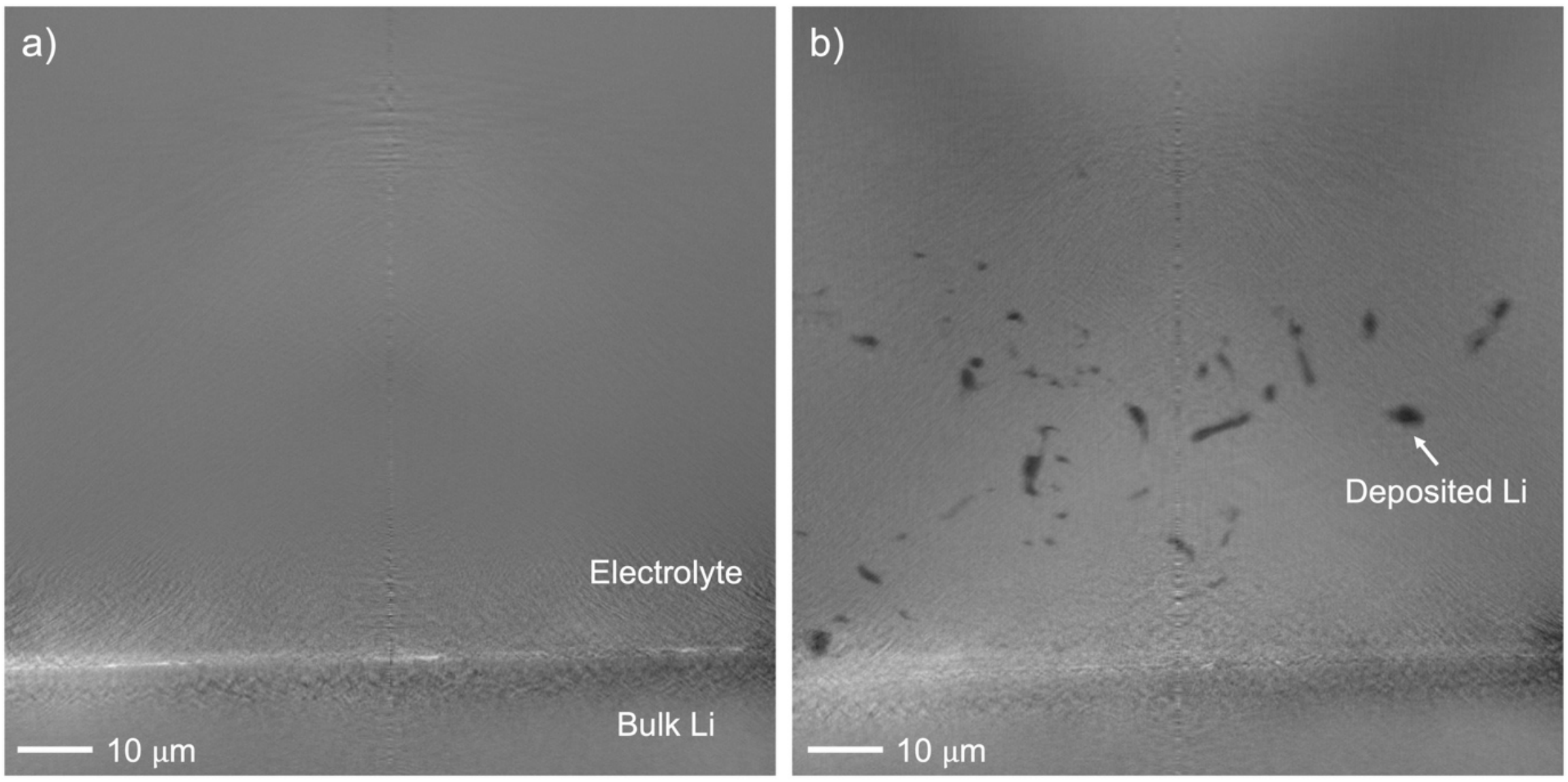

Fig. S22. Cross-sectional slices through volumes reconstructed from 50 nm voxel size holo-tomography measurements before (a) and after (b) Li electrodeposition (1 mA/cm^2 for 1 h).

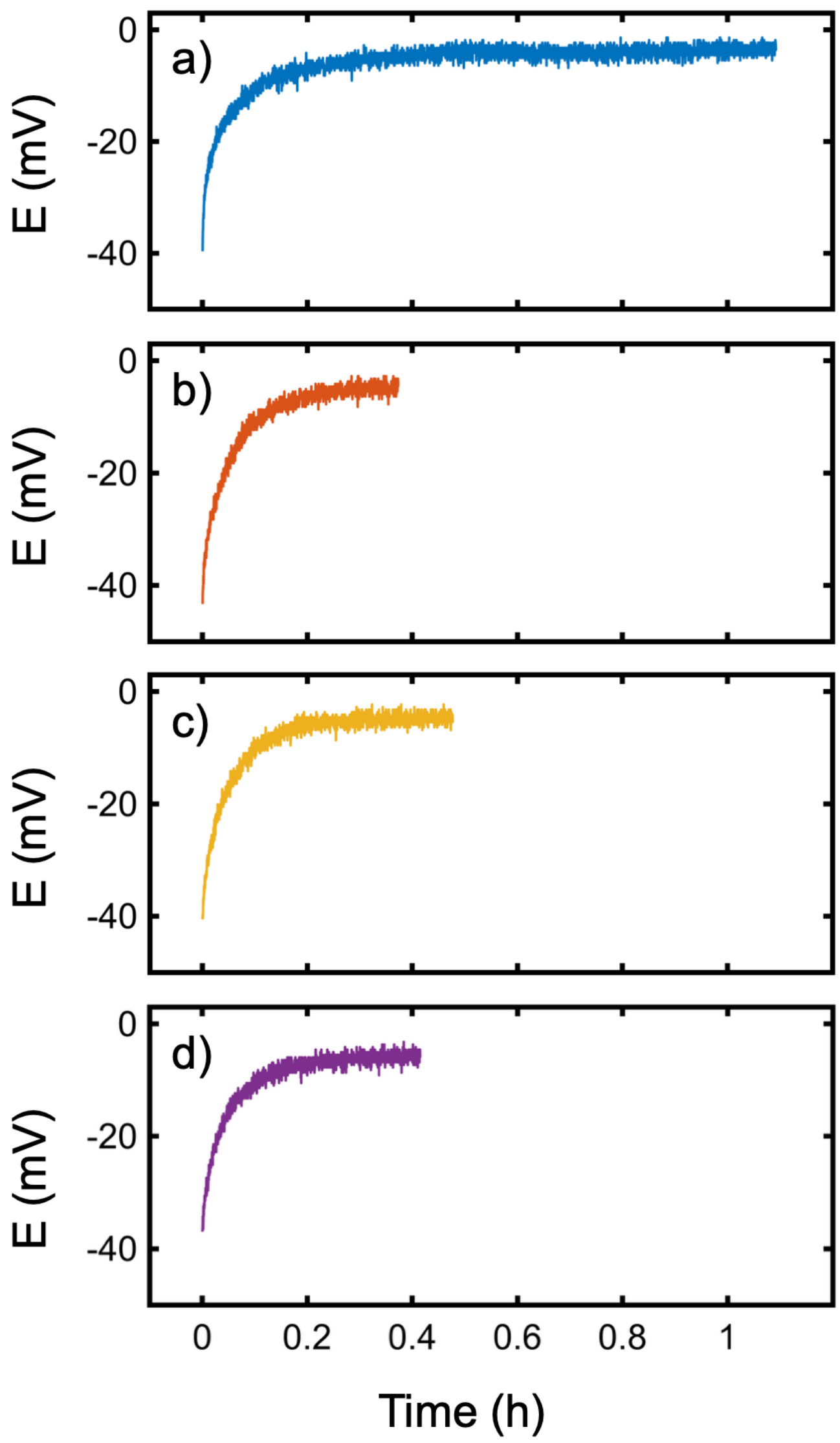

Fig. S23. Open-circuit voltage during interruptions in Li deposition, where tomography measurements were carried out, after depositing at 1 mA/cm^2 for 6 min (a), 12 min (b) 24 min (c) and 60 min (d).

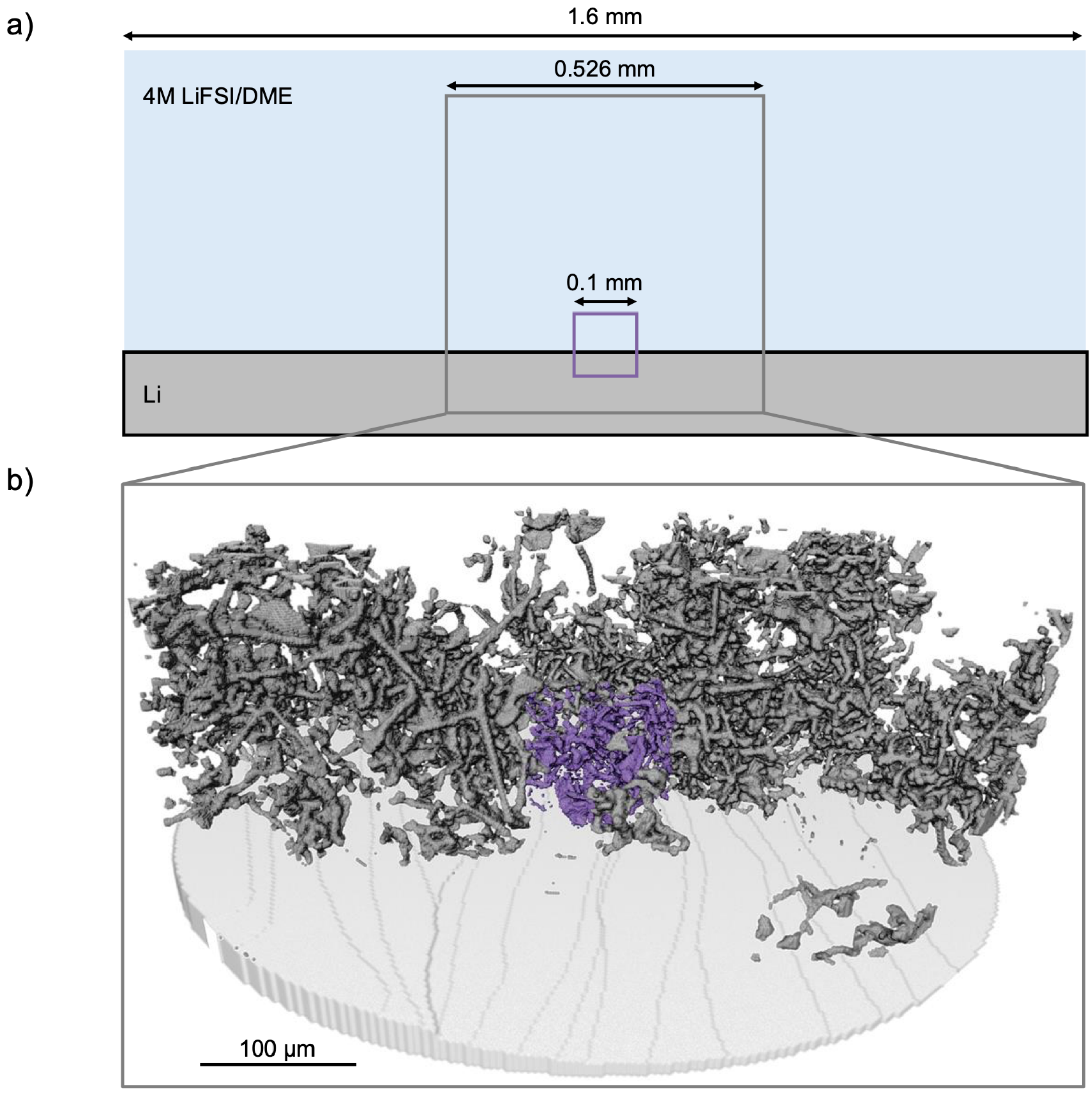

Fig. S24. Field-of-view during imaging of the lithium electrode a) Schematic drawing, with a gray square representing the volume imaged in overview measurements, and a violet square representing high resolution nano-holo-tomography. b) Volume rendering of segmented Li, after deposition at 1 mA/cm^2 for 1 h, from *in situ* nano-tomography measurements at 257 nm voxel size (from Fig. 1a). Region in the center of the volume where high resolution (50 nm, voxel size) X-ray nano-holo-tomography scans were acquired is shown in violet.

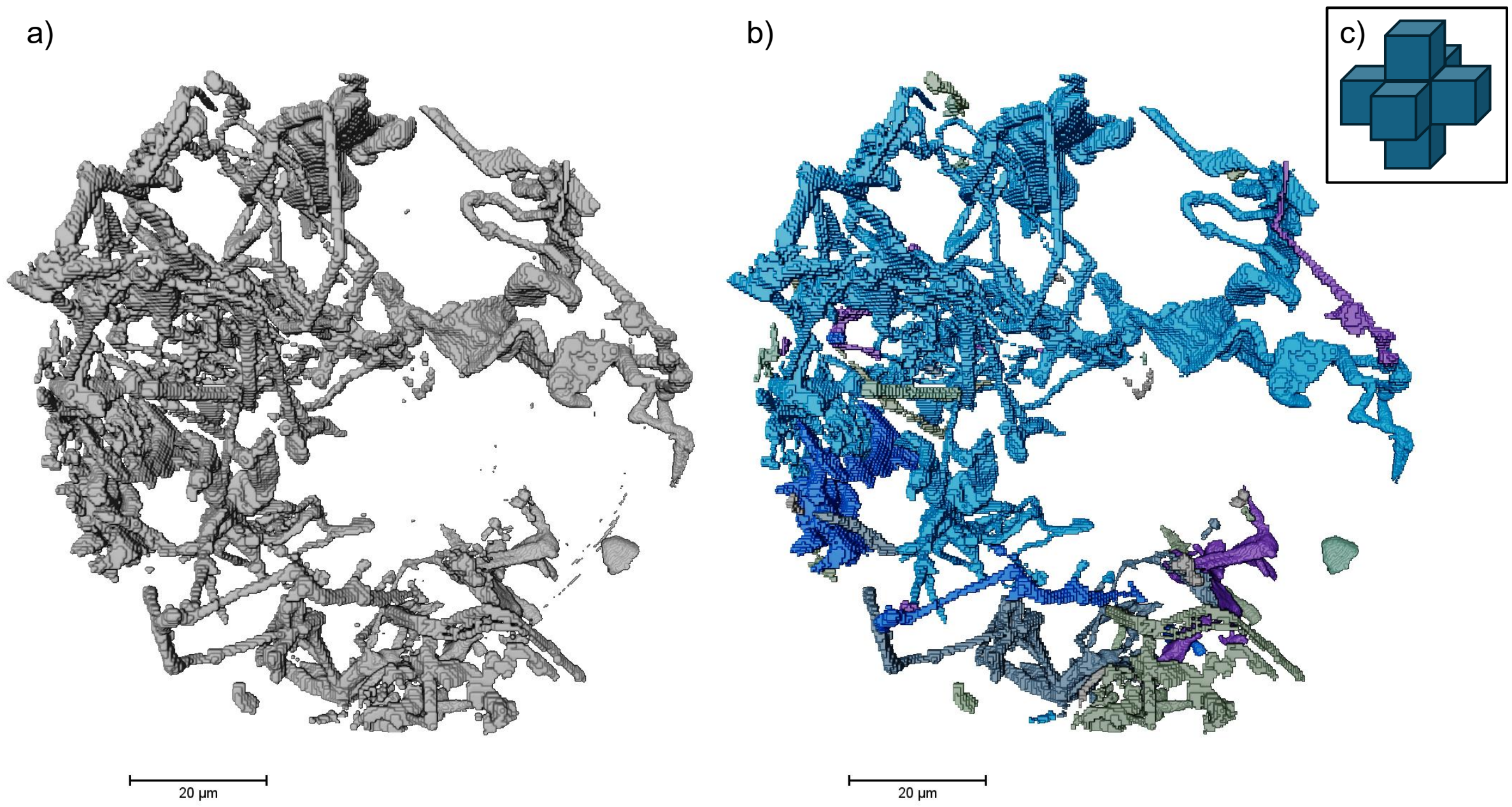

Fig. S25. Connectivity analysis. **a)** Volume rendering of segmented electrodeposited lithium. **b)** Volume rendering showing the connectivity of Li deposits from a. Li voxels connected by face are shown in the same color. Renderings and analyses in a-b are from a tomogram after 24 min deposition at 1 mA/cm^2. **c)** Voxels sharing a face with the central voxel.

References

1. Eastwood, D. S. *et al.* Three-dimensional characterization of electrodeposited lithium microstructures using synchrotron X-ray phase contrast imaging. *Chem. Commun.* **51**, 266–268 (2015).
2. Pogany, A., Gao, D. & Wilkins, S. W. Contrast and resolution in imaging with a microfocus x-ray source. *Rev Sci Instrum* **68**, 2774–2782 (1997).
3. Cloetens, P. *et al.* Holotomography: Quantitative phase tomography with micrometer resolution using hard synchrotron radiation x rays. *Appl. Phys. Lett.* **75**, 2912–2914 (1999).
4. Modregger, P., Lübbert, D., Schäfer, P. & Köhler, R. Spatial resolution in Bragg-magnified X-ray images as determined by Fourier analysis. *Physica Status Solidi (A) Applications and Materials Science* **204**, 2746–2752 (2007).
5. Chae, O. B., Kim, J. & Lucht, B. L. Modification of lithium electrodeposition behavior by variation of electrode distance. *J. Power Sources* **532**, 231338 (2022).
6. Harrison, K. L. *et al.* Effects of Applied Interfacial Pressure on Li-Metal Cycling Performance and Morphology in 4 M LiFSI in DME. *ACS Appl. Mater. Interfaces* **13**, 31668–31679 (2021).
7. Fang, C. *et al.* Pressure-tailored lithium deposition and dissolution in lithium metal batteries. *Nat. Energy* **6**, 987–994 (2021).
8. Liu, D. *et al.* Controlled large-area lithium deposition to reduce swelling of high-energy lithium metal pouch cells in liquid electrolytes. *Nat. Energy* **9**, 559–569 (2024).
9. Shea, J. *et al.* Impact of Pressure Distribution and Magnitude on the Performance of Lithium Metal Anodes. *J. Electrochem. Soc.* **171**, 020528 (2024).
10. Zhang, X. *et al.* Rethinking How External Pressure Can Suppress Dendrites in Lithium Metal Batteries. *J. Electrochem. Soc.* **166**, A3639–A3652 (2019).
11. Bai, P., Li, J., Brushett, F. R. & Bazant, M. Z. Transition of lithium growth mechanisms in liquid electrolytes. *Energy Environ. Sci.* **9**, 3221–3229 (2016).
12. Qian, J. *et al.* High rate and stable cycling of lithium metal anode. *Nat. Commun.* **6**, 6362 (2015).
13. Suo, L. *et al.* Fluorine-donating electrolytes enable highly reversible 5-V-class Li metal batteries. *Proc. Natl. Acad. Sci. U. S. A.* **115**, 1156–1161 (2018).
14. Fan, X. *et al.* Highly Fluorinated Interphases Enable High-Voltage Li-Metal Batteries. *Chem* **4**, 174–185 (2018).
15. Shi, F. *et al.* Strong texturing of lithium metal in batteries. *Proc. Natl. Acad. Sci. U. S. A.* **114**, 12138–12143 (2017).
16. Oyakhire, S. T. *et al.* Electrical resistance of the current collector controls lithium morphology. *Nat. Commun.* **13**, 3986 (2022).
17. Zachman, M. J., Tu, Z., Choudhury, S., Archer, L. A. & Kourkoutis, L. F. Cryo-STEM mapping of solid–liquid interfaces and dendrites in lithium-metal batteries. *Nature* **560**, 345–349 (2018).
18. Zheng, J. *et al.* Electrolyte additive enabled fast charging and stable cycling lithium metal batteries. *Nat. Energy* **2**, 17012 (2017).
19. Abada, S. *et al.* Safety focused modeling of lithium-ion batteries: A review. *J. Power Sources* **306**, 178–192 (2016).
20. Darling, R. & Newman, J. Modeling Side Reactions in Composite Li y Mn2 O 4 Electrodes. *J. Electrochem. Soc.* **145**, 990–998 (1998).
21. Xu, R. *et al.* Heterogeneous damage in Li-ion batteries: Experimental analysis and theoretical modeling. *J. Mech. Phys. Solids* **129**, 160–183 (2019).
22. Latz, A. & Zausch, J. Thermodynamic consistent transport theory of Li-ion batteries. *J. Power Sources* **196**, 3296–3302 (2011).

23. Wang, X. *et al.* Glassy Li metal anode for high-performance rechargeable Li batteries. *Nat. Mater.* **19**, 1339–1345 (2020).
24. Aurbach, D., Daroux, M. L., Faguy, P. W. & Yeager, E. Identification of Surface Films Formed on Lithium in Dimethoxyethane and Tetrahydrofuran Solutions. *J. Electrochem. Soc.* **135**, 1863–1871 (1988).
25. Zhang, H., Shen, C., Huang, Y. & Liu, Z. Spontaneously formation of SEI layers on lithium metal from LiFSI/DME and LiTFSI/DME electrolytes. *Appl. Surf. Sci.* **537**, 147983 (2021).
26. Eshetu, G. G. *et al.* In-Depth Interfacial Chemistry and Reactivity Focused Investigation of Lithium–Imide- and Lithium–Imidazole-Based Electrolytes. *ACS Appl. Mater. Interfaces* **8**, 16087–16100 (2016).
27. Yu, W. *et al.* Electrochemical formation of bis(fluorosulfonyl)imide-derived solid-electrolyte interphase at Li-metal potential. *Nat. Chem.* **17**, 246–255 (2025).
28. Steiger, J., Kramer, D. & Mönig, R. Mechanisms of dendritic growth investigated by in situ light microscopy during electrodeposition and dissolution of lithium. *J. Power Sources* **261**, 112–119 (2014).
