# Supplementary material for: Exploring Li growth modes in Li metal electrodes using X-ray nano-tomography
Source: Nat Commun. 2026 Sep 29;17:11233. doi: 10.1038/s41467-026-78182-9 (PMC13720151; doi:10.1038/s41467-026-78182-9)
Supplement: Supplementary file 2 [file 41467_2026_78182_MOESM2_ESM.pdf]

nature portfolio

# Peer Review File

# Exploring Li growth modes in Li metal electrodes using X-ray nano-tomography

Corresponding Author: Professor Aleksandar Matic

**This file contains all reviewer reports in order by version, followed by all author rebuttals in order by version.**

Version 0:

Reviewer comments:

Reviewer #1

(Remarks to the Author)
The manuscript presents time-resolved 3D analysis of Li metal electrodeposition in a high-concentration electrolyte using in situ X-ray nano-tomography. This approach enables both qualitative and quantitative visualization of lithium growth morphologies, distinguishing between needle-like and sheet-like structures, and linking their evolution to local current density variations and SEI dynamics. The study suggests a promising pathway to understand dendrite growth mechanisms in Li-metal batteries. However, the current version lacks sufficient discussion and supporting characterizations for the proposed mechanisms. The authors should address the following points before the manuscript can be considered for publication.

1. Needle dendrite growth mechanisms (Page 3): The claim that all three mechanisms contribute to needle growth is not strongly supported by the CT evidence. More details are required regarding the SEI morphology and the crystalline size of the Li metal anode. Additional experimental evidence and discussion are needed.
2. SEI characterization: Since dendrite growth is closely governed by the SEI, the lack of SEI characterization weakens the manuscript. If tomography can resolve SEI features, its spatial distribution and correlation with dendrite growth should be provided. Complementary chemical characterization of the SEI is also recommended.
3. Quantification of surface coverage (Fig. 2): To confirm whether the projection coverage mainly arises from elongation of initial dendrites, the authors should quantify and include the areal ratio of electrode surface covered by newly deposited Li.
4. Limitations of the in situ cell: The CT cell design differs significantly from practical cells, as it operates under zero pressure and without a separator. Both stack pressure and the separator strongly influence dendrite morphology (see doi: 10.1021/acsami.1c06488). These limitations should be explicitly discussed.
5. Beam damage concern: Synchrotron X-rays may damage the mechanical integrity Li and/or the electrolyte, raising questions on the representativeness of the results. The authors should discuss whether beam damage could affect the mechanical integrity of the electrode and electrolyte, and how this was controlled or mitigated.

Reviewer #2

(Remarks to the Author)
Review of NCOMMS-25-62476-T

Rizell et al. determined 3D structures of electrodeposited lithium as a function of time. Recently, intensive research dedicated to lithium metal batteries with solid electrolyte has shown that they are prone to metallic electrical shorts as well. Therefore, research on dendrites in liquids as done by Rizell et al. is a timely one. Due to the high resolution of the images obtained for large (> (0.1 mm)3) or huge (> (0.5 mm)3) volumes, Rizell et al. obtain useful original time-dependent data with unprecedented quality. However, the manuscript needs to be improved before it can be published: According to both abstract and conclusions, variations in the local current densities j_loc are linked to structural variations. However, it seems that no attempt has been made to substantiate this claim by estimating j_loc values. j_loc calculations are complicated by the ongoing change of the surface area and by the difficulty to decide which regions should be considered electroactive. Anyway, estimating j_loc ranges seems a pertinent analysis step. Since central aspects of wording and interpretation are incomplete, a major revision is required.

In the battery community, the definition of “anode material” and “cathode material” is based on the processes during discharge. The traditional and official meaning of “anode” (as defined in the IUPAC Compendium of Chemical Terminology, see https://goldbook.iupac.org/terms/view/A00370) is anode = electrode at which oxidation takes place. This leads to a conflict if processes during charging are considered as done here: electrodeposition is done by reduction, i.e. at a cathode.

This is not compatible with the wording used in the manuscript, e.g. in the abstract: “plated onto the anode”. Please check the manuscript and avoid or resolve this conflict at all relevant places. This can be done e.g. by using “negative electrode” instead of “anode” or “cathode”, or by precise wording, e.g. by describing lithium as “anode during discharge”; during charging, the cathodic process of electrodeposition takes place etc.

p. 4.: “The degree of crystallinity of the deposited lithium was reported to depend on the current density, with more amorphous lithium forming during deposition at low current density (0.1 mA/cm2).31

This result reported by Wang et al. (Ref. 31) seems unexpected if one assumes that crystallization might take time. Since Wang et al state “the cluster size is key”, their result might be interpreted as: larger currents lead to larger clusters, and therefore, more crystallinity. This seems to be at odds with your results: the sheets, e.g. as shown in Fig. S4 b), seem much larger than the needles shown in Fig. S4 c). If larger clusters are more crystalline, shouldn’t we expect then that the sheets are crystalline?

By using different mean (=macroscopic) current densities, Wang at al showed clearly (their Supplementary Fig. 8) that larger currents lead to more crystallinity. Why do you think that this result of different sets of experimental conditions (currents, potentials) can be interpreted as “higher _local_ current densities result in more crystallinity in an experiment where the potential is the same at all places”? Is this a result of Wang et al. or is this your interpretation of Wang’s data? Are differences in SEI thicknesses the reason for differences in local current density? Maybe it is not the local current that dictates the crystallinity, maybe it is just the other way round: Maybe high crystallinity enables faster deposition, i.e. crystallinity which might suck up mobile adatoms might be the cause of larger local currents?

A proper discussion of this issue is important in the manuscript, since the corresponding sentence in the conclusions on p. 10, “The coexistence of the two types of structures points to inhomogeneities in the local current density as sheet-like structures are expected to form at lower current density compared to need-like growth” is placed as a central part of the results. Furthermore, more quantitative results are required here. Since volume changes of the parts are known (Fig. 4) and their surface areas should be accessible as well, estimating local current densities is possible. In the simplest case of needle-like growth at constant diameter, the local current density at the tip is obtained by the formula

v = j F / Vm (2) (p. 495) of

J. L. Barton and J. O'M. Bockris, Proceedings of the Royal Society of London, Series A, Mathematical and Physical Sciences, Vol. 268, No. 1335 (Aug. 7, 1962), pp. 485-505.

Barton and Bockris studied silver deposition where it can be assumed that the silver (m.p. 1234.93 K) atoms are not very mobile. In the case of lithium, due to its high analogous temperature (m.p. 453.65 K) lithium atoms are expected to be mobile at room temperature. This means that atoms do not need to be deposited at the same place where volume growth take place, and an exact conversion of lithium volume changes to local current densities j is impossible. However, still estimated (probably upper) values of j can be given by assuming a limited range of lithium atom diffusion. These local current densities j = Q/ (t A) can be estimated from the surface areas A considered to be active (e.g. those shown in red in Fig. 6), Faraday’s law, Q = m z F / M, with m = ρ V, i.e. j = ρ V z F / (t A M). It is clear that it is unknown which part of the surface is active. However, two special cases might be considered: a) all of the surface of the structure is active, b) an area of the order of the cross-section e.g. of the structure of Fig. 4, is active. (b) corresponds to the approach of Barton and Bockris.) Although the current densities calculated might strongly depend on the assumptions made, it is still interesting to know whether there are differences between sheets and needles if the same assumptions are made. In the case that these calculations do not give a conclusive result, they still should be reported in the supplement.

p. 9 – 10, Fig. 6:

“As the deposit grows, stress will be exerted on the SEI-layer which can crack, exposing fresh Li, triggering growth of new branches (Fig. 6a).”

This text is a good description for 6 b, but it is not convincing for 6 a.

In Fig. 6 b, there is a growth zone around the sheet. Therefore, due to the growing lithium, it is clear that the SEI layer can be stressed, stretched and cracked, resulting in the growth mode as indicated. However, in Fig. 6 a), a SEI crack is shown on the side of a needle. Since there is no growth zone there, there is no reason for the SEI to crack – it should rather grow there. Could you please check in the original images that resulted in the schematic of Fig. 6 whether a surrounding needle touching the observed one might have caused the SEI to crack? If there is no such external cause of an SEI damage, an internal cause is required, e.g. a groove or a mound that results in a modified SEI or extra Li deposition. Fig. S 13 a) shows an example of a growing branch; since there is no image without the branch, the result is compatible with the view that the nucleation of the branch might have occurred already during the formation of the segment where it grows later. In Fig. 4 there is also a branching. Is is compatible with the view that the branch occurs on a mound that is present early? On a short needle segment, Li atoms deposited onto it might diffuse to growth zones nearby. If the needle gets longer, the distance to the growth zones get longer. Would it be a possible mechanism that a branch nucleates by accumulation of atoms that are too far away from a growth zone?

p. 1, and p. 10: “need-like” ?

Shouldn’t that read “needle-like” ?

p. 7:

“… the growth rate significantly decreases in the later stages of deposition for all structures despite an increasing surface area, which can be linked to the formation of a thicker more resistive SEI.”

As you explain correctly, due to the OCV periods during tomography, it is expected that the SEI thickness increase is pronounced and inevitable. However, even for constant current conditions without OCV periods, where fresh surfaces are created continously, a decrease in the growth rate can be observed, see Fig. 1 of Becherer et al., “Similarities in Lithium Growth at Vastly Different Rates”, ChemElectroChem 2021, 8, 3882 – 3893. Fig. S14 does not only demonstrate a growing

impedance during OCV, it also shows a decreasing total resistance during deposition, most likely due to an increasing surface area which results in a decreasing current density – it gets more and more difficult for the deposition to outpace the SEI growth.

p. 13: “pink beam”: since this term might be unfamiliar, a reference might be helpful, e.g. to Meents et al. 2017, https://doi.org/10.1038/s41467-017-01417-3.

Just a remark: Constructing a good electrochemical cell for lithium is difficult. Neither PEEK nor glue are expected to be stable at lithium potential – also PTFE reacts with Li.

Fig. S16. “Needle (orange)”
Please check the colors. On my screen, there is no orange needle. There is one brown structure in the left part of S16 and two brown structures in the right panel of S16.
Please label as a) and b).
"Connected Li structure not of interest are shown in grey". In the left panel, the grey structure does not seem connected, and it seems to be too narrow to be realistic. Is this an artifact which might be removed?

Reviewer #3

(Remarks to the Author)
In this manuscript, the authors tracked the evolution of Li deposition utilizing X-ray nano holotomography. Due to the difference in real and imaginary part of the refractive index between the electrolyte and Li metal, the morphology and the evolution can be visualized. Dendritic and sheet-like Li deposits were observed and tracked with lithiation.

However, similar dendritic and sheet-like deposits have been observed and 3D visualized using cryo-FIB (Nature 2018, 560, 345–349). The morphological evolution of Li deposition with a sub-micrometer resolution has also been reported (Nat Commun 14, 854 (2023)). While this paper does provide novel nano-holoCT data with Li propagation at the nanometer scale presented, it doesn't really add much impact or enlighten the community with important findings. More effort could be paid to data analysis and the mechanism. Therefore, the reviewer is not convinced that this manuscript could meet the standard and should be considered for publication in Nat. Commun.

1.Side reactions and beam damage both could lead to gas formation during the in-situ measurement. Is there any gas observed using X-ray imaging during the Li deposition? Four scans are conducted for each tomography measurement. How long is the total scan time? How do the authors deal with the potential beam damage?
2.Why a concentration of 4M LiFSI/DME electrolyte was used instead of 1 M?
3.What are the implications for the community from the phenomenon and results found in this work?
4.In Fig 5 and 6, the mechanism of the transition from Li needle to sheet is still not clear. More analysis and explanation are suggested.

Reviewer #4

(Remarks to the Author)
The authors present their work which demonstrates time-resolved, X-ray, holo-tomography of Li-dendrite growth inside of a Li-ion battery. Through this they are able to elucidate on various growth modes for Li dendrites during cycling. Notably, they claim that this is the first time-resolved tomography study on Li-dendrite growth. The direct impact is somewhat limited by the use of a 100 µm electrode gap which may alter certain effects to Li-plating (i.e., ion transport effects) as well as relegate some of the information irrelevant because li-dendrites are not allowed to grow to this size or thickness in standard cells were separators rarely extend beyond 30 µm in thickness. Regardless, I believe this work to be highly novel and an excellent addition to the literature after minor or optional revisions and/or explanations. I would mainly like to see comments 1 and 2 addressed in the manuscript
1) I would like to see comments added to the manuscript discussing the 100 µm electrode gap and its possible consequences:
a. Why was such a large gap used? (Experimental requirement?)
b. More importantly, how can this effect Li-plating and dendrite growth mechanisms? For example, the authors mention in the introduction that branched fractal structures form due to ion-transport limitations. I would assume a possible explanation why these structures are not seen here is that ion-transport was not a significant factor for this experiment because the “separator thickness” was so large. For example, 10 µm of lithium growth with a 100 µm gap reduces the ion-transport distance by 10% where as a more practical 15 µm gap would see transport reduced by 66%.
c. Why don’t you see “classical dendrites” in this experiment?
2) The authors note that Li-deposition is concentrated where the SEI is least resistive (i.e., thinnest). They further note that at OCV, cell impedance increases and that it requires 12 min to acquire a tomogram. What effects, if any, do you think this has on SEI? Would you expect Li growth to look substantially different had the tomograms been able to be acquired instantaneously?
3) Mention cell shorting as an effect t of Li-dendrites in the intro.
4) Why do you make the distinction between closed loops and connected Li structures? Shouldn’t all connected structures also be closed loops because they all originate from the same native Li Layer?
5) Does growth of Li structures stop after forming a closed loop form? I.e., is the loop actually connected electronically or does the thicker and more resistive SEI prevent electrical connection when the loop is formed? Do you have enough data to

monitor the evolution of loop structures or only enough the see that they eventually form.
6) Explain why the Li-layer gets thicker while simultaneously getting more dense near the CC side. Shouldn't this conflict with the conclusion that there is limited growth on older Li structures due to a thicker and more resistive SEI?
7) Can you elaborate on the needle-to-sheet transition in growth modes? Did you observe enough of these transitions to identify any patterns or indicators for when this transition occurs?
8) Did the cell ever electrically short?
9) While likely outside the scope of work, given this was a synchrotron experiment, I would be interested to see future work where the experiment is extended to stripping, both of the native Li layer as well as stripping of plated dendrite features. Would stripping also be localized to the red areas in fig 6 where the SEI is assumed thinnest? Would an SEI shell be left behind and subsequently affect additional plating? Interesting questions.

Version 1:

Reviewer comments:

Reviewer #1

(Remarks to the Author)
The authors have addressed all of my concerns, and I am pleased to recommend this manuscript for publication in Nature Communications.

Reviewer #2

(Remarks to the Author)
The authors have improved their manuscript considerably. They strengthened the line of arguments by additional evaluations and clarified their conclusions. Therefore, the manuscript can be accepted for publication after some minor improvements.

These minor modifications are suggested:

p. 1.
and risks of short circuiting the cell.
instead of
and risks short circuiting the cell.

through which the Li-ions must be transported to reach the electrode
instead of
which the Li-ions must be transported through to reach the electrode

cracks in the SEI result in hotspots for deposition
The authors might consider to add an additional reference here, strongly supporting the "cracks causes dendrites" hypothesis:
Doron Aurbach, Ella Zinigrad, Yaron Cohen, Hanan Teller,
A short review of failure mechanisms of lithium metal and lithiated graphite anodes in liquid electrolyte solutions
Solid State Ionics 148 (2002) 405– 416

Supplementary Note 4:
$z_{\mathrm{Li}}$ is the charge number of the Li ion ($z_{\mathrm{Li}} = 1$),
instead of
$z_{Li}$ is the charge number of the Li ion (1),

Supplementary Note 9:
Since several time steps are considered, the equations should rather read
$j = \Delta Q / A\, \Delta t$
instead of
$j = Q / t\, A$
and
$j = \rho\, \Delta V\, n\, F / \Delta t\, A\, M$
instead of
$j = \rho\, V\, n\, F / t\, A\, M$.
Since the variables are written in italics in the equations, variables in the following text should also be in italics (times, $t_1$ and $t_2$, $t=(t_1+t_2)/2$, $\Delta V= V_2-V_1$, $A=(A_1+A_2)/2$.

To emphasize that lithium atoms diffuse on the surface, one might write
but lithium atoms are redistributed to the growth zones through diffusion
instead of

but lithium is redistributed to the growth zones through diffusion
and
The surface diffusion of lithium atoms on the structures
instead of
The lithium surface diffusion on the structures

Since the publisher will not edit the supplementary material, is suggested to check and to improve the typesetting of the equations. Usually, functions like sinh and operators like div and grad are not in italic (e.g. \sinh might be used in LaTeX).
In the text,
Equation (1)
might be used instead
Equation 1
(correspondingly for all links to the equations).
Usually, equation numbers are not in italics.
In the lines
η is the overpotential η = E - Eeq = ϕS - ϕel for the lithium
and
Making the assumption that α = 1 - β = 0.5
please use minus signs instead of the dashes.

Reviewer #3

(Remarks to the Author)
The authors have addressed the questions and concerns in this revision. The manuscript has been improved. Although a new characterization method was used, similar findings have been reported, reducing the novelty of the work. In addition, several critical questions should be well addressed.

1. Regarding the novelty of the work: several studies on the Li morphology evolution and the mechanism underlying have been reported at nano-meter spatial resolution and/or under in-situ conditions. What are the differences of the Li deposition mechanism that are obtained from this work compared to previous reports (Nano Energy 32 (2017) 271–279; ACS Energy letters, 2021, 6, 1719-1728.)?

2. The authors state that no gas was observed in the experiments. Images at the initial stage and at the end of the experiment are suggested to be provided in the SI.

3. The in-situ cell has a diameter of 1.6 mm. But a field of view around 100 µm was shown in the manuscript. Which part of the in-situ cell is shown in the work? As lithium-ion diffusion could be different in different areas in the cell and thus lead to varying deposition morphology, it is therefore not clear if the selected field of view shown in the paper representative? How about the lithium deposition at the other regions within the cell?

4. As the lithium deposition behavior differs significantly under different chemical and pressure conditions. A concentration of 4M LiFSI/DME electrolyte and a zero-pressure setup that were used in the experiment are rarely used in lithium battery research. (The limitation of the setup was also mentioned by reviewer #1.) Therefore, these two limitations greatly lower the impact of the phenomenon observed and the results obtained in this work. To ensure the novelty of the work, trial experiments using in-situ setups with pressure or widely used electrolytes (like 1M $LiPF_6$/EC+DEC, 1M LiTFSI/DOL+DME, etc.) should be added.

Reviewer #4

(Remarks to the Author)
The authors have addressed all of my comments, and I believe the manuscript is ready for publication in its current form. I thank the authors for the careful consideration of my comments and the additions they made to the manuscript.

Version 2:

Reviewer comments:

Reviewer #3

(Remarks to the Author)
The authors have addressed the questions. The manuscript could be considered for publication.

**Open Access** This Peer Review File is licensed under a Creative Commons Attribution 4.0 International License, which permits use, sharing, adaptation, distribution and reproduction in any medium or format, as long as you give appropriate credit to the original author(s) and the source, provide a link to the Creative Commons license, and indicate if changes were made.
In cases where reviewers are anonymous, credit should be given to 'Anonymous Referee' and the source.
The images or other third party material in this Peer Review File are included in the article's Creative Commons license, unless indicated otherwise in a credit line to the material. If material is not included in the article's Creative Commons license and your intended use is not permitted by statutory regulation or exceeds the permitted use, you will need to obtain permission directly from the copyright holder.
To view a copy of this license, visit https://creativecommons.org/licenses/by/4.0/

# A Point-by-Point Response to Reviewers comments on manuscript ID NCOMMS-25-62476-T

**Manuscript ID:** NCOMMS-25-62476-T
**Title:** Revealing Li growth modes using X-ray nano-tomography
**Authors:** Josef Rizell, Victor Vanpeene, Martina Olsson, Jan Jamroz, François Cadiou, Hervé Manzanarez, Valentin Vinci, Olga Stamati, Julie Villanova, Sandrine Lyonnard, Aleksandar Matic

## Response to Reviewer #1:

*The manuscript presents time-resolved 3D analysis of Li metal electrodeposition in a high-concentration electrolyte using in situ X-ray nano-tomography. This approach enables both qualitative and quantitative visualization of lithium growth morphologies, distinguishing between needle-like and sheet-like structures, and linking their evolution to local current density variations and SEI dynamics. The study suggests a promising pathway to understand dendrite growth mechanisms in Li-metal batteries. However, the current version lacks sufficient discussion and supporting characterizations for the proposed mechanisms. The authors should address the following points before the manuscript can be considered for publication.*

**Authors' response:**
We thank the reviewer for the constructive feedback which we have addressed in the subsequent comments.

*1. Needle dendrite growth mechanisms (Page 3): The claim that all three mechanisms contribute to needle growth is not strongly supported by the CT evidence. More details are required regarding the SEI morphology and the crystalline size of the Li metal anode. Additional experimental evidence and discussion are needed.*

**Authors' response:**
We have strengthened the arguments regarding the needle growth mechanism to highlight observable facts in the CT images on pages 4 and 9 in the revised manuscript. Stress driven extrusion leads to base growth, but we have added additional evidence, see new figure S7, that the needles also grow from other parts than the base. This means that alternative driving forces (such as crystal anisotropy, negative surface charge, and/or local SEI composition) are also responsible for the needle growth. To further support claims on growth modes additional XPS experiments have also been performed to provide details on the SEI composition, supporting information page 33, which are also correlated to information on thickness and structure from TEM experiments reported in literature (new references [35] added).

**Manuscript edits on page 4:**

While stress-driven extrusion of lithium (ii) could explain the initiation of needle-type structures, including branching and would favor base growth, **Fig. S7** shows that segments in the middle of lithium needles elongate and branch during deposition. This indicates that a negatively charged surface (i) and/or crystal anisotropy (iii) should also be important drivers for the elongation of needle segments during lithium growth.

**Manuscript edits on page 9:**

To trigger different types of growth, changes/cracks in the SEI-layer could impact the nucleation of new growth sites. This electrolyte forms a roughly 10 nm thick, amorphous SEI,[35] containing several inorganic components, such as LiF, $Li_2S$, $Li_2O$ and $Li_3N$, as determined by X-ray photoelectron spectroscopy experiments (**Fig. S22, Supplementary Note 10**). This type of interphase can be expected to be brittle and thereby prone to crack due to stress buildup during growth.[36]

[35] Chen, Y. et al. Origin of dendrite-free lithium deposition in concentrated electrolytes. Nat. Commun. 14, 2655 (2023).

**Manuscript edits on supporting information page 11:**

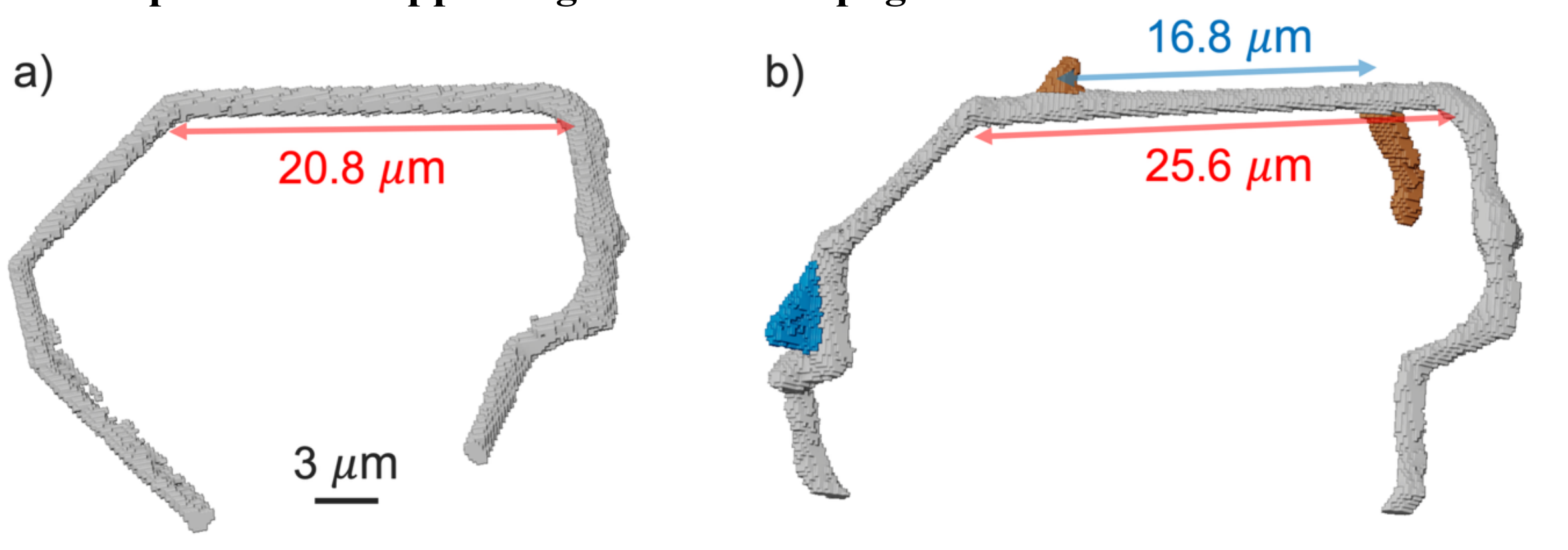


**Fig. S7**. Nucleation of new branches and elongation of a Li-needle. **a)** A needle found after 24 min of deposition. **b)** The same needle after 60 min of deposition. New needle-like branches are shown in brown and a sheet growing from the side of a segment is shown in blue.

**Manuscript edits on supporting information page 31:**

**Supplementary Note 10: SEI characterization**

**Fig. S22** shows X-ray photoelectron spectra of electrodeposited Li on a Cu current collector. Since DME is the sole carbon source in the electrolyte, the C1s spectrum reflects the solvent decomposition products in the SEI (Fig. S22a). The peak at 286.5 eV is assigned to C-O bonds, suggesting that Li alkoxides are present in the SEI, which is consistent with previous reports identifying $LiOCH_3$ as the main reduction product of DME on Li metal.[17] The peak at 289.0 eV, attributed to C=O bonds, indicates the presence of lithium carbonates or semi-carbonates ($ROCO_2Li$)[18,19] Signatures of inorganic SEI components originating from the LiFSI salt can be found in the S2p, N1s, O1s, and F1s spectra (Fig. S22b-e). Peaks with characteristic binding energies that can be ascribed to $Li_2S$ (160.1 eV), $Li_3N$ (396.4 eV), $Li_2O$ (528.1 eV) and LiF (684.8 eV) as well as various oxidized sulfur $SO_x$ (167.0 eV) and N-SO(398.3 eV) species can be observed.[20] Additionally, signatures of unreacted LiFSI can be found (Fig. S22b-e), originating from adsorbed LiFSI species remaining even after the washing. These were inhomogeneously distributed across the sample surface.

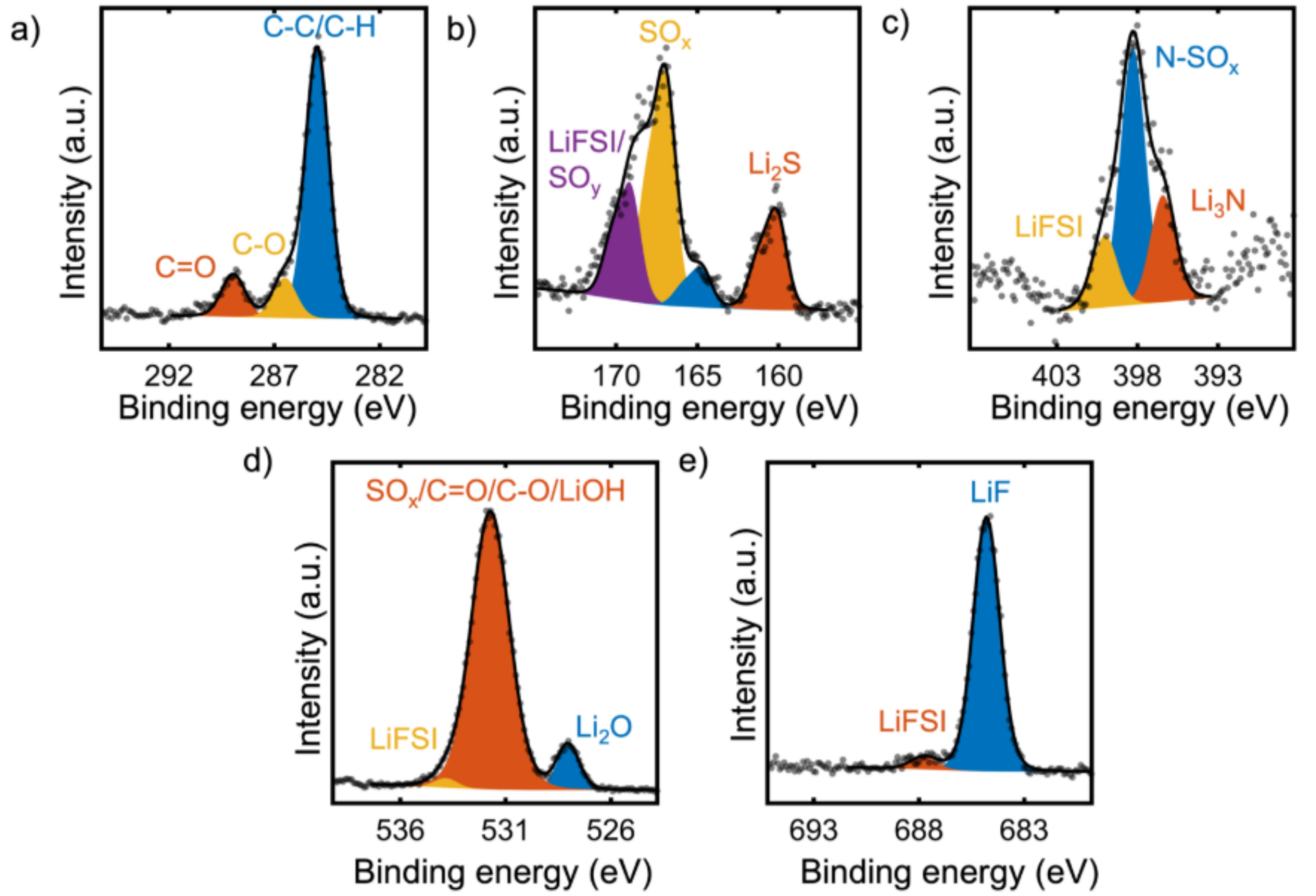


**Fig. S22**. XPS spectra of Li deposited on a Cu current collector: C1s (a), S2p (b), N1s (c), O1s (d), F1s (e).

*2. SEI characterization: Since dendrite growth is closely governed by the SEI, the lack of SEI characterization weakens the manuscript. If tomography can resolve SEI features, its spatial distribution and correlation with dendrite growth should be provided. Complementary chemical characterization of the SEI is also recommended.*

**Authors' response:**

The spatial resolution (150 nm) in our tomography experiment is not sufficient to resolve the SEI and its structure, as the SEI forming on lithium metal in this electrolyte is around 10 nm thick (reference [35] in the revised manuscript). However, information on the SEI composition obtained from XPS experiments has been added to further support our claims. The results clearly show the presence of several inorganic components such as LiF, $Li_2S$, $Li_2O$ and $Li_3N$, which suggests that the SEI can be expected to be brittle.

**References**

[35] Chen, Y., Li, M., Liu, Y. et al. Origin of dendrite-free lithium deposition in concentrated electrolytes. Nat Commun 14, 2655 (2023). https://doi.org/10.1038/s41467-023-38387-8

**Manuscript edits on page 9:**

To trigger different types of growth, changes/cracks in the SEI-layer could impact the nucleation of new growth sites. This electrolyte forms a roughly 10 nm thick, amorphous SEI,[35] containing several inorganic components, such as LiF, $Li_2S$, $Li_2O$ and $Li_3N$, as determined by X-ray photoelectron spectroscopy experiments (**Fig. S22, Supplementary Note 10**). This type of interphase can be expected to be brittle and thereby prone to crack due to stress buildup during growth.[36]

**Manuscript edits on supporting information page 31:**
**Supplementary Note 10: SEI characterization**
**Fig. S22** shows X-ray photoelectron spectra of electrodeposited Li on a Cu current collector. Since DME is the sole carbon source in the electrolyte, the C1s spectrum reflects the solvent decomposition products in the SEI (Fig. S22a). The peak at 286.5 eV is assigned to C-O bonds, suggesting that Li alkoxides are present in the SEI, which is consistent with previous reports identifying $LiOCH_3$ as the main reduction product of DME on Li metal.[17] The peak at 289.0 eV, attributed to C=O bonds, indicates the presence of lithium carbonates or semi-carbonates ($ROCO_2Li$)[18,19] Signatures of inorganic SEI components originating from the LiFSI salt can be found in the S2p, N1s, O1s, and F1s spectra (Fig. S22b-e). Peaks with characteristic binding energies that can be ascribed to $Li_2S$ (160.1 eV), $Li_3N$ (396.4 eV), $Li_2O$ (528.1 eV) and LiF (684.8 eV) as well as various oxidized sulfur $SO_x$ (167.0 eV) and N-SO(398.3 eV) species can be observed.[20] Additionally, signatures of unreacted LiFSI can be found (Fig. S22b-e), originating from adsorbed LiFSI species remaining even after the washing. These were inhomogeneously distributed across the sample surface.

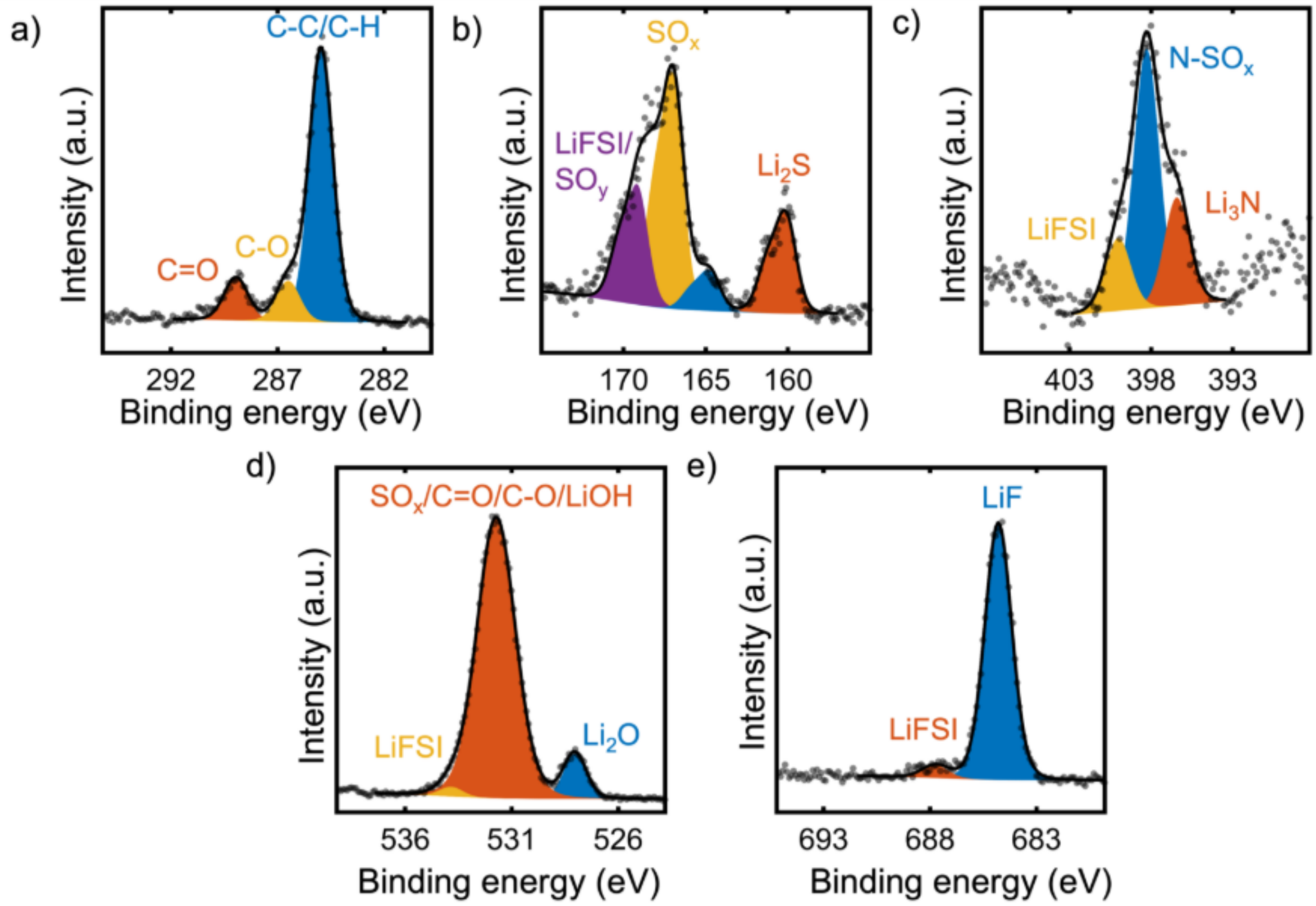


**Fig. S22**. XPS spectra of Li deposited on a Cu current collector: C1s (a), S2p (b), N1s (c), O1s (d), F1s (e).

*3. Quantification of surface coverage (Fig. 2): To confirm whether the projection coverage mainly arises from elongation of initial dendrites, the authors should quantify and include the areal ratio of electrode surface covered by newly deposited Li.*

**Authors' response:** With the growth of structures occurring at different segments simultaneously (see Fig. 6), it is not trivial to isolate only the freshly deposited lithium (i.e. the Li deposited since the last tomogram was acquired) to estimate its surface coverage. The straightforward approach of calculating the difference between the areal

capacities at each timepoint (Fig. 2e-h) would result in negative areal capacities in some parts of the electrode, because old lithium structures move when lithium gets deposited anywhere but the tip of the structure.

Instead, the growth of lithium structures through elongation and/or planar expansion (of sheets) can be confirmed using Figure 3c (local thickness of lithium deposits) and Figure S9 (intersection between bulk and deposit). If the needles grow with minor thickness increase, and no new growth sites appear on the bulk electrode, the existing needles must be elongating and branching. This has been clarified in the manuscript on page 5.

**Manuscript edits on page 5**:
The average local thickness increases only slightly, from 1.40 µm ($\sigma$=0.71 µm) to 1.55 µm ($\sigma$=0.62 µm) between 6 and 60 min of deposition (**Fig. S10**). The almost constant average local thickness, and lack of new growth sites on the bulk electrode (Fig. S9), show that Li grows mainly through branching, elongation of branches or planar growth, without significant coarsening of the structures.

*4. Limitations of the in situ cell: The CT cell design differs significantly from practical cells, as it operates under zero pressure and without a separator. Both stack pressure and the separator strongly influence dendrite morphology (see doi: 10.1021/acsami.1c06488). These limitations should be explicitly discussed.*

**Authors' response:** Indeed, cell configuration and pressure both have impact on the deposited Li morphology. Here, we would like to clarify that this experiment serves as a model one to explore the intrinsic growth mode of lithium prior to evaluate the pressure-induced impact on the morphology. We have clarified in the main text on page 2 that the cell operates without pressure and added a discussion on the effects of pressure in supporting information on page 6.

**Manuscript edits on page 2:**
Results from *in situ* X-ray nano-tomography during galvanostatic cycling of Li metal symmetric cells in a pressure free configuration (**Fig. S1, Supplementary Note 3**) are shown in **Fig. 1**.

**Manuscript edits in supporting information on page 6:**
**Supplementary Note 3: Cell configuration**
The tomography cell operates with no applied stack pressure, and a large electrode separation (≈1 mm). This ensures that cells do not short circuit while keeping electrode separation small enough to avoid ion depletion at the electrode surface (see Supplementary Note 4). Still, the absence of stack pressure is a clear difference between this cell and common lithium metal cell formats, where the pressure and type of separator have a strong influence on the morphology of the deposited lithium.[5] Generally, a more compact morphology with larger grains form under pressure.[6] However, the deposition without pressure is still important to understand for two reasons: 1) the driving forces controlling the lithium metal deposition are easier to disentangle in this more idealized scenario and 2) local differences in pressure are common in both coin and pouch cells,[7–9] which means that it is important to understand how lithium grows also in regions with little or no pressure.

5. Harrison, K. L. et al. Effects of Applied Interfacial Pressure on Li-Metal Cycling Performance and Morphology in 4 M LiFSI in DME. ACS Appl. Mater. Interfaces 13, 31668–31679 (2021).
6. Fang, C. et al. Pressure-tailored lithium deposition and dissolution in lithium metal batteries. Nat. Energy 6, 987–994 (2021).
7. Liu, D. et al. Controlled large-area lithium deposition to reduce swelling of high-energy lithium metal pouch cells in liquid electrolytes. Nat. Energy 9, 559–569 (2024).
8. Shea, J. et al. Impact of Pressure Distribution and Magnitude on the Performance of Lithium Metal Anodes. J. Electrochem. Soc. 171, 020528 (2024).
9. Zhang, X. et al. Rethinking How External Pressure Can Suppress Dendrites in Lithium Metal Batteries. J. Electrochem. Soc. 166, A3639–A3652 (2019).

*5. Beam damage concern: Synchrotron X-rays may damage the mechanical integrity Li and/or the electrolyte, raising questions on the representativeness of the results. The authors should discuss whether beam damage could affect the mechanical integrity of the electrode and electrolyte, and how this was controlled or mitigated.*

**Authors' response:**

To mitigate beam damage, the X-ray energy is selected to 29.44 eV (decreasing absorption compared to lower energy) and the photon flux on the sample was decreased using filters and slits. With these settings, no bubble formation was observed during the experiment, indicating that gas formation through beam induced electrolysis is avoided. Voltage perturbations in the OCV are often observed if cells experience beam induced changes (reference [37] in the revised manuscript). We tracked the OCV during tomography scans (Fig. S23) and a stable OCV, apart from the expected relaxation of the concentration overpotential after interrupting the deposition, was observed indicating negligible X-ray exposure effects.

In addition, using *ex situ* SEM (figure S5), the morphology of lithium deposits can be confirmed to be qualitatively the same as during the tomography measurement. This confirms that any influence of the beam on the resulting morphology is minor.

We have clarified the strategies to mitigate beam damage and the comparison to *ex situ* SEM results in the main text on page 14 and in the supporting information on page 33.

**References:**

[37] Vanpeene V., Stamati O., Guilloud C., *et al.* Comparative Study of the Quantitative Analysis of Battery Materials with X-ray Nano-tomography: From Ex Situ toward Operando Measurements. ACS Nano 2025, 19, 10, 9994–10012

**Manuscript edits on page 14:**

Measurements were carried out at the ID16B beamline at the synchrotron facility ESRF in Grenoble, France.[38] To mitigate beam damage, the experiment was carried out with a reduced beam flux of 3.4 $10^{11}$ ph/s, using 3.5 mm of Si filters and diminished aperture opening of a horizontal slit. The sample was illuminated with a coherent conical pink beam[39] of energy 29.45 keV ($\Delta E/E \sim 10^{-2}$), produced by focusing the beam upstream of the sample (50 × 50 nm) using Kirkpatrick–Baez (KB) mirrors. Projections were acquired using a PCO Edge 4.2 CMOS camera (2048×2048 pixels), equipped with a 30 µm LSO scintillator. For a single tomogram, 2505 projections were taken with 50 ms integration time for each projection. For holo-tomography, four different sample-detector distances are used resulting in a total beam exposure time of 501 s for one measurement. Using these settings, no bubble formation, which would indicate beam

induced electrolysis, could be detected during the experiment. Further, voltage perturbations during beam exposure can be a sign of beam damage,[37] but here no voltage changes could be observed during tomography scans (**Fig. S23**) apart from the expected relaxation of the concentration overpotential. Complementary *ex situ* SEM measurements (Fig. S5) also showed good agreement with the lithium morphologies observed during the tomography experiment, suggesting that any influence of the X-ray beam on the resulting morphology is minor.

**Manuscript edits in supporting information on page 33:**

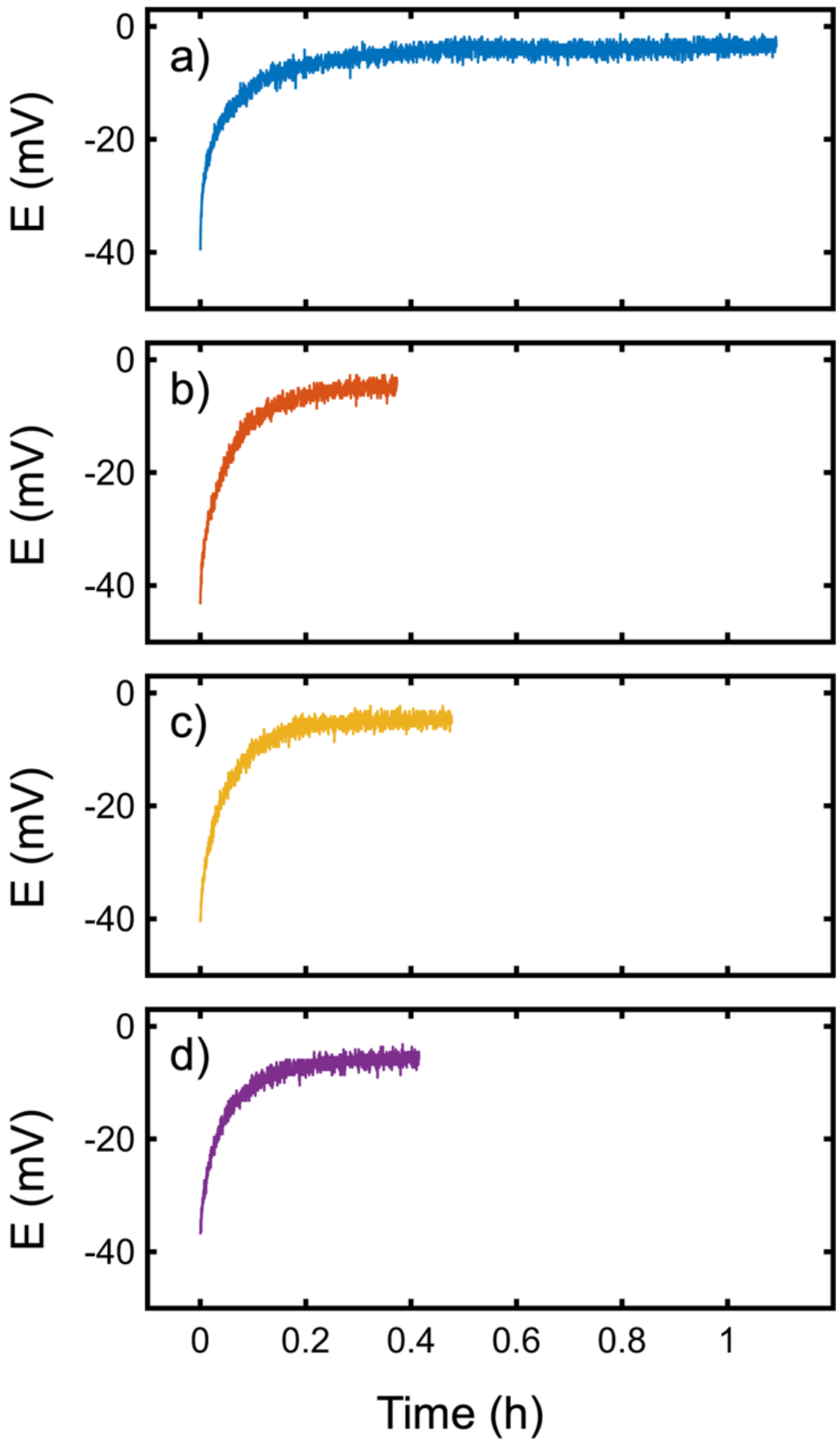


**Fig. S23**. Open circuit voltage during interruptions in Li deposition, where tomography measurements were carried out, after depositing at 1 mA/cm$^2$ for 6 min (a), 12 min (b) 24 min (c) and 60 min (d).

**<u>Response to Reviewer #2:</u>**

*Rizell et al. determined 3D structures of electrodeposited lithium as a function of time. Recently, intensive research dedicated to lithium metal batteries with solid electrolyte has shown that they are prone to metallic electrical shorts as well. Therefore, research*

*on dendrites in liquids as done by Rizell et al. is a timely one. Due to the high resolution of the images obtained for large (> (0.1 mm)3) or huge (> (0.5 mm)3) volumes, Rizell et al. obtain useful original time-dependent data with unprecedented quality. However, the manuscript needs to be improved before it can be published:*

*According to both abstract and conclusions, variations in the local current densities j_loc are linked to structural variations. However, it seems that no attempt has been made to substantiate this claim by estimating j_loc values. j_loc calculations are complicated by the ongoing change of the surface area and by the difficulty to decide which regions should be considered electroactive. Anyway, estimating j_loc ranges seems a pertinent analysis step. Since central aspects of wording and interpretation are incomplete, a major revision is required.*

**<u>Authors' response:</u>**

We thank the reviewer for the constructive feedback which we have addressed point by point below.

*In the battery community, the definition of "anode material" and "cathode material" is based on the processes during discharge. The traditional and official meaning of "anode" (as defined in the IUPAC Compendium of Chemical Terminology, see <u>https://goldbook.iupac.org/terms/view/A00370</u>) is anode = electrode at which oxidation takes place. This leads to a conflict if processes during charging are considered as done here: electrodeposition is done by reduction, i.e. at a cathode. This is not compatible with the wording used in the manuscript, e.g. in the abstract: "plated onto the anode". Please check the manuscript and avoid or resolve this conflict at all relevant places. This can be done e.g. by using "negative electrode" instead of "anode" or "cathode", or by precise wording, e.g. by describing lithium as "anode during discharge"; during charging, the cathodic process of electrodeposition takes place etc.*

**<u>Authors' response:</u>** We have removed the inconsistencies in terminology throughout the manuscript.

**Manuscript edits on page 1:**

**To pave way for application of Li-metal electrodes in high energy density batteries the evolution of the 3D structures formed during charging needs accurate characterization inside working cells.**

**Manuscript edits on page 1:**

As a result of the high capacity (3860 mAh/g) and low redox potential (–2.79 V vs SHE in propylene carbonate)[1] of Li, cells with energy densities well above those of conventional lithium-ion batteries can be realized by replacing the graphite electrode with Li metal.[2] In a Li-metal cell, lithium is electrochemically plated onto the negative electrode during charge and stripped during discharge.

**Manuscript edits on page 1:**

At low potentials electrolyte components are reduced at the negative electrode until a passivating solid electrolyte interphase (SEI) layer forms, which the Li-ions must be transported through to reach the electrode.

*p. 4.: "The degree of crystallinity of the deposited lithium was reported to depend on the current density, with more amorphous lithium forming during deposition at low current density (0.1 mA/cm2).31*
*This result reported by Wang et al. (Ref. 31) seems unexpected if one assumes that crystallization might take time. Since Wang et al state "the cluster size is key", their result might be interpreted as: larger currents lead to larger clusters, and therefore, more crystallinity. This seems to be at odds with your results: the sheets, e.g. as shown in Fig. S4 b), seem much larger than the needles shown in Fig. S4 c). If larger clusters are more crystalline, shouldn't we expect then that the sheets are crystalline?*

**Authors' response:** Wang *et al.* [1] also observe sheets that are several microns in size and attribute that to growth of glassy nuclei. Since they propose that the size of the particles/nuclei are a few nm when the disorder-order transition occurs, this suggests that after nucleation, amorphous clusters can grow without crystallizing. Therefore, larger lithium structures need not be (more) crystalline. Wang *et al.* emphasize that the "initial nucleation shapes the subsequent growth of the Li metal deposits". To reflect this, we have highlighted the role of the initial growth or nucleation of a lithium grain/structure on page 4 in the revised manuscript. In addition, one can note that sheets are not necessarily "larger", they have very similar local thickness as needles.

**References:**
[1] Wang, X. *et al.* Glassy Li metal anode for high-performance rechargeable Li batteries. *Nat. Mater.* **19**, 1339–1345 (2020).

**Manuscript edits on page 4:**
At lower current densities, more amorphous nuclei are suggested to form, which then evolve into sheet-like structures with low crystallinity.[31] Considering the proposed dependence of deposit shape on the crystallinity of the initial structures, the coexistence of needle like and sheet like lithium structures (**Fig. 1**) could be a signature of local variations in how new lithium grains form. This may result from local current density variations caused by SEI inhomogeneity and its evolution.

*By using different mean (=macroscopic) current densities, Wang at al showed clearly (their Supplementary Fig. 8) that larger currents lead to more crystallinity. Why do you think that this result of different sets of experimental conditions (currents, potentials) can be interpreted as "higher _local_ current densities result in more crystallinity in an experiment where the potential is the same at all places"? Is this a result of Wang et al. or is this your interpretation of Wang's data? Are differences in SEI thicknesses the reason for differences in local current density? Maybe it is not the local current that dictates the crystallinity, maybe it is just the other way round: Maybe high crystallinity enables faster deposition, i.e. crystallinity which might suck up mobile adatoms might be the cause of larger local currents? A proper discussion of this issue is important in the manuscript, since the corresponding sentence in the conclusions on p. 10, "The coexistence of the two types of structures points to inhomogeneities in the local current density as sheet-like structures are expected to form at lower current density compared to need-like growth" is placed as a central part of the results. Furthermore, more quantitative results are required here. Since volume changes of the parts are known (Fig. 4) and their surface areas should be accessible as well, estimating local current densities is possible. In the simplest case of needle-like growth at constant diameter, the local current density at the tip is obtained by the formula*
*v = j F / Vm (2) (p. 495) of*

*J. L. Barton and J. O'M. Bockris, Proceedings of the Royal Society of London, Series A, Mathematical and Physical Sciences, Vol. 268, No. 1335 (Aug. 7, 1962), pp. 485-505.*
*Barton and Bockris studied silver deposition where it can be assumed that the silver (m.p. 1234.93 K) atoms are not very mobile. In the case of lithium, due to its high analogous temperature (m.p. 453.65 K) lithium atoms are expected to be mobile at room temperature. This means that atoms do not need to be deposited at the same place where volume growth take place, and an exact conversion of lithium volume changes to local current densities j is impossible. However, still estimated (probably upper) values of j can be given by assuming a limited range of lithium atom diffusion. These local current densities $j = Q/(t A)$ can be estimated from the surface areas A considered to be active (e.g. those shown in red in Fig. 6), Faraday's law, $Q = m z F / M$, with $m = \rho V$, i.e. $j = \rho V z F / (t A M)$. It is clear that it is unknown which part of the surface is active. However, two special cases might be considered: a) all of the surface of the structure is active, b) an area of the order of the cross-section e.g. of the structure of Fig. 4, is active. (b) corresponds to the approach of Barton and Bockris.) Although the current densities calculated might strongly depend on the assumptions made, it is still interesting to know whether there are differences between sheets and needles if the same assumptions are made. In the case that these calculations do not give a conclusive result, they still should be reported in the supplement.*

**Authors' response:**
Variation in local current density can occur as a result of variations in the ion-concentration in the electrolyte and/or the resistance of the SEI. To shed more light on this issue we have added results from electrochemical simulations to the manuscript on page 6 (discussion and new Fig. 4). These results show (Fig. 4) that ion-concentration effects only account for minor variations of the local current density. Therefore, the interface resistance, which can vary through SEI composition and/or thickness differences/changes (as suggested by the referee), is the probable cause of local current density variations. A local current density drop over time is also to be expected since the electrode surface area is constantly increasing. Therefore, local current densities can vary in both space and time.

Estimations of local current densities, based on the suggestion by the referee, for different structures have also been added (supporting information Fig. S21). These show that the current density varies considerably for the same part of the structure during different times. However, we note that the largest local current density for any of these segments is significantly larger than the smallest current for the other segments. This overlap suggests that growth rate alone does not dictate morphology. Based on this, and in line with what Wang *et al.* [1] argue, we propose that the initial growth event is crucial for determining the final morphology. We have revised the conclusions on page 10 to reflect this emphasis on the initial growth.

Wang propose a correlation between the crystallinity of the nuclei (initial growth structure) and the final morphology. With this mechanism in mind a sheet would arise after a slow nucleation event, while not excluding that after the initial growth, the sheet could grow more quickly than, or at the same rate as, the needle. The increased crystallinity at high macroscopic current densities would then be rationalized by differences in the nucleation process. On pages 4 and 8-9 in the revised manuscript we have modified the discussion to clarify this mechanism, added the results from (page 6 in the manuscript), and the description of (supporting information page 17), the

electrochemical simulations and also added the description on calculation of local current density (supporting information pages 29-30 and figure S21).

**References:**

[1] Wang, X. *et al.* Glassy Li metal anode for high-performance rechargeable Li batteries. *Nat. Mater.* **19**, 1339–1345 (2020).

**Manuscript edits on page 4:**

At lower current densities, more amorphous nuclei are suggested to form, which then evolve into sheet-like structures with low crystallinity.[31] Considering the proposed dependence of deposit shape on the crystallinity of the initial structures, the coexistence of needle like and sheet like lithium structures (**Fig. 1**) could be a signature of local variations in how new lithium grains form. This may result from local current density variations caused by SEI inhomogeneity and its evolution.

**Manuscript edits on page 6:**

To elucidate the growth mechanism, the equilibrium Li-ion concentration and local current densities were obtained from electrochemical simulations based on the actual 3D-structures obtained from tomography. (**Fig. 4, Fig. S11-S13, Supplementary Note 7**). Assuming a homogeneous interfacial resistance across the electrode, a Li ion concentration gradient from the top of the sample to the bulk electrode is predicted, with smooth variations and no significant changes around edges or endpoints (Fig. 4a, c). This ion concentration gradient would cause slightly higher Butler-Volmer exchange current density at the structures extending the furthest into the electrolyte (Fig. 4b, d), but generally the variations are small, less than 10 %. Clearly, neither the differences between needles and sheets or the emergence of either shape, can be due to enhancement of electrical fields at tips or Li concentration gradients. Instead, effects such as crystal anisotropy and differences in interfacial resistance across the electrode, not accounted for in the simulation, must be driving the evolution of the electrode morphology.

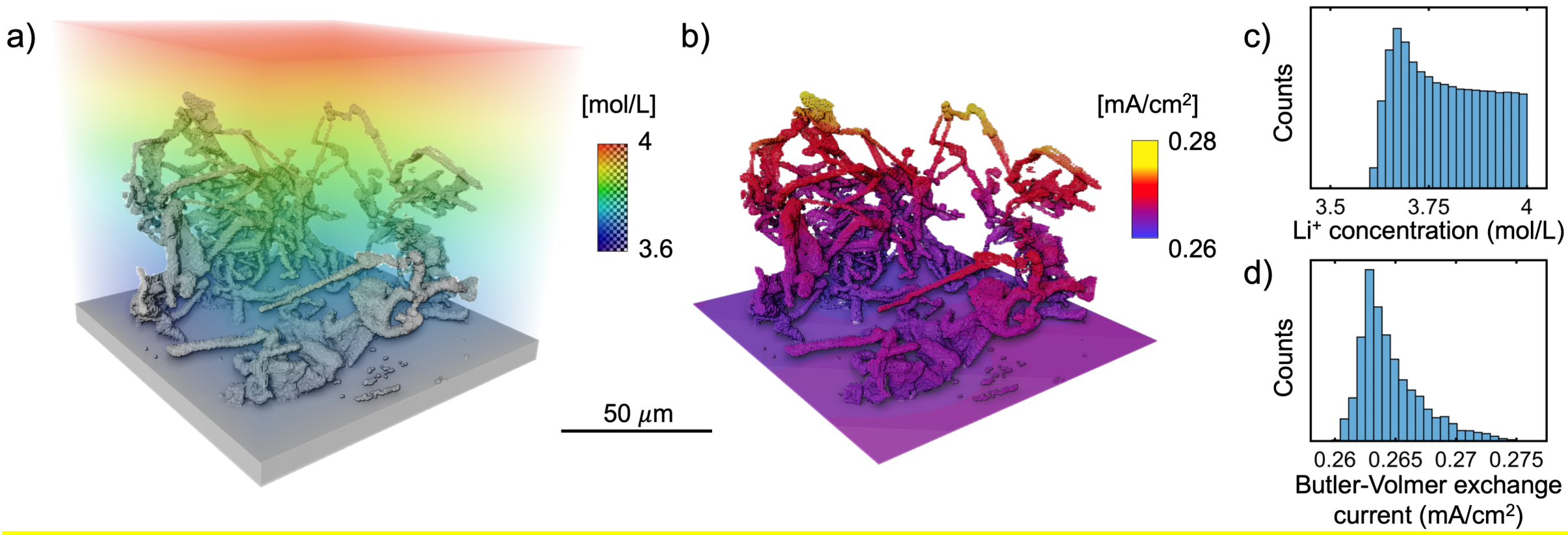


**Fig. 4**. 3D electrochemical simulations, based on Li morphology after 24 min. a) Li-ion distribution in the electrolyte, visualized with a semitransparent colormap. Grey structures correspond to the Li metal. b) Local Butler-Volmer exchange current density distribution across the electrode surface. c-d) Histograms based on the data in a-b.

**Manuscript edits on page 8-9:**

The converse transition of growth mode, from sheet to needle, can also be observed (see **Fig. S20**), however no instances of sheets growing directly from the bulk electrode could be found. The range of local current densities estimated for growing sheets and needles show significant overlap (**Fig. S21, Supplementary Note 9**), suggesting that local current density during the growth phase does not determine the morphology. Instead, the initial growth of new grains/branches could dictate which morphology evolves during

their subsequent growth. To trigger different types of growth, changes/cracks in the SEI-layer could impact the nucleation of new growth sites. This electrolyte forms a roughly 10 nm thick, amorphous SEI,[35] containing several inorganic components, such as LiF, $Li_2S$, $Li_2O$ and $Li_3N$, as determined by X-ray photoelectron spectroscopy experiments (**Fig. S22, Supplementary Note 10**). This type of interphase can be expected to be brittle and thereby prone to crack due to stress buildup during growth.[36]

## Manuscript edits on page 10:

Transitions between sheet and needle morphologies can occur within one connected structure, and it is hypothesized that transitions are caused by changes/cracks in the SEI-layer impacting the initial growth of new lithium grains, but more work is required to fully understand the mechanism.

## Manuscript edits in supporting information page 29-30:

**Supplementary Note 9: Local current density estimate**

The local current density, $j$, in a region of the electrode can be calculated as

$$j = \frac{Q}{tA},$$

Where, $A$, is the area, $t$, is the time, and $Q$ is the charge passed to the electrode. Using Faraday's law, the expression can be rewritten as

$$j = \frac{\rho V n F}{tAM}.$$

Where $\rho$ is the density of lithium, $V$ is the volume of lithium deposited in the time $t$, $n$ is the number of electrons transferred in the reaction (1), $F$ is Faraday's constant, $M$ is the molar mass of lithium. With tomography images at two different times, $t_1$ and $t_2$, this formula can be used to estimate time-averaged local current density values at $t=(t_1+t_2)/2$ by determining the lithium volume increases, $V= V_2-V_1$, and average lithium surface areas $A=(A_1+A_2)/2$. **Fig. S21** shows this calculation for different segments of the lithium structure from Fig. 6. Calculations under two different assumptions are shown:

1. All of the surface area is active in the deposition, but lithium is redistributed to the growth zones through diffusion (Fig. S21a)
2. The lithium surface diffusion on the structures can be neglected, which implies that the lithium must be deposited in the growth zones allowing the current density to be estimated based on the surface areas of the growth zones. For the needle (segment 1) active areas at both ends of the needle are assumed, with a cross-sectional area of roughly 3.9 $\mu m^2$, a total active area of A=7.8 $\mu m^2$ is used for the calculation. For the sheets (segment 3) the growth occurs along its edges, the surface of which can be manually defined in the tomography volumes (red in Fig. S21b).

Neither of these cases are likely to be fully representative of the deposition process but enable straightforward calculations of the local current density. For both cases, the current density shows a quick decline over time. Given that the deposition current is constant, it is inevitable that the current densities decrease when the surface area of the electrode increases through inhomogeneous deposition. This trend should also be exacerbated by the gradually increasing thickness/resistivity of the SEI as the structures age.

The largest local current density for any of these segments is significantly larger than the smallest current for the other segments. This could suggest that the local current density during the growth does not determine the morphology. A possible explanation for the vastly different morphologies could be that different nucleation events dictates which morphology evolves during subsequent growth, as suggested by Wang *et al*.[17]

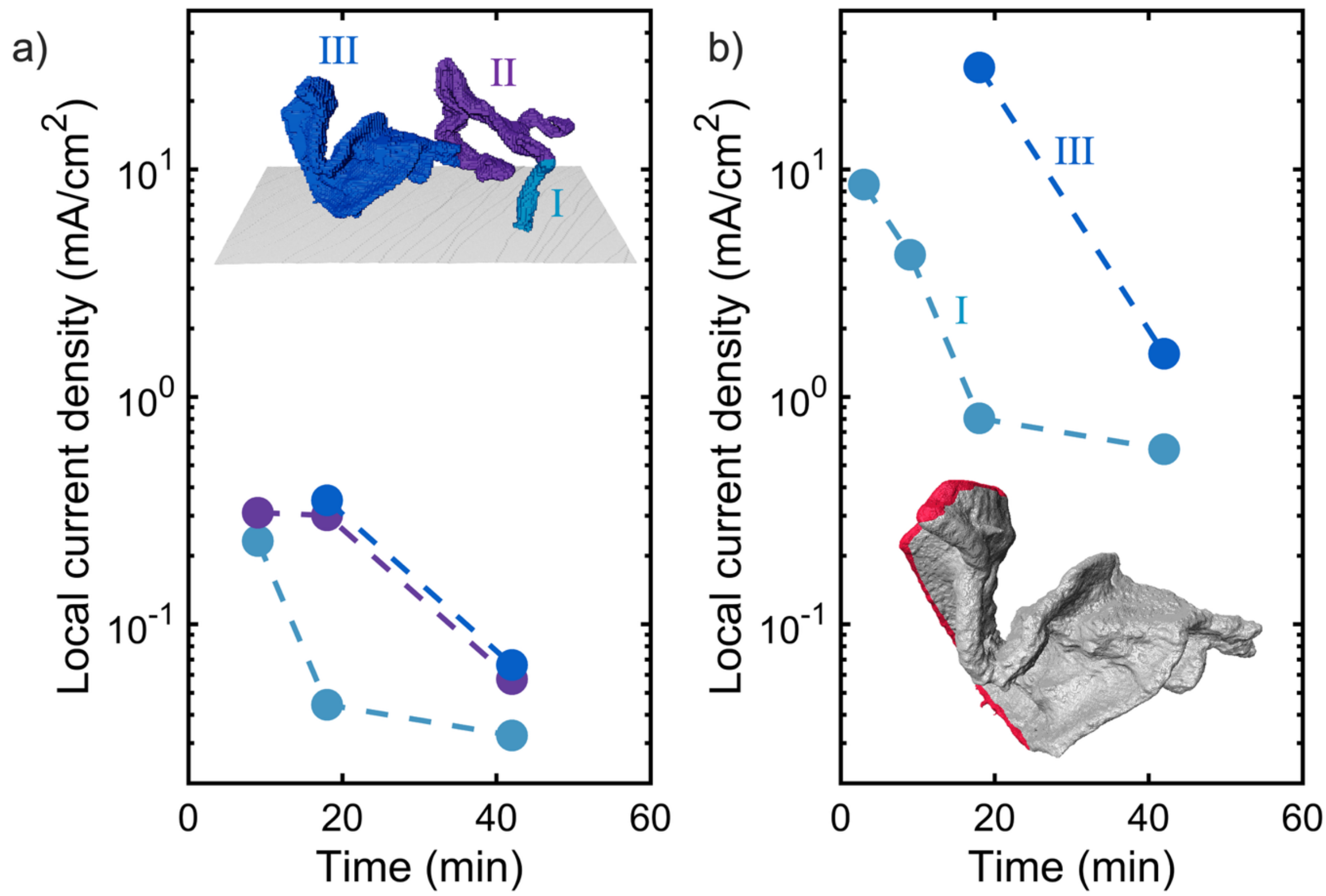


**Fig. S21**. Current density estimations. a) Current densities estimated for segment I, II and III of the structure (see inset), assuming their entire surface area is active during deposition. b) Current densities for segment I and III, calculated using assumptions regarding which areas are active during the deposition. Inset shows active area defined for segment 3 after 60 min of deposition.

*p. 9 – 10, Fig. 6:*
*"As the deposit grows, stress will be exerted on the SEI-layer which can crack, exposing fresh Li, triggering growth of new branches (Fig. 6a)."*
*This text is a good description for 6 b, but it is not convincing for 6 a.*
*In Fig. 6 b, there is a growth zone around the sheet. Therefore, due to the growing lithium, it is clear that the SEI layer can be stressed, stretched and cracked, resulting in the growth mode as indicated. However, in Fig. 6 a), a SEI crack is shown on the side of a needle. Since there is no growth zone there, there is no reason for the SEI to crack – it should rather grow there. Could you please check in the original images that resulted in the schematic of Fig. 6 whether a surrounding needle touching the observed one might have caused the SEI to crack? If there is no such external cause of an SEI damage, an internal cause is required, e.g. a groove or a mound that results in a modified SEI or extra Li deposition. Fig. S 13 a) shows an example of a growing branch; since there is no image without the branch, the result is compatible with the view that the nucleation of the branch might have occurred already during the formation of the segment where it grows later. In Fig. 4 there is also a branching. Is is compatible with the view that the branch occurs on a mound that is present early? On a short needle segment, Li atoms deposited onto it might diffuse to growth zones nearby. If the needle gets longer, the distance to the growth zones get longer. Would it be a possible mechanism that a branch nucleates by accumulation of atoms that are too far away from a growth zone?*

**Authors' response:**
We agree that the SEI should crack where the expansion occurs since the SEI is expected to be attached to the Li surface. The model in Fig. 8 has been modified to be

consistent with this reasoning. An additional figure has also been added in SI (Fig. S7) where the branching on a side of a needle is observed without any mound/groove present or any surrounding structures touching this part of the needle before the branch appeared. We hypothesize that the formation of a new branch/grain in such a location could be due to a long diffusion path to the growth zone as suggested by the reviewer, while acknowledging that branching triggered by SEI-cracking could also occur in other locations. This is discussed in the revised manuscript on page 9 in connection to the revised figure 8, and the new figure S7 is added and a note on branching in supporting information on page 32.

### Manuscript edits on page 10:

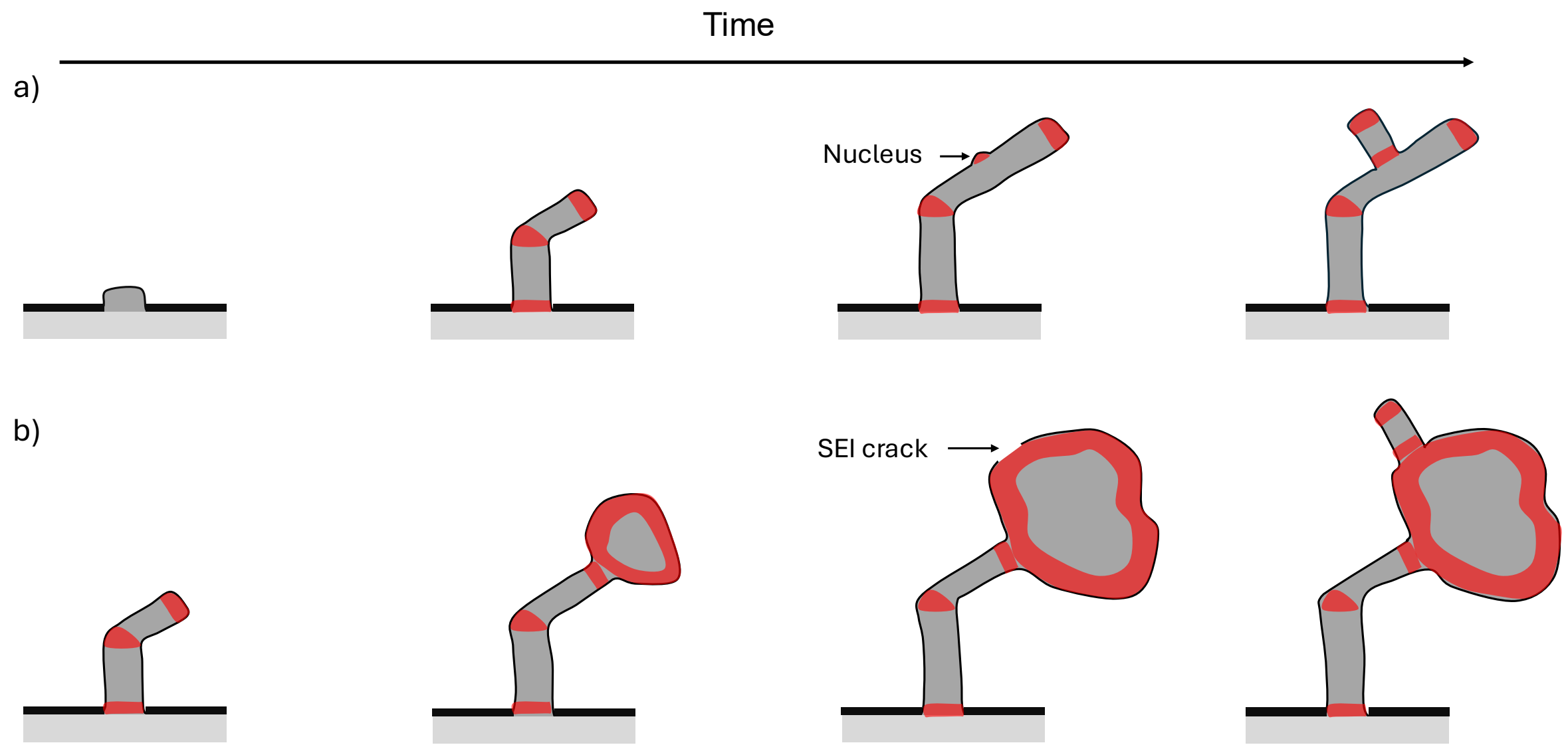


**Fig. 8**. Schematic of lithium growth modes and proposed transition mechanisms. a) Growth of a needle b) Growth of a sheet from a needle. Semi-transparent red marks growth zones in each timestep and thicker black lines show the native layer covering the bulk electrode.

### Manuscript edits on page 9:

**Fig. 8** summarizes the observed growth modes. After a deposit has pierced the native surface layer, preferential growth of this deposit is observed. Needles are shown to grow through elongation, which occurs in parallel in several segments. This can be rationalized as crystal grains grow along a specific direction(s), forming growth zones at the needle base, tip or at kinks. New branches have been observed to grow from the sides of existing needles (**Fig. S7, Fig. 8a, Supplementary Note 11**), which could be rationalized by a long diffusion path to the growth zones. Segments with sheet-like morphology are also observed (**Fig. 8b**) to grow from needles during the deposition. While the mechanism for their formation remains to be determined, they grow from the edges through in-plane expansion. As the sheet grows, stress will be exerted on the SEI-layer which can crack, exposing fresh Li, which could trigger growth of a needle from the crack.

### Manuscript edits in supporting information on page 11:

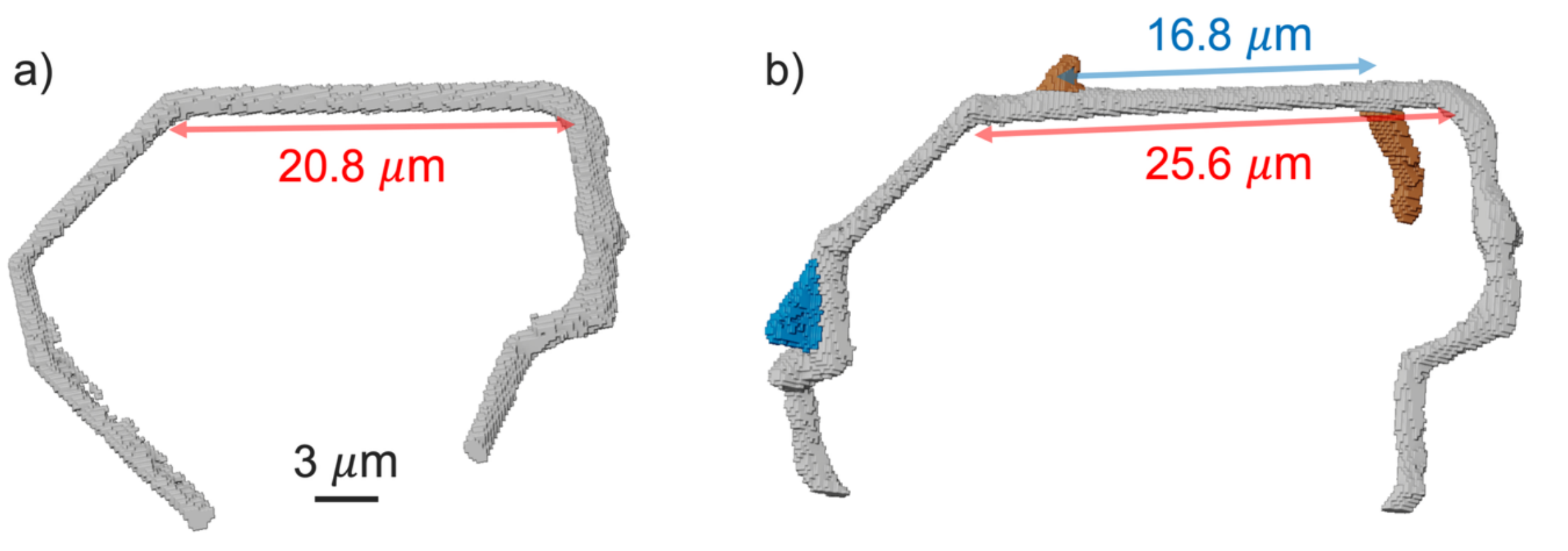


**Fig. S7**. Nucleation of new branches and elongation of a Li-needle. **a)** A needle found after 24 min of deposition. **b)** The same needle after 60 min of deposition. New needle-like branches are shown in brown and a sheet growing from the side of a segment is shown in blue.

### Manuscript edits in supporting information on page 32:

**Supplementary Note 11: Branching**

**Fig. S7** shows branches (brown) that start growing from a needle. A segment between two kinks which is 20.8 micrometers long after 24 min (Fig. S7a) has two branches (brown) 16.8 micrometers apart after 60 min of deposition (Fig. S7b). This shows that at least one of the branches must have nucleated along the segment (presumably a single crystal grain) rather than at the kink (grain boundary). If lithium tends to grow through insertion at grain boundaries at kinks,[21] nucleation of a new branch along the side of a needle could be triggered by a long diffusion path to the growth zones. Note that sheet-like morphologies also can grow from the sides of needle segments (Fig. S7b).

*p. 1, and p. 10: "need-like" ?*
*Shouldn't that read "needle-like" ?*

**Authors' response:** The first example was revised and the second sentence mentioned was removed in the revision.

### Manuscript edits on page 1:

Homogeneous deposition is expected below a mechanically strong SEI, whereas imperfections or cracks in the SEI result in hotspots for deposition with heterogeneous growth as a result, e.g. as needle-like structures.

*p. 7:*
*"... the growth rate significantly decreases in the later stages of deposition for all structures despite an increasing surface area, which can be linked to the formation of a thicker more resistive SEI."*
*As you explain correctly, due to the OCV periods during tomography, it is expected that the SEI thickness increase is pronounced and inevitable. However, even for constant current conditions without OCV periods, where fresh surfaces are created continously, a decrease in the growth rate can be observed, see Fig. 1 of Becherer et al., "Similarities in Lithium Growth at Vastly Different Rates", ChemElectroChem 2021, 8, 3882 – 3893. Fig. S14 does not only demonstrate a growing impedance during OCV, it also shows a decreasing total resistance during deposition, most likely due to an increasing surface area which results in a decreasing current density – it gets more and more difficult for the deposition to outpace the SEI growth.*

**Authors' response:**

The increase of the surface area is indeed a possible additional explanation for the observed decline in growth rate. We have modified the wording on page 7-8 to highlight this possibility and point out that the increasing surface area will contribute to the overall reduction seen in the impedance during the deposition.

**Manuscript edits on page 7-8:**

One can note that the growth rate significantly decreases in the later stages of deposition for all structures, which can be linked to the increasing total electrode surface area, effectively reducing the average local current density, and the formation of a thicker more resistive SEI on aging parts of the structure, redirecting deposition towards freshly formed structures. This explanation is supported by electrochemical impedance spectroscopy (EIS) measurements (**Fig. S18**). During deposition, when more fresh structures are continuously formed, the overall electrode impedance decreases as the electrode surface area increases. However, the electrode impedance increases when held at OCV after deposition, highlighting that the SEI becomes more resistive as it ages.

*p. 13: "pink beam": since this term might be unfamiliar, a reference might be helpful, e.g. to Meents et al. 2017, https://doi.org/10.1038/s41467-017-01417-3.*

**Authors' response:**

We have added a reference (ref 39) on page 14 to help readers unfamiliar with the terminology.

**Manuscript edits on page 14:**

The sample was illuminated with a coherent conical pink beam[39] of energy 29.45 keV ($\Delta E/E \sim 10^{-2}$), produced by focusing the beam upstream of the sample (50 × 50 nm) using Kirkpatrick–Baez (KB) mirrors.

[39] Meents, A. *et al.* Pink-beam serial crystallography. *Nat. Commun*. **8**, 1281 (2017).

*Just a remark: Constructing a good electrochemical cell for lithium is difficult. Neither PEEK nor glue are expected to be stable at lithium potential – also PTFE reacts with Li.*

**Authors' response:**

We agree that it is both challenging and important to design an effective cell for Li and have ongoing efforts in the lab to improve the cell design for future works. From our experience, PEEK provides the best compromise of (electro)chemical stability, X-ray transmission and machinability compared to other candidate materials such as glass, or various polymers. Additionally, we agree that glue should be avoided, but in the present case UV-polymerizing glue was used as a secondary safety sealing strategy at the cell's tubing ends, located far away from its electrochemically active regions, and therefore no significant impact on the electrochemistry is expected from the glue.

*Fig. S16. "Needle (orange)"*
*Please check the colors. On my screen, there is no orange needle. There is one brown structure in the left part of S16 and two brown structures in the right panel of S16. Please label as a) and b).*
*"Connected Li structure not of interest are shown in grey". In the left panel, the grey structure does not seem connected, and it seems to be too narrow to be realistic. Is this an artifact which might be removed?*

**Authors' response:**

We have changed the description of the color to brown instead of orange and labelled the left and right panels as a) and b) in this figure (Fig. S20 in the revised manuscript). The grey structure in the left figure is an artefact from visualizing the bulk electrode. The bulk electrode has been cropped in the visualization to avoid this confusion.

**Manuscript edits on supporting information page 28:**

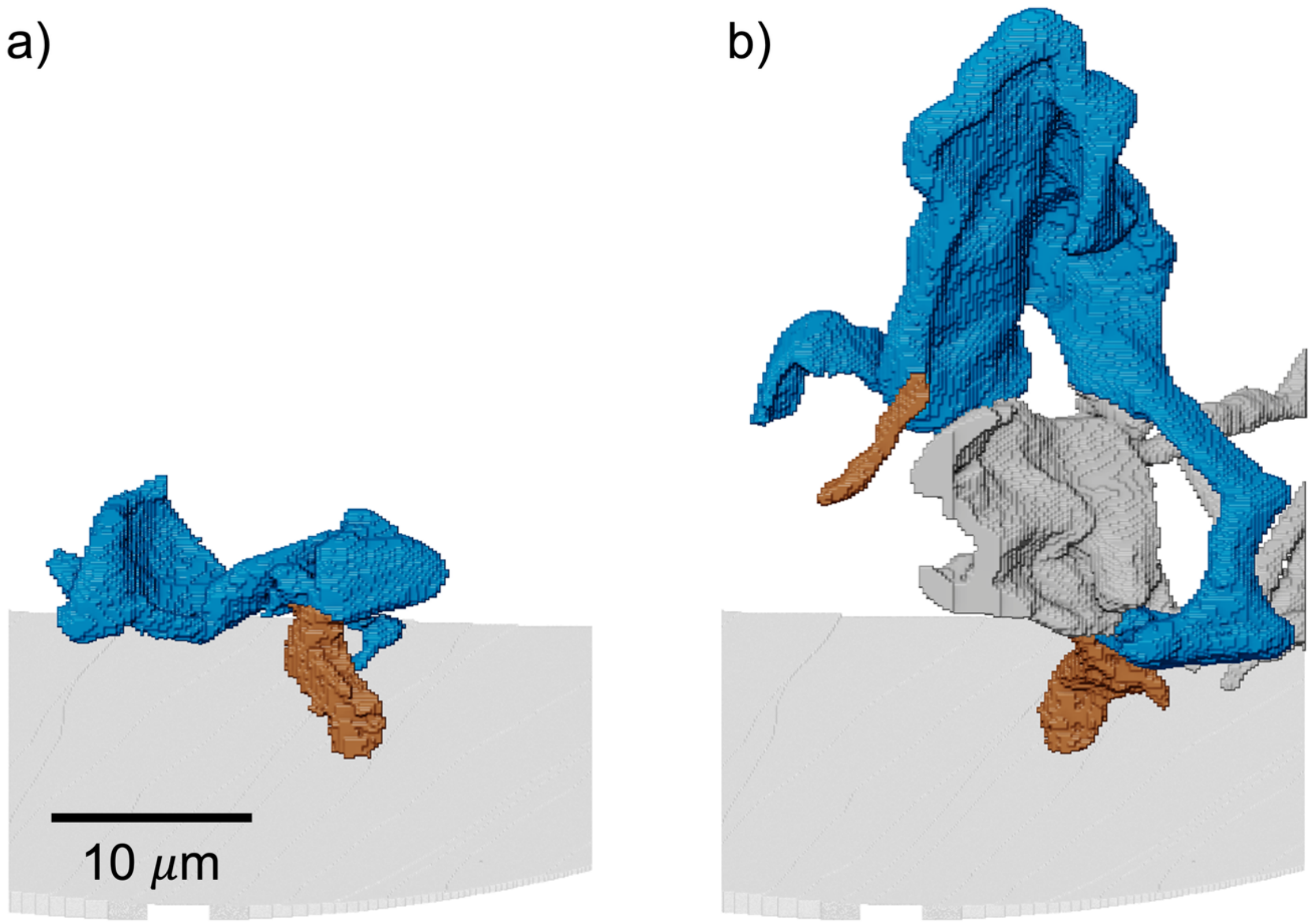


**Fig. S20**. Needle (brown) growing from a sheet (blue), between 12 (a) and 24 min (b) of deposition. Connected Li structure not of interest are shown in grey.

## Response to Reviewer #3:

*In this manuscript, the authors tracked the evolution of Li deposition utilizing X-ray nano holotomography. Due to the difference in real and imaginary part of the refractive index between the electrolyte and Li metal, the morphology and the evolution can be visualized. Dendritic and sheet-like Li deposits were observed and tracked with lithiation.*

*However, similar dendritic and sheet-like deposits have been observed and 3D visualized using cryo-FIB (Nature 2018, 560, 345–349). The morphological evolution of Li deposition with a sub-micrometer resolution has also been reported (Nat Commun 14, 854 (2023)). While this paper does provide novel nano-holoCT data with Li propagation at the nanometer scale presented, it doesn't really add much impact or enlighten the community with important findings. More effort could be paid to data analysis and the mechanism. Therefore, the reviewer is not convinced that this manuscript could meet the standard and should be considered for publication in Nat. Commun.*

**Authors' response:**

We recognize the value of the referenced studies, but our work provides *in situ* 3D imaging during lithium deposition with unprecedented spatial resolution. Lower-resolution tomography (note that the resolution in e.g. *Nat Commun 14, 854 (2023)* is around 1-2 µm compared to 150 nm in our study), cannot capture thin lithium branches or detailed connectivity. This highlights the importance of our approach for tracking deposition on branch or deposit level and understanding how/from where lithium has

grown. In contrast to cryo-FIB and STEM imaging (*Nature 2018, 560, 345–349*), which are limited to small (a few $\mu$m wide), destructively analyzed regions, our method enables observation of structural evolution and connectivity with a large field of view (>100 $\mu$m) and *in situ*.

Although the sheet structure observed in (*Nature 2018, 560, 345–349*) is similar to what we observe, the paper distinguishes the two different types of lithium deposit claiming that one is a LiH structure. Leveraging our data on structural evolution, we can conclude that this is unlikely in our case as LiH is poorly electronically conductive and would not permit continued electrodeposition, which we observe. This clearly demonstrates the utility of *in situ* high resolution 3D imaging to accurately and in detail describing the deposition mechanism, where we provide a drastically different explanation compared to e.g. *Nature 2018, 560, 345–349*.

With the improved resolution and capability of *in situ* measurements with a large field of view we can therefore track the detailed evolution of structures and determine growth modes. Key findings from our data include that i) the local thickness of growing lithium structures remains nearly constant and ii) we can isolate and analyze individual branches, revealing that multiple segments grow in parallel, with freshly deposited structures growing faster. To further elucidate the growth mechanisms additional data analysis, on for instance the local current density on individual deposits, has been added as well as electrochemical simulations based on the actual 3D data from tomography to investigate the role of Li-ion concentration and local current density. Discussions and additional analysis are found on pages 6 and 10 (new figures 4, S7 and S21). The added simulations highlights that the type of novel high-resolution 3D data provided here can be used as a base for simulations of electrodeposition. We have also revised the conclusions on page 10 to better highlight the novelty and impact of our studies.

**Manuscript edits on page 6 regarding the electrochemical modelling:**
To elucidate the growth mechanism, the equilibrium Li-ion concentration and local current densities were obtained from electrochemical simulations based on the actual 3D-structures obtained from tomography. (**Fig. 4, Fig. S11-S13, Supplementary Note 7**). Assuming a homogeneous interfacial resistance across the electrode, a Li ion concentration gradient from the top of the sample to the bulk electrode is predicted, with smooth variations and no significant changes around edges or endpoints (Fig. 4a, c). This ion concentration gradient would cause slightly higher Butler-Volmer exchange current density at the structures extending the furthest into the electrolyte (Fig. 4b, d), but generally the variations are small, less than 10 %. Clearly, neither the differences between needles and sheets or the emergence of either shape, can be due to enhancement of electrical fields at tips or Li concentration gradients. Instead, effects such as crystal anisotropy and differences in interfacial resistance across the electrode, not accounted for in the simulation, must be driving the evolution of the electrode morphology.

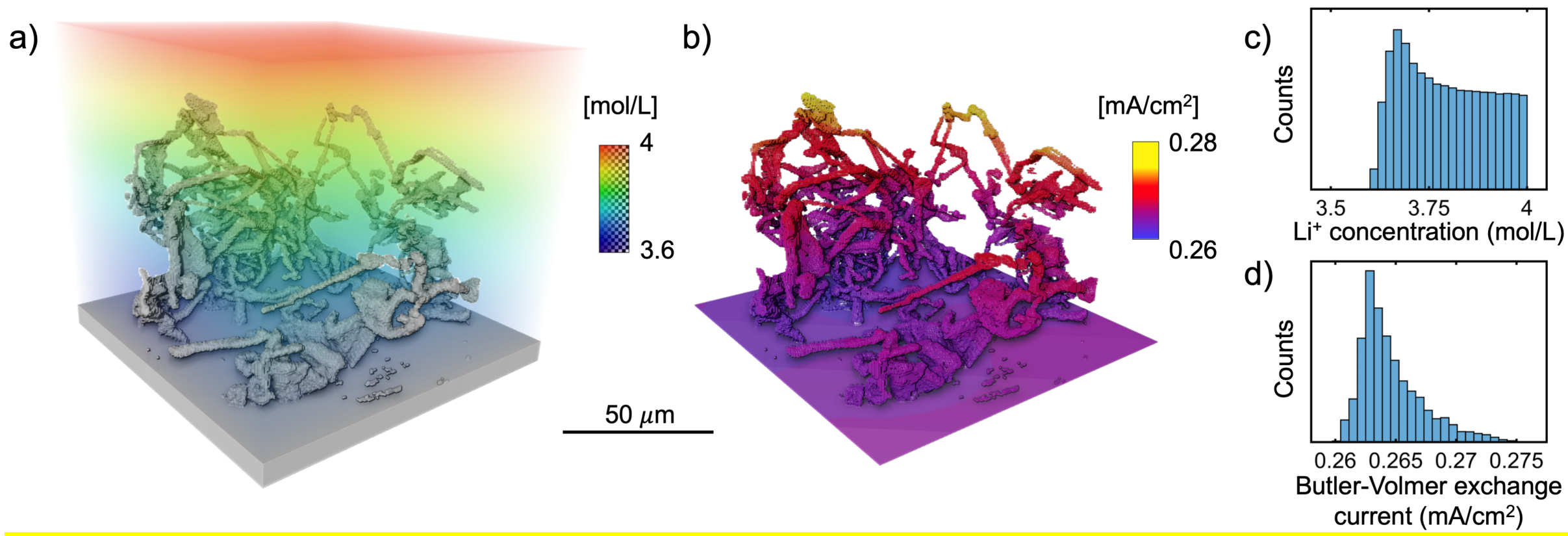


**Fig. 4**. 3D electrochemical simulations, based on Li morphology after 24 min. a) Li-ion distribution in the electrolyte, visualized with a semitransparent colormap. Grey structures correspond to the Li metal. b) Local Butler-Volmer exchange current density distribution across the electrode surface. c-d) Histograms based on the data in a-b.

### Manuscript edits on page 10:

We present the first time-resolved analysis of lithium metal electrodeposition in 3D with a sub-micron spatial resolution, highlighting the potential of *in situ* X-ray nano-tomography to elucidate the growth modes and formation mechanisms of Li structures. Lithium electrodeposition is captured at multiple scales, ranging from entangled lithium structures, down to (sub)micron-sized segments within individual lithium deposits. Two fundamentally different Li structures coexist and build up the mossy Li morphology, (i) one-dimensional needle-like lithium, presumed to be single crystalline and (ii) two-dimensional wrinkled, amorphous or polycrystalline, sheets. Based on our data, we propose a model for Li growth, where Li structures also grow with almost constant thickness, through branching, elongation of existing branches and planar growth of sheets. In a single connected structure, different segments grow simultaneously, however freshly deposited parts grow faster, pointing to a gradually increasing resistance of the SEI over time. Transitions between sheet and needle morphologies can occur within one connected structure, and it is hypothesized that transitions are caused by changes/cracks in the SEI-layer impacting the initial growth of new lithium grains, but more work is required to fully understand the mechanism. For instance, the coupling between SEI layer properties and deposit morphology needs to be rationalized, calling for both systematic studies exploring the impact of current density and electrolyte formulation, to shed light on the role of the SEI by tuning its characteristics, as well as theoretical modelling based on 4D tomography datasets. Development of more advanced computational models, which account for e.g. the SEI layer properties and lithium crystal anisotropy, will be important to help explain how different lithium morphologies form. More broadly, *in situ* X-ray nano-tomography experiments could help shed light on a wide range of processes influencing the morphology of lithium metal electrodes such as stripping or cycling under pressure.

*1.Side reactions and beam damage both could lead to gas formation during the in-situ measurement. Is there any gas observed using X-ray imaging during the Li deposition? Four scans are conducted for each tomography measurement. How long is the total scan time? How do the authors deal with the potential beam damage?*

**Authors' response:**

The total time with beam exposure during a holo-tomography measurement is 501 s (2505 projections, with 50 ms integration time, at 4 different sample-detector distances). This has been clarified on page 14 of the manuscript.

To mitigate beam damage, the X-ray energy is selected to 29.44 eV (decreasing absorption compared to lower energy) and the photon flux on the sample is decreased using filters and slits. No bubble formation, indicating gas evolution, is observed during measurements, indicating that excessive beam induced radiolysis is mitigated. Voltage perturbations in the OCV can often be observed if cells experience beam induced changes (reference [37] in the revised manuscript), which was tracked during tomography scans (Fig. S23). A stable OCV, apart from the expected relaxation of the concentration overpotential after interrupting the deposition, was confirmed indicating acceptable X-ray exposure. Using *ex situ* SEM (figure S5), the morphology of lithium

deposits is also qualitatively confirmed. This confirms that any influence of the beam on the resulting morphology is limited. We have clarified these points on page 14 in the methods section and in supporting information (p33).

**References:**

[37] Vanpeene V., Stamati O., Guilloud C., *et al.* Comparative Study of the Quantitative Analysis of Battery Materials with X-ray Nano-tomography: From Ex Situ toward Operando Measurements. ACS Nano 2025, 19, 10, 9994–10012

**Manuscript edits on page 14:**

To mitigate beam damage, the experiment was carried out with a reduced beam flux of 3.4 $10^{11}$ ph/s, using 3.5 mm of Si filters and diminished aperture opening of a horizontal slit. The sample was illuminated with a coherent conical pink beam[39] of energy 29.45 keV ($\Delta E/E \sim 10^{-2}$), produced by focusing the beam upstream of the sample (50 × 50 nm) using Kirkpatrick–Baez (KB) mirrors. Projections were acquired using a PCO Edge 4.2 CMOS camera (2048×2048 pixels), equipped with a 30 µm LSO scintillator. For a single tomogram, 2505 projections were taken with 50 ms integration time for each projection. For holo-tomography, four different sample-detector distances are used resulting in a total beam exposure time of 501 s for one measurement. Using these settings, no bubble formation, which would indicate beam induced electrolysis, could be detected during the experiment. Further, voltage perturbations during beam exposure can be a sign of beam damage,[37] but here no voltage changes could be observed during tomography scans (**Fig. S23**) apart from the expected relaxation of the concentration overpotential. Complementary *ex situ* SEM measurements (Fig. S5) also showed good agreement with the lithium morphologies observed during the tomography experiment, suggesting that any influence of the X-ray beam on the resulting morphology is minor.

**Manuscript edits in supporting information on page 33:**

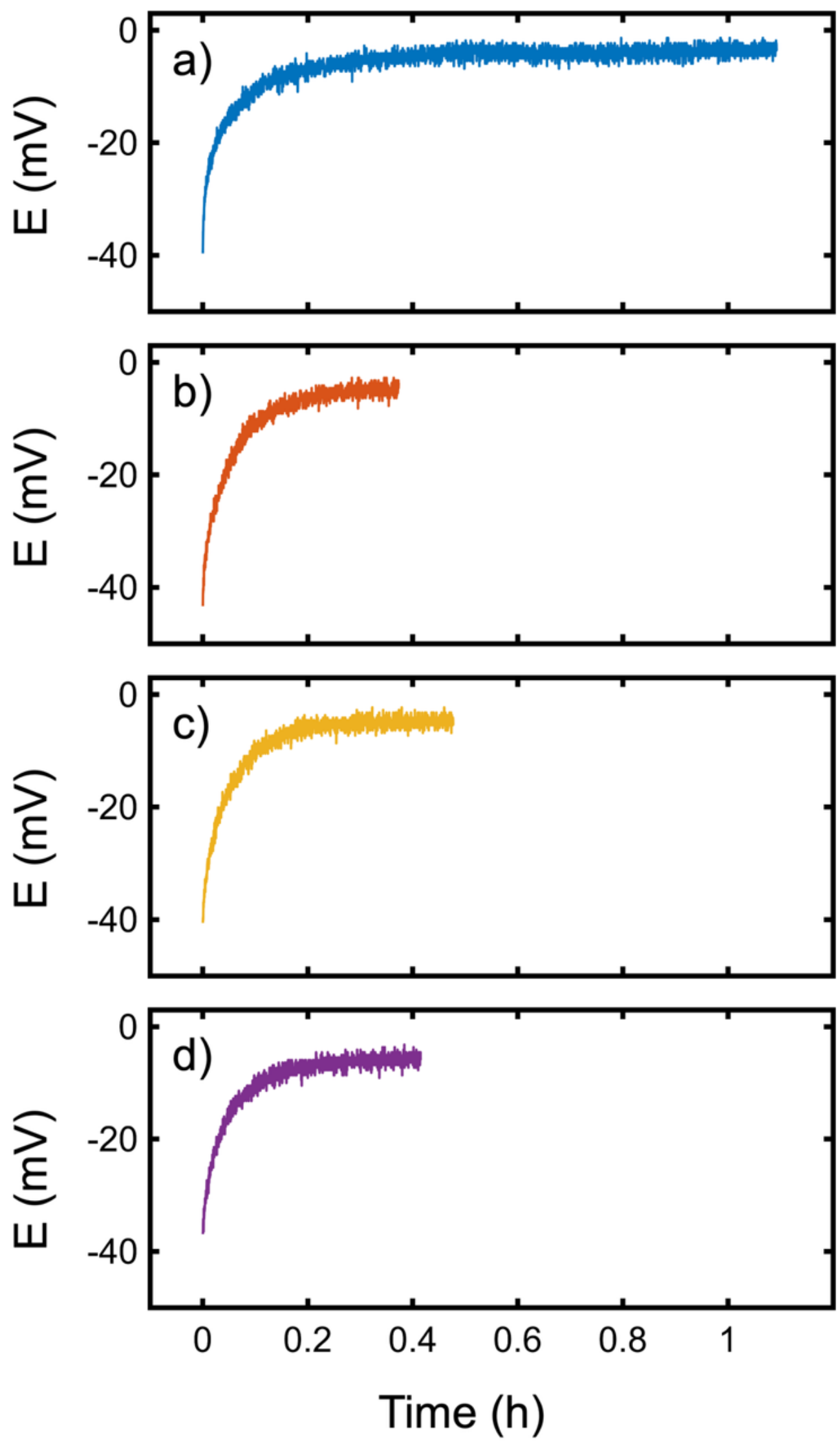


**Fig. S23**. Open circuit voltage during interruptions in Li deposition, where tomography measurements were carried out, after depositing at 1 mA/cm² for 6 min (a), 12 min (b) 24 min (c) and 60 min (d).

*2.Why a concentration of 4M LiFSI/DME electrolyte was used instead of 1 M?*

**Authors' response:** The highly concentrated, 4M, electrolyte was chosen due to excellent long term cycling performance as shown by Quian et al [1]. Additionally, as the image contrast (see Fig. S2) in holo-tomography measurements is related to the electron density difference between the studied materials [2], which means that a 4M electrolyte, due to its higher electron density than a 1M mixture, will enable better image contrast with Li. We have clarified this point on page 14 in the revised manuscript.

**References**

[1] Qian, J., Henderson, W., Xu, W. et al. High rate and stable cycling of lithium metal anode. Nat Commun 6, 6362 (2015). https://doi.org/10.1038/ncomms7362

[2] Pogany, A., Gao, D. & Wilkins, S. W. Contrast and resolution in imaging with a microfocus x-ray source. Rev Sci Instrum 68, 2774–2782 (1997).

**Manuscript edits on page 14:**

The final electrolyte was a 4M mixture, magnetically stirred at room temperature for 24h to ensure complete salt dissolution. Note that the high salt concentration helps improve the image contrast (between Li metal and electrolyte) in this experiment.

*3. What are the implications for the community from the phenomenon and results found in this work?*

**Authors' response:**

There are three main implications from this work:

(1) X-ray nano-tomography can provide 3D images with unprecedented spatial resolution, allowing e.g. the evolution of single branches of lithium structures to be tracked in 3D for the first time. This highlights new opportunities to investigate differences in growth mechanisms between electrolytes, current densities, stack pressures or even different metal anodes (e.g. sodium, zinc, and magnesium all highly relevant for next generation batteries).

(2) Detailed and quantitative insights on the lithium metal growth. For instance, we find that lithium grows with almost constant local thickness (without external pressure). Based on our results we propose that this is driven by crystal anisotropy and/or a large negative surface charge. We have added an additional argument for this based on a new figure (Fig. S7) and discussion on page 4 in the manuscript. We also demonstrate that lithium pierces the native layer in a few places and then continues to grow from those (Fig. S9), which implies that the initial structures formed have a very large effect on morphological evolution (either at the onset of the first nucleation event or during branching of Li structures). Based on our observations, we propose a new model for the growth. A good understanding of the growth mechanism could be leveraged for better strategies to control the lithium morphology.

(3) High resolution 4D datasets like ours open new possibilities for modelling of the deposition mechanism. We have added an example of this in Fig. 4, but more advanced models can also be developed, attempting to account for more of the factors influencing the morphology.

We have revised the conclusion on page 10 to highlight these aspects.

**Manuscript edits on page 10:**

We present the first time-resolved analysis of lithium metal electrodeposition in 3D with a sub-micron spatial resolution, highlighting the potential of *in situ* X-ray nano-tomography to elucidate the growth modes and formation mechanisms of Li structures. Lithium electrodeposition is captured at multiple scales, ranging from entangled lithium structures, down to (sub)micron-sized segments within individual lithium deposits. Two fundamentally different Li structures coexist and build up the mossy Li morphology, (i) one-dimensional needle-like lithium, presumed to be single crystalline and (ii) two-dimensional wrinkled, amorphous or polycrystalline, sheets. Based on our data, we propose a model for Li growth, where Li structures also grow with almost constant thickness, through branching, elongation of existing branches and planar growth of sheets. In a single connected structure, different segments grow simultaneously, however freshly deposited parts grow faster, pointing to a gradually increasing resistance of the SEI over time. Transitions between sheet and needle morphologies can occur within one connected structure, and it is hypothesized that transitions are caused by changes/cracks in the SEI-layer impacting the initial growth of new lithium grains, but more work is required to fully understand the mechanism. For instance, the coupling between SEI layer properties and deposit morphology needs to be rationalized, calling for both systematic studies exploring the impact

of current density and electrolyte formulation, to shed light on the role of the SEI by tuning its characteristics, as well as theoretical modelling based on 4D tomography datasets. Development of more advanced computational models, which account for e.g. the SEI layer properties and lithium crystal anisotropy, will be important to help explain how different lithium morphologies form. More broadly, *in situ* X-ray nano-tomography experiments could help shed light on a wide range of processes influencing the morphology of lithium metal electrodes such as stripping or cycling under pressure.

*4.In Fig 5 and 6, the mechanism of the transition from Li needle to sheet is still not clear. More analysis and explanation are suggested.*

**Authors' response:**

To shed light on this transition we have analyzed the local current densities during the growth of a Li sheet and a Li needle, establishing that there is significant overlap between their local current densities (See new supplementary Note 9, Fig. S21). Further, electrochemical simulations have been carried out (Fig. 4), establishing that the differences between sheet and needle growth cannot arise due to ion-concentration differences in the electrolyte or electric field effects. Instead, the transitions between different growth modes need to be triggered by for instance differences in interfacial resistance, which is not captured by the simulation.

Wang *et al.* [1] propose that the initial growth event/nucleation, and particularly the crystallinity of the structure, is crucial for determining the final morphology. With this mechanism in mind a sheet would arise after a slow nucleation event, while not excluding that after the initial growth, the sheet could grow more quickly than, or at the same rate as, the needle.

We have clarified these points on pages 6 and 8-9 in the revised manuscript. However, we also emphasize in the manuscript that the mechanism for the transition to sheet growth needs further investigation in the conclusion on page 10.

**References**

[1] Wang, X., Pawar, G., Li, Y. et al. Glassy Li metal anode for high-performance rechargeable Li batteries. Nat. Mater. 19, 1339–1345 (2020).

**Manuscript edits on page 6:**

To elucidate the growth mechanism, the equilibrium Li-ion concentration and local current densities were obtained from electrochemical simulations based on the actual 3D-structures obtained from tomography. (**Fig. 4, Fig. S11-S13, Supplementary Note 7**). Assuming a homogeneous interfacial resistance across the electrode, a Li ion concentration gradient from the top of the sample to the bulk electrode is predicted, with smooth variations and no significant changes around edges or endpoints (Fig. 4a, c). This ion concentration gradient would cause slightly higher Butler-Volmer exchange current density at the structures extending the furthest into the electrolyte (Fig. 4b, d), but generally the variations are small, less than 10 %. Clearly, neither the differences between needles and sheets or the emergence of either shape, can be due to enhancement of electrical fields at tips or Li concentration gradients. Instead, effects such as crystal anisotropy and differences in interfacial resistance across the electrode, not accounted for in the simulation, must be driving the evolution of the electrode morphology.

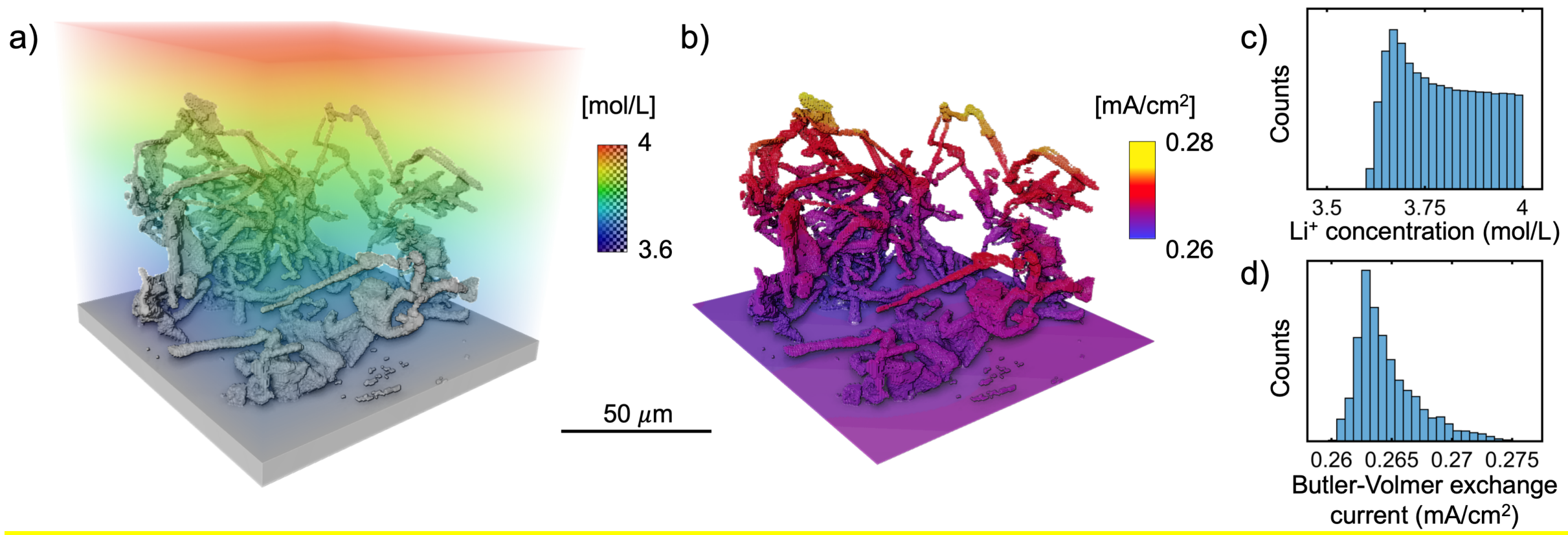


**Fig. 4**. 3D electrochemical simulations, based on Li morphology after 24 min. a) Li-ion distribution in the electrolyte, visualized with a semitransparent colormap. Grey structures correspond to the Li metal. b) Local Butler-Volmer exchange current density distribution across the electrode surface. c-d) Histograms based on the data in a-b.

## Manuscript edits on page 8-9:

Similarly, **Fig. 7b** shows a sheet formed at the tip of a needle. The converse transition of growth mode, from sheet to needle, can also be observed (see **Fig. S20**), however no instances of sheets growing directly from the bulk electrode could be found. The range of local current densities estimated for growing sheets and needles show significant overlap (**Fig. S21, Supplementary Note 9**), suggesting that local current density during the growth phase does not determine the morphology. Instead, the initial growth of new grains/branches could dictate which morphology evolves during their subsequent growth. To trigger different types of growth, changes/cracks in the SEI-layer could impact the nucleation of new growth sites. This electrolyte forms a roughly 10 nm thick, amorphous SEI,[35] containing several inorganic components, such as LiF, $Li_2S$, $Li_2O$ and $Li_3N$, as determined by X-ray photoelectron spectroscopy experiments (**Fig. S22, Supplementary Note 10**). This type of interphase can be expected to be brittle and thereby prone to crack due to stress buildup during growth.[36]

## Manuscript edits on page 10:

Transitions between sheet and needle morphologies can occur within one connected structure, and it is hypothesized that transitions are caused by changes/cracks in the SEI-layer impacting the initial growth of new lithium grains, but more work is required to fully understand the mechanism. For instance, the coupling between SEI layer properties and deposit morphology needs to be rationalized, calling for both systematic studies exploring the impact of current density and electrolyte formulation, to shed light on the role of the SEI by tuning its characteristics, as well as theoretical modelling based on 4D tomography datasets.

## Manuscript edits in Supporting information on page 29-30:

**Supplementary Note 9: Local current density estimate**

The local current density, $j$, in a region of the electrode can be calculated as

$$j = \frac{Q}{tA},$$

Where, $A$, is the area, $t$, is the time, and $Q$ is the charge passed to the electrode. Using Faraday's law, the expression can be rewritten as

$$j = \frac{\rho V n F}{tAM}.$$

Where $\rho$ is the density of lithium, $V$ is the volume of lithium deposited in the time $t$, $n$ is the number of electrons transferred in the reaction (1), $F$ is Faraday's constant, $M$ is the molar mass of lithium. With tomography images at two different times, $t_1$ and $t_2$, this formula can be used to estimate time-averaged local current density values at $t=(t_1+t_2)/2$ by determining the lithium volume increases, $V= V_2-V_1$, and average lithium surface areas $A=(A_1+A_2)/2$. **Fig. S21** shows this calculation for different segments of the lithium structure from Fig. 6. Calculations under two different assumptions are shown:

1. All of the surface area is active in the deposition, but lithium is redistributed to the growth zones through diffusion (Fig. S21a)
2. The lithium surface diffusion on the structures can be neglected, which implies that the lithium must be deposited in the growth zones allowing the current density to be estimated based on the surface areas of the growth zones. For the needle (segment 1) active areas at both ends of the needle are assumed, with a cross-sectional area of roughly 3.9 $\mu m^2$, a total active area of A=7.8 $\mu m^2$ is used for the calculation. For the

sheets (segment 3) the growth occurs along its edges, the surface of which can be manually defined in the tomography volumes (red in Fig. S21b).

Neither of these cases are likely to be fully representative of the deposition process but enable straightforward calculations of the local current density. For both cases, the current density shows a quick decline over time. Given that the deposition current is constant, it is inevitable that the current densities decrease when the surface area of the electrode increases through inhomogeneous deposition. This trend should also be exacerbated by the gradually increasing thickness/resistivity of the SEI as the structures age.

The largest local current density for any of these segments is significantly larger than the smallest current for the other segments. This could suggest that the local current density during the growth does not determine the morphology. A possible explanation for the vastly different morphologies could be that different nucleation events dictates which morphology evolves during subsequent growth, as suggested by Wang *et al.*[17]

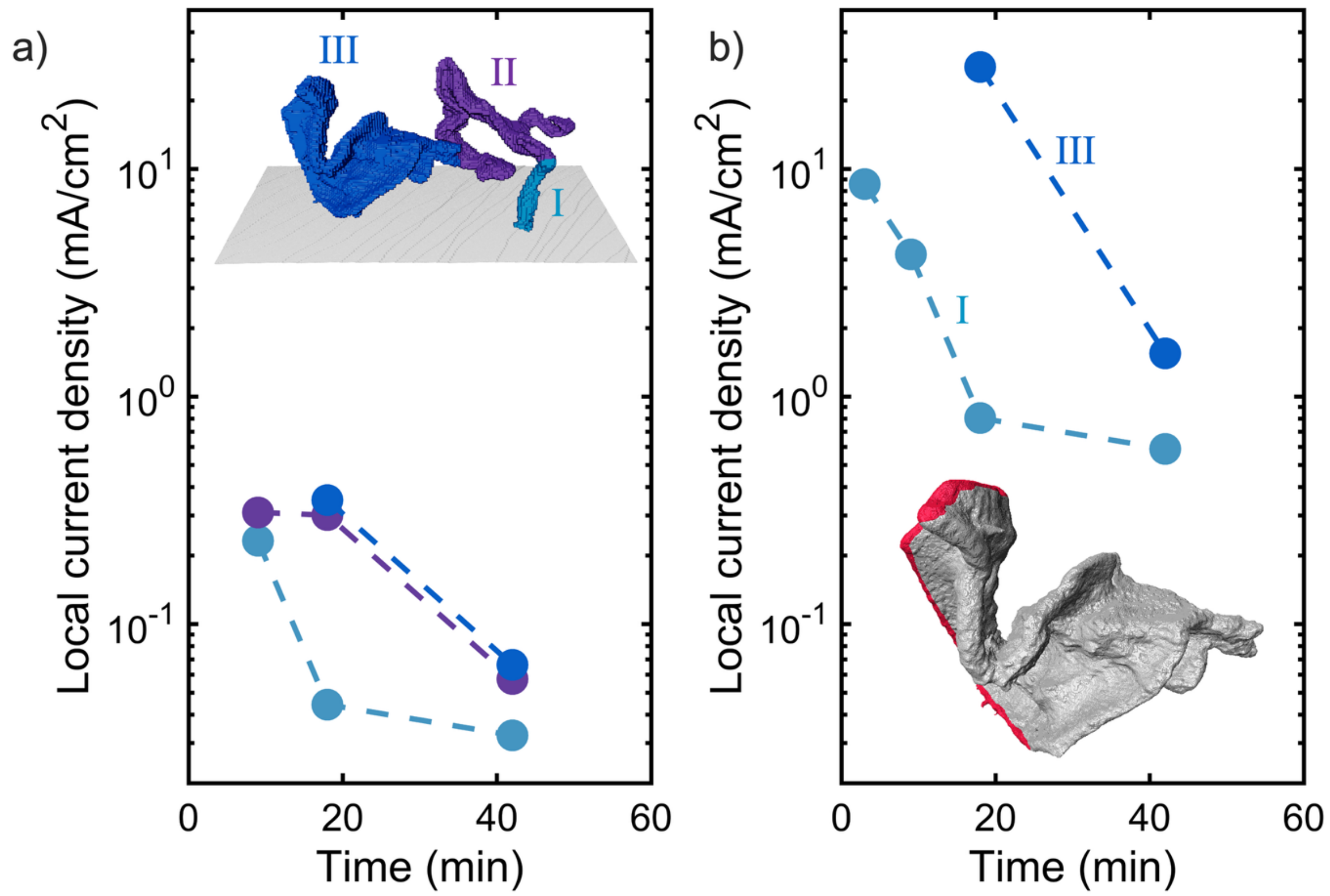


**Fig. S21**. Current density estimations. a) Current densities estimated for segment I, II and III of the structure (see inset), assuming their entire surface area is active during deposition. b) Current densities for segment I and III, calculated using assumptions regarding which areas are active during the deposition. Inset shows active area defined for segment 3 after 60 min of deposition.

## <u>Response to Reviewer #4:</u>

*The authors present their work which demonstrates time-resolved, X-ray, holo-tomography of Li-dendrite growth inside of a Li-ion battery. Through this they are able to elucidate on various growth modes for Li dendrites during cycling. Notably, they claim that this is the first time-resolved tomography study on Li-dendrite growth. The direct impact is somewhat limited by the use of a 100 μm electrode gap which may alter certain effects to Li-plating (i.e., ion transport effects) as well as relegate some of the information irrelevant because li-dendrites are not allowed to grow to this size or thickness in standard cells were separators rarely extend beyond 30 μm in thickness. Regardless, I believe this work to be highly novel and an excellent addition to the literature after minor or optional revisions and/or explanations. I would mainly like to see comments 1 and 2 addressed in the manuscript*

*1) I would like to see comments added to the manuscript discussing the 100 μm*

*electrode gap and its possible consequences:*
*a. Why was such a large gap used? (Experimental requirement?)*

**Authors' response:** In this work we wanted to investigate the fundamental aspects of lithium deposition. External pressure and a separator add complexity both to the experimental setup and make it more difficult to disentangle which factor influences what part of the deposition process. Further, the segmentation is more difficult with additional components. To ensure that cells did not short circuit under the chosen conditions, the gap had to be large, here set to 1 mm (note: not 100 µm) to be on the safe side. We have clarified this in the discussion on cell configuration in supporting information, page 6.

**Manuscript edits in supporting information on page 6:**
**Supplementary Note 3: Cell configuration**
The tomography cell operates with no applied stack pressure, and a large electrode separation (≈1 mm). This ensures that cells do not short circuit while keeping electrode separation small enough to avoid ion depletion at the electrode surface (see Supplementary Note 4). Still, the absence of stack pressure is a clear difference between this cell and common lithium metal cell formats, where the pressure and type of separator have a strong influence on the morphology of the deposited lithium.[5] Generally, a more compact morphology with larger grains form under pressure.[6] However, the deposition without pressure is still important to understand for two reasons: 1) the driving forces controlling the lithium metal deposition are easier to disentangle in this more idealized scenario and 2) local differences in pressure are common in both coin and pouch cells,[7–9] which means that it is important to understand how lithium grows also in regions with little or no pressure.

*b. More importantly, how can this effect Li-plating and dendrite growth mechanisms? For example, the authors mention in the introduction that branched fractal structures form due to ion-transport limitations. I would assume a possible explanation why these structures are not seen here is that ion-transport was not a significant factor for this experiment because the "separator thickness" was so large. For example, 10 µm of lithium growth with a 100 µm gap reduces the ion-transport distance by 10% where as a more practical 15 µm gap would see transport reduced by 66%.*
*c. Why don't you see "classical dendrites" in this experiment?*

**Authors' response:** The diffusion limited current density, $J^*$, above which the ion diffusion to the electrode cannot be sustained, eventually causing the ion surface concentration to fall to 0, can be calculated from [1 (eq S9)]

$$J^* = \frac{4z_{Li}c_0FD_{Li}}{L}$$

where, $z_{Li}$ is the charge number of the Li ion (1), $c_0$ the bulk salt concentration, $F$ is Faraday's constant, $D_{Li}$ the diffusion coefficient for Li ions in the electrolyte and $L$ the inter-electrode separation. The distance between the electrodes sets the maximum possible thickness of the diffusion layer, a larger separation leads to a thicker diffusion layer and thus reduced diffusive mass transport. In practical batteries, the limiting current density will be much higher than the currents applied (due to the small electrode separation). For our electrolyte, $D_{Li} = 2.06\ 10^{-6} cm^2/s$ was determined using nuclear magnetic resonance spectroscopy at 20°C. Together with an electrode spacing $L = 1$ mm, and 4 M Li-ion concentration, this would give a limiting current density $J^* = 3.2\ \mathrm{mA/cm^2}$. This value is significantly higher than the current density in this work. Therefore, even if ion diffusion limitations are more pronounced in this cell, compared to a standard cell with smaller electrode spacing, we are far from a region where we risk depleting the electrolyte, leading to the formation of classical dendrites (fractal structures). This corresponds well to the absence of fractal structures in our experiment.

We have clarified this point on page 2 in the manuscript and page 7 in supplementary information.

**References**

[1] P. Bai, J. Li, F.R. Brushett, M.Z. Bazant, Transition of lithium growth mechanisms in liquid electrolytes, Energy Environ Sci 9 (2016) 3221–3229. https://doi.org/10.1039/c6ee01674j.

**Manuscript edits on page 2:**

This morphology stands in contrast to both (i) classical dendrites, which are fractal self-similar structures forming due to ion depletion in the electrolyte (**Supplementary Note 4**),[9,22] as well as (ii) ideal bulk lithium. The applied current is too low to cause ion depletion with the cell geometry and electrolyte used, explaining the absence of fractal structures (Supplementary Note 4).

**Manuscript edits in supporting information on page 7:**

**Supplementary Note 4: Dendrite growth**

The absence of fractal, self-similar structures (classical dendrites), can be rationalized considering the moderate current density during deposition. The diffusion limited current density, $J^*$, above which the ion diffusion to the electrode cannot be sustained by the electrolyte, eventually triggering fractal (dendritic) growth, can be calculated from the following formula:[10]

$$J^* = \frac{4 z_{Li} c_0 F D_{Li}}{L}$$

where, $z_{Li}$ is the charge number of the Li ion (1), $c_0$ the bulk salt concentration, $F$ is Faraday's constant, $D_{Li}$ the diffusion coefficient for Li ions in the electrolyte and $L$ the inter-electrode separation. The diffusion coefficient $D_{Li} = 2.06\ 10^{-6} \mathrm{cm}^2/\mathrm{s}$ for this electrolyte (determined using nuclear magnetic resonance spectroscopy at 20°C), together with an electrode spacing $L = 1$ mm, and 4 M Li-ion concentration gives a limiting current density $J^* = 3.2\ \mathrm{mA/cm}^2$. This value is significantly higher than the current applied in this work, explaining why classic (fractal) dendrites could not be observed.

*2) The authors note that Li-deposition is concentrated where the SEI is least resistive (i.e., thinnest). They further note that at OCV, cell impedance increases and that it requires 12 min to acquire a tomogram. What effects, if any, do you think this has on SEI? Would you expect Li growth to look substantially different had the tomograms been able to be acquired instantaneously?*

**Authors' response:** It is true that the SEI thickness is expected to increase when the cycling is interrupted and the cell is rested at OCV. However, the characteristic morphology with needles and sheets is also observed in experiments without OCV holds, as shown in the complementary SEM analysis (figure S5). Moreover, the overall resistance of the electrode increases slowly during an OCV hold (Figure S18), but since the same areas of the electrode are still likely to be the least resistive both before and after the hold, this should not have a strong influence on the overall morphology.

**Manuscript edits on page 14**:

Since the characteristic growth modes of needle and sheet were confirmed to arise also in cells cycled without interruption (Fig. S5, SEM images), the morphology should not have been qualitatively altered by the interruptions, and ion depletion should not occur at the electrode with or without pauses (Supplementary Note 4).

*3) Mention cell shorting as an effect t of Li-dendrites in the intro.*

**Authors' response:** We have added a comment regarding risk of short circuiting in the introduction.

**Manuscript edits on page 1**:

Ideally, the plated Li metal would form a dense and uniform film,[3] but in practice, it tends to plate and strip heterogeneously, resulting in low reversibility and risks short circuiting the cell.

*4) Why do you make the distinction between closed loops and connected Li structures? Shouldn't all connected structures also be closed loops because they all originate from the same native Li Layer?*
*5) Does growth of Li structures stop after forming a closed loop form? I.e., is the loop actually connected electronically or does the thicker and more resistive SEI prevent electrical connection when the loop is formed? Do you have enough data to monitor the evolution of loop structures or only enough the see that they eventually form.*

**Authors' response:** We are distinguishing between closed loop (where you can return to your starting point without traversing the same path twice) from the larger class of connected structures because they could imply different growth modes. All deposited Li structures should be connected to the bulk electrode, since the electrons needed for the deposition could not be provided otherwise. However, if the bulk electrode is removed, and there are still closed loops in the structure, this could indicate that branches coalesce during growth. This has been clarified on page 7.

We show in Fig. S15 that connections between structures found using tomography are not always stable over the growth, which highlights that at the individual lithium structures in at least some cases are not actually attached but only in close proximity, suggesting we would not have an electronic connection. However, at this point we do not have enough data to ascertain whether this is always the case or if there could be instances where branches permanently coalesce. We have clarified on page 7 that this remains an open question.

**Manuscript edits on page 7:**
Still, the possibility of different branches coalescing during deposition remains an open question. Several closed loops are found in the analyzed volume (**Fig. S16**), which could be a sign of coalescence, but this data is not sufficient to determine if the loops generally stay connected during continued growth.

*6) Explain why the Li-layer gets thicker while simultaneously getting more dense near the CC side. Shouldn't this conflict with the conclusion that there is limited growth on older Li structures due to a thicker and more resistive SEI?*

**Authors' response:** All the structures close to the electrode are not old. Due to formation of new branches and that structures can grow along and/or towards the bulk electrode (see for instance Fig. 6a), we can still have rapid growth of structures close to the bulk electrode. This has been clarified on page 8.

**Manuscript edits on page 8:**
Note that the fast growing freshly formed structures can both be close to and far from the bulk electrode since new branches can grow in many different directions, as for instance illustrated by the growth of the structure in Figure 6a.

*7) Can you elaborate on the needle-to-sheet transition in growth modes? Did you observe enough of these transitions to identify any patterns or indicators for when this transition occurs?*

**Authors' response:** We observe no cases where a sheet grows directly from the bulk electrode. But see several cases where needles transition to sheets and also back again. Sheets grow both from the side of needles and from their tip. We have added another

example of this kind of transition in Fig. S7. To shed light on this transition we have also analyzed the local current densities during the growth of a Li sheet and a Li needle, establishing that there is significant overlap between their local current densities. Further, electrochemical simulations have also been carried out (Fig. 4), establishing that the differences between sheet and needle growth cannot arise due to ion-concentration differences in the electrolyte or electric field effects. Instead, the transitions between different growth modes need to be triggered by for instance differences in interfacial resistance, which is not captured by the simulation.

Wang *et al.* [1] propose that the initial growth event/nucleation, and particularly the crystallinity of the structure, is crucial for determining the final morphology. With this mechanism in mind a sheet would arise after a slow nucleation event, while not excluding that after the initial growth, the sheet could grow more quickly than, or at the same rate as, the needle.

We have clarified these points on pages 6 and 8-9 in the revised manuscript. However, we also emphasize in the manuscript that the mechanism for the transition to sheet growth needs further investigation in the conclusions section on page 10.

**References:**

[1] Wang, X. *et al.* Glassy Li metal anode for high-performance rechargeable Li batteries. *Nat. Mater.* **19**, 1339–1345 (2020).

**Manuscript edits on page 6:**

To elucidate the growth mechanism, the equilibrium Li-ion concentration and local current densities were obtained from electrochemical simulations based on the actual 3D-structures obtained from tomography. (**Fig. 4, Fig. S11-S13, Supplementary Note 7**). Assuming a homogeneous interfacial resistance across the electrode, a Li ion concentration gradient from the top of the sample to the bulk electrode is predicted, with smooth variations and no significant changes around edges or endpoints (Fig. 4a, c). This ion concentration gradient would cause slightly higher Butler-Volmer exchange current density at the structures extending the furthest into the electrolyte (Fig. 4b, d), but generally the variations are small, less than 10 %. Clearly, neither the differences between needles and sheets or the emergence of either shape, can be due to enhancement of electrical fields at tips or Li concentration gradients. Instead, effects such as crystal anisotropy and differences in interfacial resistance across the electrode, not accounted for in the simulation, must be driving the evolution of the electrode morphology.

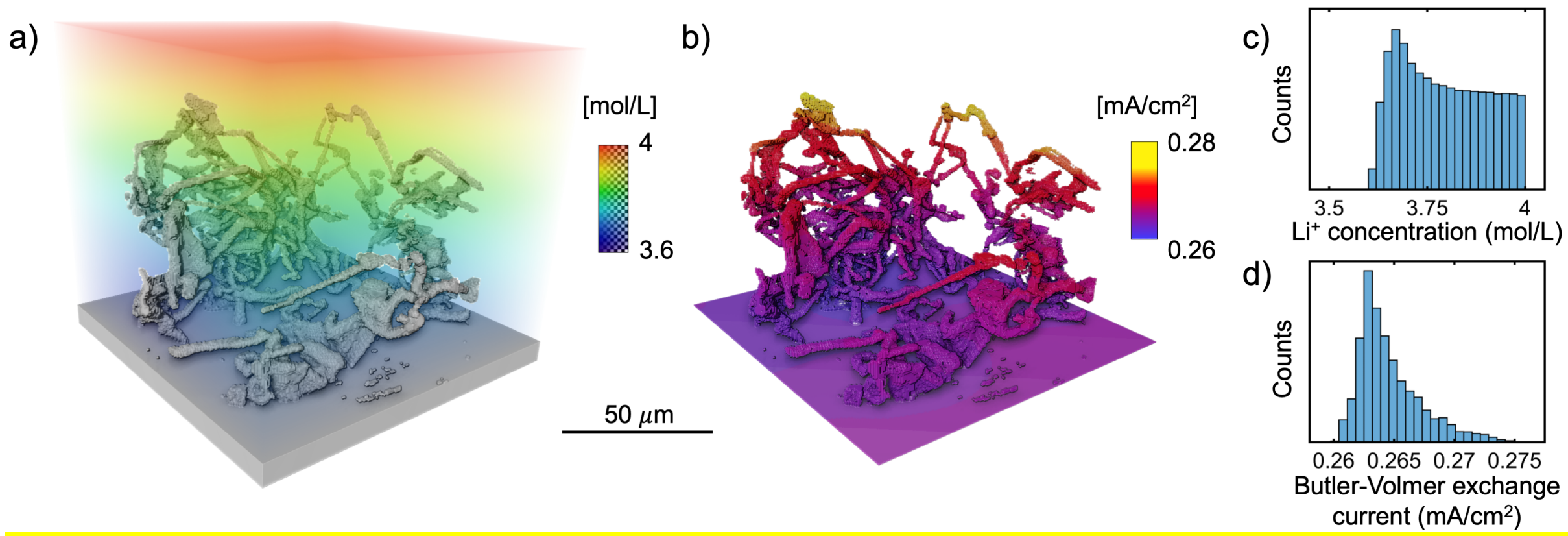


**Fig. 4**. 3D electrochemical simulations, based on Li morphology after 24 min. a) Li-ion distribution in the electrolyte, visualized with a semitransparent colormap. Grey structures correspond to the Li metal. b) Local Butler-Volmer exchange current density distribution across the electrode surface. c-d) Histograms based on the data in a-b.

**Manuscript edits on page 8-9**:

The detailed analysis of individual deposits also highlights transitions between different dimensionalities, e.g. from 1D (needle-type) to 2D (sheet-type) segments during deposition. For instance, **Fig. 7a** and Fig. S7 show sheets growing from the side of a needle, expanding in-plane. Similarly, **Fig. 7b** shows a sheet formed at the tip of a needle. The converse transition of growth mode, from sheet to needle, can also be observed (see **Fig. S20**), however no instances of sheets growing directly from the bulk electrode could be found. The range of local current densities estimated for growing sheets and needles show significant overlap (**Fig. S21, Supplementary Note 9**), suggesting that local current density during the growth phase does not determine the morphology. Instead, the initial growth of new grains/branches could dictate which morphology evolves during their subsequent growth. To trigger different types of growth, changes/cracks in the SEI-layer could impact the nucleation of new growth sites. This electrolyte forms a roughly 10 nm thick, amorphous SEI,[35] containing several inorganic components, such as LiF, $Li_2S$, $Li_2O$ and $Li_3N$, as determined by X-ray photoelectron spectroscopy experiments (**Fig. S22, Supplementary Note 10**). This type of interphase can be expected to be brittle and thereby prone to crack due to stress buildup during growth.[36]

**Manuscript edits on page 10:**

Transitions between sheet and needle morphologies can occur within one connected structure, and it is hypothesized that transitions are caused by changes/cracks in the SEI-layer impacting the initial growth of new lithium grains, but more work is required to fully understand the mechanism. For instance, the coupling between SEI layer properties and deposit morphology needs to be rationalized, calling for both systematic studies exploring the impact of current density and electrolyte formulation, to shed light on the role of the SEI by tuning its characteristics, as well as theoretical modelling based on 4D tomography datasets.

*8) Did the cell ever electrically short?*

**Authors' response:** Not under the conditions used at the synchrotron. Shorts were avoided through the large electrode separation and limited current and areal capacity.

*9) While likely outside the scope of work, given this was a synchrotron experiment, I would be interested to see future work where the experiment is extended to stripping, both of the native Li layer as well as stripping of plated dendrite features. Would stripping also be localized to the red areas in fig 6 where the SEI is assumed thinnest? Would an SEI shell be left behind and subsequently affect additional plating? Interesting questions.*

**Authors' response:** These are indeed interesting points. We agree that stripping is one of many important phenomena regarding metal anodes that could and should be covered in future work using nano-holo-tomography. We have added a sentence to the conclusion on page 10 to highlight this.

**Manuscript edits on page 10**:

More broadly, *in situ* X-ray nano-tomography experiments could help shed light on a wide range of processes influencing the morphology of lithium metal electrodes such as stripping or cycling under pressure.

# A Point-by-Point Response to Reviewers comments on manuscript ID NCOMMS-25-62476-T

**Manuscript ID:** NCOMMS-25-62476-T

**Title:** Revealing Li growth modes using X-ray nano-tomography

**Authors:** Josef Rizell, Victor Vanpeene, Martina Olsson, Jan Jamroz, François Cadiou, Hervé Manzanarez, Valentin Vinci, Olga Stamati, Julie Villanova, Sandrine Lyonnard, Aleksandar Matic

**<u>Response to Reviewer #2:</u>**

*The authors have improved their manuscript considerably. They strengthened the line of arguments by additional evaluations and clarified their conclusions. Therefore, the manuscript can be accepted for publication after some minor improvements.*

*These minor modifications are suggested:*

*p. 1.*
*and risks of short circuiting the cell.*
*instead of*
*and risks short circuiting the cell.*

*through which the Li-ions must be transported to reach the electrode*
*instead of*
*which the Li-ions must be transported through to reach the electrode*

*cracks in the SEI result in hotspots for deposition*
*The authors might consider to add an additional reference here, strongly supporting the "cracks causes dendrites" hypothesis:*
*Doron Aurbach, Ella Zinigrad, Yaron Cohen, Hanan Teller,*
*A short review of failure mechanisms of lithium metal and lithiated graphite anodes in liquid electrolyte solutions*
*Solid State Ionics 148 (2002) 405– 416*

*Supplementary Note 4:*
*$z_{Li}$ is the charge number of the Li ion ($z_{Li}$ = 1),*
*instead of*
*$z_{Li}$ is the charge number of the Li ion (1),*

*Supplementary Note 9:*
*Since several time steps are considered, the equations should rather read*
*$j = \Delta Q / A \Delta t$*
*instead of*
*$j = Q / t A$*
*and*
*$j = \rho \Delta V n F / \Delta t A M$*

*instead of*
*j = ρ V n F / t A M.*
*Since the variables are written in italics in the equations, variables in the following text should also be in italics (times, $t_1$ and $t_2$, $t=(t_1+t_2)/2$, $\Delta V= V_2-V_1$, $A=(A_1+A_2)/2$.*

*To emphasize that lithium atoms diffuse on the surface, one might write*
*but lithium atoms are redistributed to the growth zones through diffusion*
*instead of*
*but lithium is redistributed to the growth zones through diffusion*
*and*
*The surface diffusion of lithium atoms on the structures*
*instead of*
*The lithium surface diffusion on the structures*

*Since the publisher will not edit the supplementary material, is suggested to check and to improve the typesetting of the equations. Usually, functions like sinh and operators like div and grad are not in italic (e.g. \sinh might be used in LaTeX).*
*In the text,*
*Equation (1)*
*might be used instead*
*Equation 1*
*(correspondingly for all links to the equations).*
*Usually, equation numbers are not in italics.*
*In the lines*
*η is the overpotential η = E - Eeq = ϕS - ϕel for the lithium*
*and*
*Making the assumption that α = 1 - β = 0.5*
*please use minus signs instead of the dashes.*

**Authors' response:**
We thank the reviewer for the comments and have implemented the suggested changes in the revised manuscript.

## Response to Reviewer #3:

*The authors have addressed the questions and concerns in this revision. The manuscript has been improved. Although a new characterization method was used, similar findings have been reported, reducing the novelty of the work. In addition, several critical questions should be well addressed.*

**Authors' response:**
We thank the reviewer for the constructive feedback which we have addressed in the subsequent comments.

*1. Regarding the novelty of the work: several studies on the Li morphology evolution and the mechanism underlying have been reported at nano-meter spatial resolution and/or under in-situ conditions. What are the differences of the Li deposition mechanism that are obtained from this work compared to previous reports (Nano Energy 32 (2017) 271–279; ACS Energy letters, 2021, 6, 1719-1728.)?*

**Authors' response:**
We thank the reviewer for highlighting the work of Kushima *et al.* (Nano Energy 32, 2017, 271–279) [1] and Dong *et al.* (ACS Energy letters, 2021, 6, 1719-1728) [2] and below we detail how our work distinguishes from previous reports. With this we further underline the novelty of our work and how it advances the understanding of Li metal deposition.

As noted by Kushima *et al.* lithium deposition can proceed through different growth regimes, including root growth of needle-like structures at lower overpotentials and surface growth of clusters at higher overpotentials. Their TEM observations over timescales of a few minutes showed evidence of lithium needles growing from their base, where new lithium segments are extruded from the bulk electrode, beneath existing deposits, likely through cracks in the SEI. In contrast, our *in situ* tomography measurements track a larger and more representative 3D volume over a much longer timescale (1 h) allowing us to follow the evolution of the structure not possible before. This is an intrinsic benefit of using X-ray tomography, compared to TEM, and this has been clarified on page 2 and a reference to the work of Kushima has been added on page 4. This allows us to observe that all parts of the lithium deposit continue to grow simultaneously, with the newly formed segments exhibiting the highest growth rates. This behavior differs from the sequential segment-by-segment growth mechanism proposed by Kushima *et al.* and further underlines that needle growth is not only driven by extrusion from the base of the structure. A discussion on this has been added on page 8. By probing a larger volume, due to the larger field of view in X-ray tomography, we are also able to observe different types of lithium (sheets and needles) coexisting and transitions between the two growth modes occurring. We have revised the conclusion on page 10 to emphasize the importance of a large FOV.

Dong *et al.* propose that Li balls, primarily amorphous, form at low current densities while bush shaped deposits (consisting of balls, whiskers and bulky Li) form at higher current densities. A reference to this work has been added on page 4 in the discussion on amorphous lithium deposition. However, Dong *et al.* observe significantly different morphologies compared to those reported in our study. We believe that this reflects a different deposition regime, controlled by the electrode separation and choice of electrolyte and applied current. Chae *et al.* [3] report similar morphologies to Dong *et al.*, for large electrode separations but with the smaller electrode spacing, both sheets and needles are observed, resembling the morphology observed in our work. We have added a discussion on this in Supplementary note 3 on page 6 of the supporting information. The morphological evolution in our work is not driven by ion diffusion limitations in the electrolyte (see Fig. 4 and supplementary note 4), which is the case by Dong et al, instead crystallinity, crystal anisotropy and interphase evolution are proposed as key drivers. We have developed the discussion on this in the conclusions on page 10.

**References:**

[1] Kushima, A. *et al.* Liquid cell transmission electron microscopy observation of lithium metal growth and dissolution: Root growth, dead lithium and lithium flotsams. *Nano Energy* **32**, 271–279 (2017).
[2] Dong, K. *et al.* Unravelling the Mechanism of Lithium Nucleation and Growth and the Interaction with the Solid Electrolyte Interface. *ACS Energy Lett.* **6**, 1719–1728 (2021).
[3] Chae, O. B., Kim, J. & Lucht, B. L. Modification of lithium electrodeposition behavior by variation of electrode distance. *J. Power Sources* **532**, 231338 (2022).


**Manuscript edits on page 2:**

While TEM provides excellent spatial resolution, the cell configuration is far from ideal only allowing very thin two-dimensional structures in a small field of view to be tracked. This limits investigations of lithium deposition to relatively short timescales, as structures soon start growing outside the field of view.

**Manuscript edits on page 4:**

(ii) Inhomogeneous deposition beneath the SEI layer also creates stress and, eventually, the SEI layer cracks and lithium metal is extruded through the crack as a needle.[12,18]

**Manuscript edits on page 4:**

A sheet-like morphology has been associated with non-crystalline deposition[37] and amorphous lithium deposits have previously been observed in TEM experiments.[38,39]

**Manuscript edits on page 8:**

This stands in contrast to the sequential growth of needle segments through extrusion from the base proposed by Kushima *et al.*[18], underlining that needle growth occurs not only from the base, but rather through concurrent growth of connected segments, proposed for instance by Steiger *et al*.[16] The fast growth of newly formed segments points to preferential deposition on freshly formed structures, where the SEI-layer is expected to be thin and highly conductive.

**Manuscript edits on page 10:**

A large field of view (102×102×102 $\mu m^3$) allowed the evolution of lithium structures to be tracked over a longer time (1h of deposition) as the growing structures stay within the field of view.

**Manuscript edits on page 10:**

Based on our data, the evolution of these structures cannot be explained by ion diffusion limitations in the electrolyte, instead we propose a model for Li growth, where Li structures also grow with almost constant thickness, through branching, elongation of existing branches and planar growth of sheets.

**Manuscript edits in supporting information on page 6:**

The tomography cell operates with no applied stack pressure, and an electrode separation around 1 mm. This ensures that cells do not short circuit while keeping electrode separation small enough to avoid ion depletion at the electrode surface (see Supplementary Note 4). Note that very large electrode separations have been observed to trigger spherical lithium growth morphologies, whereas more moderate inter-electrode spacing has been shown to result in morphologies similar to those observed in this work.[5]

*2. The authors state that no gas was observed in the experiments. Images at the initial stage and at the end of the experiment are suggested to be provided in the SI.*

**Authors' response:**

Cross-sectional slices from reconstructed tomograms at the initial and final stage of the experiment have been added to the supporting information on page 34, supporting that bubble formation due to gas evolution was not detected.

**Manuscript edits on page 15:**

Using these settings, no bubble formation, indicative of beam-induced electrolysis, was observed during the experiment (see **Fig. S23**).

**Manuscript edits in supporting information on page 34:**

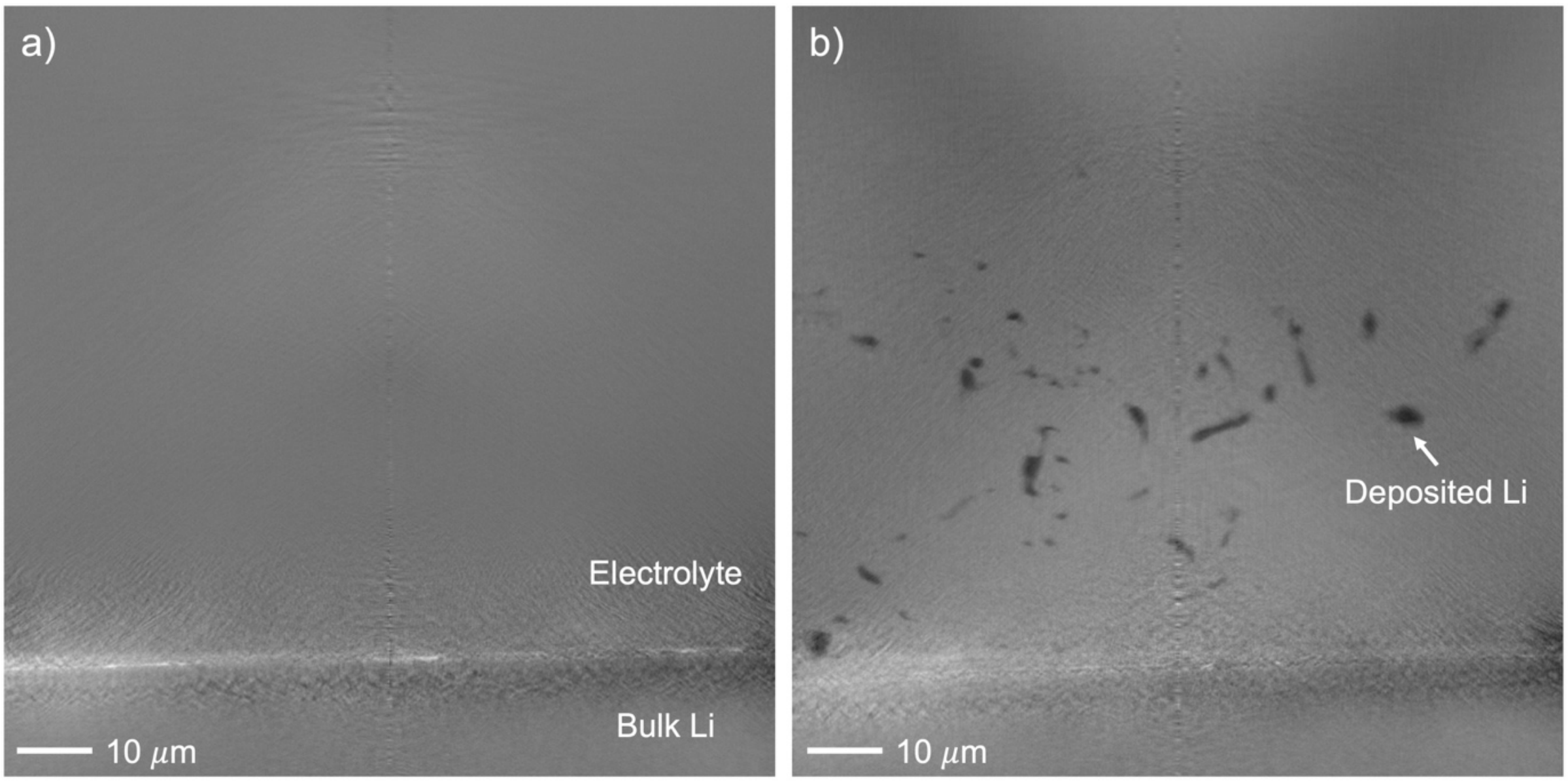


**Fig. S23**. Cross-sectional slices through volumes reconstructed from 50 nm voxel size holo-tomography measurements before (a) and after (b) Li electrodeposition (1 mA/cm$^2$ for 1 h).

*3. The in-situ cell has a diameter of 1.6 mm. But a field of view around 100 µm was shown in the manuscript. Which part of the in-situ cell is shown in the work? As lithium-ion diffusion could be different in different areas in the cell and thus lead to varying deposition morphology, it is therefore not clear if the selected field of view shown in the paper representative? How about the lithium deposition at the other regions within the cell?*

**Authors' response:**

To avoid edge effects, the central part of the electrode was imaged. We have added schematic drawing in supporting information (new figure S25), showing the part of the electrode which was studied. The representativity of the 102×102×102 µm$^3$ subregion imaged with high spatial resolution (see for instance Fig. 1b and 2b) can be assessed by analyzing the surrounding 526×526×526 µm$^3$ micrometer region, which was imaged with lower spatial resolution (see Fig. 1a, new figure S25). The lithium morphology remains qualitatively the same in the 526×526×526 µm$^3$ region imaged.

**Manuscript edits on page 16:**

The center of the electrode was imaged during the experiment and the representativity of the (102×102×102 µm$^3$) subregion tracked with high resolution X-ray nano-holo-tomography measurements was confirmed by comparing the lithium morphology there to surrounding locations on the electrode (**Fig. S25**). Qualitatively, these show similar morphologies.

**Manuscript edits in supporting information on page 36:**

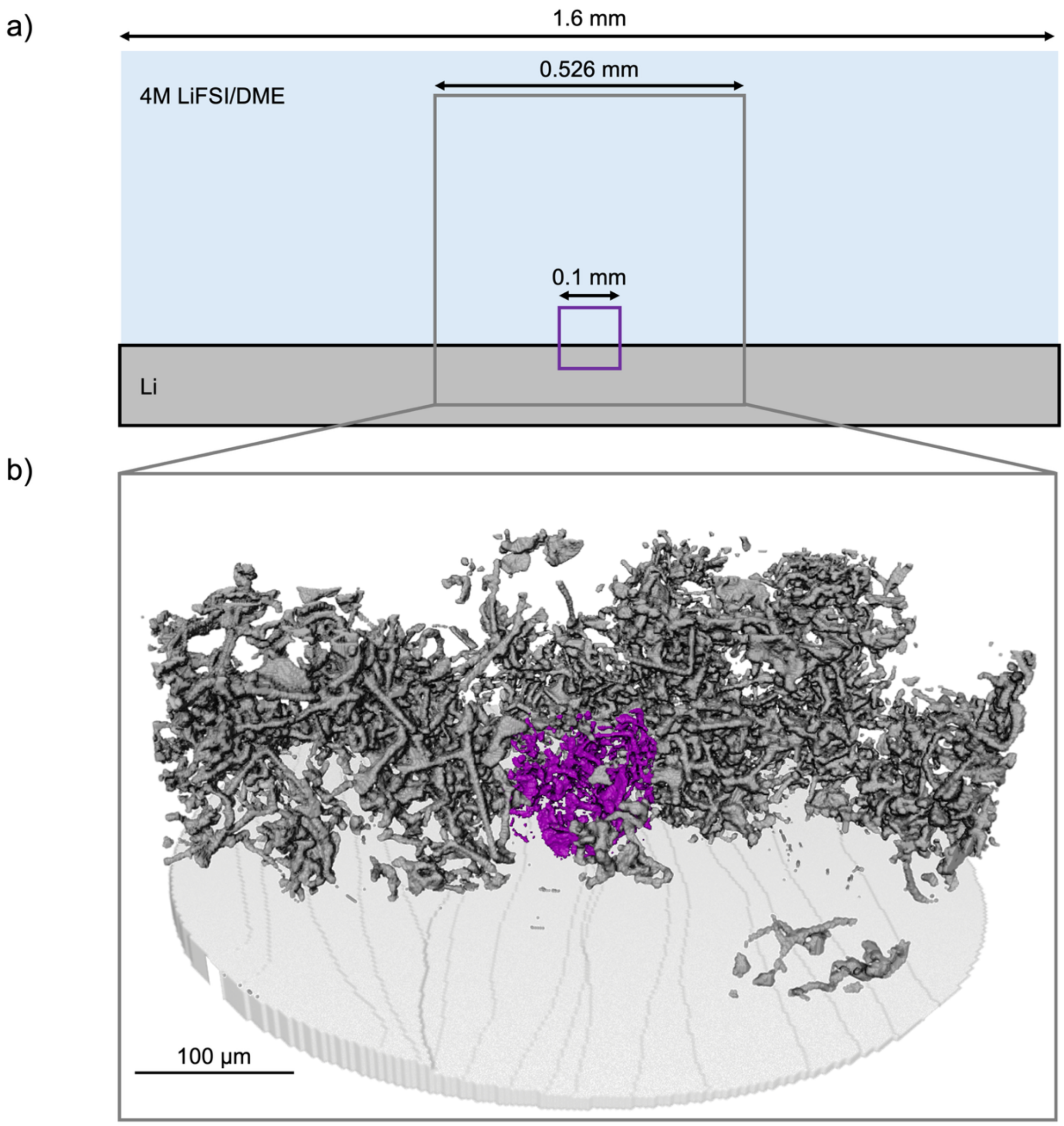


**Fig. S25**. Field-of-view during imaging of the lithium electrode a) Schematic drawing, with a grey square representing the volume imaged in overview measurements, and a purple square representing high resolution nano-holo-tomography. b) Volume rendering of segmented Li, after deposition at 1 mA/cm$^2$ for 1 h, from *in situ* nano-tomography measurements at 257 nm voxel size (from Fig. 1a). Region in the center of the volume where high resolution (50 nm, voxel size) X-ray nano-holo-tomography scans were acquired is shown in purple.

*4. As the lithium deposition behavior differs significantly under different chemical and pressure conditions. A concentration of 4M LiFSI/DME electrolyte and a zero-pressure setup that were used in the experiment are rarely used in lithium battery research. (The limitation of the setup was also mentioned by reviewer #1.) Therefore, these two limitations greatly lower the impact of the phenomenon observed and the results obtained in this work. To ensure the novelty of the work, trial experiments using in-situ setups with pressure or widely used electrolytes (like 1M LiPF6/EC+DEC, 1M LiTFSI/DOL+DME, etc.) should be added.*

**Authors' response:**

Cell configuration and applied pressure can impact deposited Li morphology, as discussed in response to referee 1 in the previous revision round and the supplementary note 3 added in that revision. To further clarify the significance of analyzing Li deposition in a pressure free

environment we have added a discussion on page 2 in the revised manuscript. Studying the detailed effects of pressure is of course of high interest but is outside the scope of this work and a very interesting direction for future studies as we also note in the conclusions section on page 11.

Concerning the choice of electrolyte, it is interesting to investigate the lithium morphology during electrodeposition in highly concentrated electrolytes, since they have generally shown good compatibility with Li metal electrodes, increasing the coulombic efficiency and resulting in longer cycle life. [1-2] The 4M LiFSI/DME electrolyte used in this work has been shown to support excellent long term cycling performance of Li-metal cells (shown for instance by Quian et al [3]). Adding to the relevance of the study and its conclusions, similar morphologies to what we report in our work have also been observed in more standard electrolytes with lower salt concentration (typically 1M). To clarify these points and highlight the relevance of our results we have added a discussion on the choice of electrolyte and comparison to other systems on page 12 in supplementary information as Supplementary note 5 and added a comment on page 4 stating that similar structures can be found also with other electrolytes. With this in mind, we think that the presented results are relevant and interesting without additional tomography data from other electrolytes. A detailed characterization and comparison of morphologies formed in different electrolytes is indeed interesting, which we point out in the conclusions section, but it falls outside the scope of the current work.

**References**

[1] Suo, L. *et al.* Fluorine-donating electrolytes enable highly reversible 5-V-class Li metal batteries. *Proc. Natl. Acad. Sci. U. S. A.* **115**, 1156–1161 (2018).
[2] Fan, X. *et al.* Highly Fluorinated Interphases Enable High-Voltage Li-Metal Batteries. *Chem* **4**, 174–185 (2018).
[3] Qian, J., Henderson, W., Xu, W. et al. High rate and stable cycling of lithium metal anode. Nat Commun 6, 6362 (2015).

**Manuscript edits on page 2:**

Measuring without applied pressure constitutes an idealized case, or model system, simplifying the analysis of the driving forces controlling the evolution of different morphologies during lithium deposition. Furthermore, local differences in pressure are common in both coin and pouch cells,[23–25] and it is thus important to understand how lithium grows also in regions with little or no pressure.

**Manuscript edits in supporting information on page 12:**

**Supplementary Note 5: Electrolyte formulation**

The 4M LiFSI/DME electrolyte used shows excellent long term cycling performance of Li-metal cells, as shown in for instance by Qian *et al.*[12] This is in line with the general observation that highly concentrated electrolytes shown good compatibility with Li metal electrodes, increasing the coulombic efficiency and resulting in longer cycle life.[13,14]

Even though a highly concentrated electrolyte is used in this work, the results are relevant also with respect to standard electrolytes with lower salt concentration (typically 1M). Electrodeposited Li with a morphology containing sheets and needles can be observed also in these types of electrolytes and some examples are listed in Table 1.

**Table 1.** Reports of lithium with sheet morphology.

| **Electrolyte** | **Reference** | **Figure** | **Morphology** |
|---|---|---|---|

| | | | |
|---|---|---|---|
| 1M $LiPF_6$ in EC:EMC 3:7 (v/v) | [5] | S9 | Needle + Sheet |
| 1M LiTFSI in DOL/DME | [15] | S4c | Needle + Sheet |
| 1M LiFSI in FDMB | [16] | 6e | Needle + Sheet |
| 1M $LiPF_6$ in EC:DMC (v:v = 1:1) | [17] | 1e | Sheet |
| 1M LiTFSI in EC-EMC (4:6 by wt.) | [18] | S9b | Needle + Sheet |

**Manuscript edits on page 4:**
Reports discussing sheet-like Li deposits are uncommon in literature, but several publications show SEM images of similar structures in a range of electrolytes (**Supplementary Note 5**) without discussing them in detail.[31–36]
